# Developing Global Maps of the Dominant *Anopheles* Vectors of Human Malaria

**DOI:** 10.1371/journal.pmed.1000209

**Published:** 2010-02-09

**Authors:** Simon I. Hay, Marianne E. Sinka, Robi M. Okara, Caroline W. Kabaria, Philip M. Mbithi, Carolynn C. Tago, David Benz, Peter W. Gething, Rosalind E. Howes, Anand P. Patil, William H. Temperley, Michael J. Bangs, Theeraphap Chareonviriyaphap, Iqbal R. F. Elyazar, Ralph E. Harbach, Janet Hemingway, Sylvie Manguin, Charles M. Mbogo, Yasmin Rubio-Palis, H. Charles J Godfray

**Affiliations:** 1 Spatial Ecology and Epidemiology Group, Department of Zoology, University of Oxford, Oxford, United Kingdom; 2 Malaria Public Health and Epidemiology Group, KEMRI/Wellcome Trust Programme, Centre for Geographic Medicine Research, Nairobi, Kenya; 3 Public Health and Malaria Control Department, PT Freeport Indonesia, Kuala Kencana, Papua, Indonesia; 4 Department of Entomology, Faculty of Agriculture, Kasetsart University, Bangkok, Thailand; 5 Eijkman-Oxford Clinical Research Unit, Jakarta, Indonesia; 6 Department of Entomology, The Natural History Museum, London, United Kingdom; 7 Liverpool School of Tropical Medicine, Liverpool, United Kingdom; 8 Laboratoire d'Immuno-Physiopathologie Virale et Moleculaire, IRD/Université Montpellier I, Faculté de Pharmacie, Montpellier, France; 9 KEMRI/Wellcome Trust Programme, Centre for Geographic Medicine Research - Coast, Kilifi, Kenya; 10 BIOMED, Universidad de Carabobo, Maracay, Venezuela

## Abstract

Simon Hay and colleagues describe how the Malaria Atlas Project has collated anopheline occurrence data to map the geographic distributions of the dominant mosquito vectors of human malaria.

## Introduction

Despite advances in mapping the geographical distribution and intensity of malaria transmission [1],[2], the ability to provide strategic, evidence-based advice for malaria control programmes remains constrained by the lack of range maps of the dominant *Anopheles* vectors of human malaria. This is because appropriate vector control depends on knowing both the distribution and epidemiological significance of *Anopheles* vectors [3]. Substantial investments by major donors in the distribution of long-lasting insecticide-treated nets and indoor residual spraying campaigns [4] are, therefore, not always fully informed by the basic biology of local anophelines.

Recent attempts to delineate *Anopheles* distributions have been conducted in Africa [5]–[11], the Americas [12]–[16], Europe [17], Central and South East Asia [18]–[22], and at the global scale [23]–[26]. The mapping techniques used in these various studies range from those based on expert opinion and simple interpolations to those employing more sophisticated statistical methods. Consequently, these studies are difficult to compare and impossible to synthesize globally. In addition, whereas in some regions *Anopheles* species distributions and their contribution to human malaria transmission are well known, uncertainty arises when suites of vectors contribute to local transmission, when the margins of the species ranges are poorly defined, and/or when there is simply a lack of any, or reliably identified, distribution records. Furthermore, as many regions attempt to maintain their malaria-free status against imported malaria [27] and others consider their prospects of malaria elimination [28],[29], contemporary maps of anophelines that are competent vectors for malaria are important in assessing local receptivity to reintroduction [30].

To help address these needs, the Malaria Atlas Project (MAP, http://www.map.ox.ac.uk) [31] has extended its activities to collate anopheline occurrence data to map the contemporary geographic distributions of the dominant mosquito vectors of human malaria. The plans for, and progress of, this initiative are described here.

## Defining the Dominant Anopheles Vectors of Human Malaria

There are 462 formally named *Anopheles* species, with a further 50 provisionally designated and awaiting description [32]–[34]. Of these, approximately 70 have been shown to be competent vectors of human malaria [35] and from this set, 52 candidate dominant vector species (DVS) were initially chosen for inclusion in the MAP vector distribution mapping project. These DVS are species (or species complexes) that transmit the majority of human malaria parasites in an area by virtue of their abundance, their propensity for feeding on humans, their mean adult longevity (only old individuals incubate the parasite long enough to transmit the disease), or any combination of these and other factors that increase overall vectorial capacity [36]. The DVS were the inclusive set of those species identified as “main” [37],[38], “dominant” [24], or “principal” [23],[25] in major reviews of *Anopheles* distribution and biology. The list was then further refined by anopheline experts from the Americas, Europe, Africa, Asia, and the Pacific, who co-author this article, to exclude 11 species that were not considered important vectors either because few recent data had implicated them in transmission or because they acted as vectors in only restricted geographical areas (Text S1). Following the convention of the major reviews in this area [23]–[25],[37],[38], the DVS of the *Anopheles* (*Cellia*) *gambiae* complex are listed separately. We hope also to map at species level three other complexes, where examination of the primary literature has indicated sufficient species-specific data (the *An.* (*Nyssorhynchus*) *albitarsis*, *An.* (*Cellia*) *culicifacies*, and *An.* (*Cellia*) *dirus* complexes). Further details are provided in the legend of the maps of each complex in Text S3 (for the *An.* (*Nyssorhynchus*) *albitarsis* complex) and Text S5 (for the *An.* (*Cellia*) *culicifacies* and *An.* (*Cellia*) *dirus* complexes).

## Comprehensive Literature Searches

An exhaustive and systematic search of formal and informal literature was conducted, mirroring the approaches developed by the MAP in building a global database of malaria parasite prevalence [39]. Only information collected after 31 December 1984 was searched. This criterion ensured that the data collected were representative of the contemporary distribution of the DVS and that the DVS occurrence records included only data collected using modern taxonomic species concepts [32],[33]. Following the introduction of cytological and then molecular methods to mosquito systematics, the taxonomy of the *Anopheles* changed radically, making many earlier species determinations potentially unreliable [32],[33],[40]–[43]. This date restriction also served to focus finite literature retrieval and abstracting resources on newer references, that are easier to retrieve from libraries, have sites that are less problematic to geo-position, and have authors that can often still be contacted with queries.

Records of the presence or absence of a DVS at a particular site and on a particular date were entered into the database so that information collected at different times from a locality was documented. Because abundance data have not been reported using methods that can be readily standardized across entomological surveys, only presence and absence data were used to generate the maps. Although the geographic distribution of the DVS in malaria-endemic countries is the first concern, data from any location was recorded because, as previously noted, information on DVS distribution is of major importance in those areas seeking to maintain their malaria-free status. Moreover, when modelling the fundamental niche of a species [44] using climate-envelope approaches [45], the aim is to be inclusive geographically, in an attempt to fully represent the environmental limits encompassed by its range.

Once a relevant literature source was identified, information was extracted using a list of data fields specified by a detailed pro forma (Text S2). Precise geo-positioning was conducted using established methods [39], so that any uncertainty associated with the positioning could be estimated [46]–[49]. Our strategy has been to first target the formally published literature and to use this base to direct further searches for informal (“grey”) literature sources and unpublished information held by relevant individuals and organisations. The results of this exercise were a total of 41,518 records with 22,249 spatially unique observations for all 41 DVS. These records are shown in full in a series of maps in Text S3, Text S4, and Text S5 for the American, Europe Africa, and Middle East and Asia Pacific region species, respectively. Short legends are included with each map indicating areas for which occurrence records are not well documented in the formal literature by comparison with digitised expert opinion distributions for each species. Informal searches are to be focussed on these areas of poor coverage and, where not prohibited by taxonomic identification issues, the inclusion date will be relaxed to the 31 December 1974. Ultimately, all these data will be made available in the public domain in accordance with the open access data sharing principles of the MAP [31].

## Collaborative Online Databases

Many initiatives are being developed to provide information on the geographical distribution of disease vectors, including the *Anopheles* (Table 1; for example surveys of the geographical distribution of different forms of insecticide resistance [50]–[52]). These initiatives will be a significant help in data acquisition. Duplication of search effort will be minimized by ensuring compatibility between different data abstraction ontologies (e.g., [53] and Text S2), so that where possible, data exchange can be automated. Where this cannot be achieved, data will be incorporated manually into the MAP archives with its provenance clearly recorded.

**Table 1:** Summary of the online resources for *Anopheles*.

Site	URL	Description
Anobase	http://www.anobase.org	Contains genomic/biological information for *An. gambiae s.l.*
Disease Vector Database	http://www.diseasevectors.org	Species occurrence data for 111 vectors including many *Anopheles* [10].
Lifemapper	http://www.lifemapper.org	Lifemapper correlates online geospatial species (plant, animal, and insect) occurrence data with a number of environmental variables to create distribution maps and total range predictions.
Malvecasia	http://www.itg.be/malvecasia	A multi-institutional European Union–funded project mapping insecticide resistance of *An. dirus*, *An. minimus*, *An. epiroticus*, and *An. vagus* in South-east Asia [52].
MARA	http://www.mara.org.za	The Mapping Malaria Risk in Africa site provides a spreadsheet documenting species occurrence data for *An. gambiae s.l.* [6].
MosquitoMap	http://www.mosquitomap.org	Interactive map showing global sampling points of mosquitoes from a number of genera, incorporating sampling details and full taxonomic descriptions [14].
VectorBase	http://www.vectorbase.org	Contains sequence and molecular vector-specific information for *An. gambiae s.l.*
WRBU	http://www.wrbu.org	The Walter Reed Biosystematics Unit has identification resources, images, and limited distribution maps for a range of medically important arthropods.

## New Species Mapping Techniques

Recent years have seen the development of a number of new techniques to predict species ranges [54]–[59], of which the most promising include methods based on boosted regression trees [60],[61], generalised additive models [62], and maximum entropy approaches [63]. In addition, Bayesian statistical approaches [64]–[66], which have been widely used in mapping malaria prevalence [67]–[72], have recently begun to be applied to mapping the relative frequency of *Anopheles* species [73]. Bayesian models are able to integrate information from disparate sources and allow the comprehensive quantification of prediction uncertainty, something that is often overlooked in species mapping exercises [74].

An important input into the iterative mapping process is expert advice from entomologists and public health workers with extensive experience of DVS in the field. To facilitate this input, the DVS have been split into three biogeographical regions: the Americas (nine species); Africa, Europe, and the Middle East (13 species); and the Asia-Pacific region (19 species) (Text S1). These experts have helped refine the expert opinion distributions digitised from the literature for the 41 DVS. These are presented alongside the species occurrence summaries in Text S3, Text S4, and Text S5.

## New Earth Observing Satellite Data

The statistical techniques we shall employ in future mapping efforts will model species occurrence as a function of environmental variables. We can then predict species distributions as a function of environmental conditions that can be obtained from Earth-observing satellite imagery [75]. During model formulation and validation we shall use coarse spatial resolution (∼8×8 km) multitemporal remotely sensed imagery [76] to reduce computational demand. Once the particular mapping technique is chosen, we will move to more contemporary Moderate Resolution Imaging Spectroradiometer (MODIS) satellite imagery, available globally at ∼1×1 km spatial resolution [77], to improve the spatial resolution of the predictions. Adapting temporal Fourier analyses techniques, which ordinate seasonal environmental data [78],[79], to cope with the irregular compositing periods of MODIS data, has been completed and the data has already been made available in the public domain [77].

## New Bionomics Review

The usefulness of the species range maps when available online [80], can be improved by combining them with summaries of the species-specific life history characteristics or “bionomics” of the DVS. Anopheline vector bionomics are critical in defining the appropriate (and inappropriate) modes of control at the national and local level [81]–[83]. For example, indoor residual spraying of houses for the control of a vector that is predominantly an outdoor resting species and prefers biting animals (e.g., *An.* (*Cellia*) *arabiensis*) is unlikely to be an optimal control strategy [84]. Conversely, if the vector feeds predominantly indoors and at night (e.g., *An.* (*Cellia*) *gambiae*), insecticide-treated nets are likely to be a very appropriate intervention [85],[86]. Information on characteristics of specific larval habitats and range will also be informative. Public health and education measures aimed at larval reduction may be feasible across large parts of the Middle East and Asia [87], where *An.* (*Cellia*) *stephensi* is the major DVS. This species readily breeds in urban areas, often using human-made water containers as its preferred larval habitat. Conversely, environmental management techniques such as installing tidal gates or constructing drainage systems are likely to be more effective as a permanent means of reducing or eliminating suitable coastal habitats of members of the *An.* (*Cellia*) *sundaicus* complex across substantial areas of South East Asia [88].

A systematic review of life-history characteristics pertinent to control is also timely as previous summaries become out of date [3],[89]–[97]. For example, as the taxonomy of the genus is better understood, it is evident that previous accounts which do not separate the different members of species complexes may omit or confuse critical biological information relevant for pest management. Examples of this occur in the *An. sundaicus* [98] and *An.* (*Cellia*) *minimus* complexes [99]. In addition, it would be desirable to incorporate the latest information on the phylogeny of the *Anopheles* [33], so that modern comparative methods [100] can be used to infer species characteristics from evolutionary relationships when no observations are available. This assembled information will be particularly useful for extending models of malaria transmission beyond *An. gambiae*, the species that has been the subject of most [101]–[103], but not all [104], attention. This will become increasingly important as operational and research communities alike continue to model the impact of vector control on malaria transmission [30].

Since abundance cannot be modelled with these opportunistic data assemblies, the bionomics review will also facilitate a ranking of the importance in malaria transmission of the different DVS in each region. This ranking will enable multiple species maps to be overlaid to obtain a more accurate picture of the overall epidemiological significance of the local DVS community and thus provide a better understanding of the complexity of transmission in an area. It is clear that subregional ecological diversity, coupled with the behavioural plasticity of many DVS, will require that any maps, and associated bionomics information provided, be interpreted and acted on cautiously with local expert knowledge.

## Conclusions

The completed DVS databases and predictive maps will be made available online once generated, alongside the wider portfolio of MAP products, including spatial limits and endemicity maps for the human malaria parasites [1],[2]. This juxtaposition of information should represent an important cartographic resource for those engaged in malaria control and where feasible, its elimination. The success and long-term sustainability of this DVS mapping initiative depends critically on its continued support, development, and refinement in the malaria vector control and research communities. We hope that the information on the aims and objectives provided here, and the commitment to providing data in an open access venue, will help ensure that support.

## Supporting Information

Text S1 Defining the dominant *Anopheles* vector species (and species complexes) of human malaria.
(0.14 MB DOC)

Text S2 Pro forma for the abstraction of occurrence data for the dominant *Anopheles* vectors of human malaria.
(0.08 MB DOC)

Text S3 Maps of expert opinion distribution and species occurrence records for the dominant *Anopheles* vector species (and species complexes) of human malaria in the Americas.
(1.82 MB PDF)

Text S4 Maps of expert opinion distribution and species occurrence records for the dominant *Anopheles* vector species (and species complexes) of human malaria in Africa, Europe, and the Middle East.
(3.02 MB PDF)

Text S5 Maps of expert opinion distribution and species occurrence records for the dominant *Anopheles* vector species (and species complexes) of human malaria in the Asia-Pacific region.
(4.11 MB PDF)

## Abbreviations

DVS: dominant vector species
MAP: Malaria Atlas Project
MODIS: Moderate Resolution Imaging Spectroradiometer
