# Supplementary material for: Developing Global Maps of the Dominant Anopheles Vectors of Human Malaria
Source: PLoS Med. 2010 Feb 9;7(2):e1000209. doi: 10.1371/journal.pmed.1000209 (PMC2817710; doi:10.1371/journal.pmed.1000209)
Supplement: Text S1 — Defining the dominant Anopheles vector species (and species complexes) of human malaria. (0.14 MB DOC) [file pmed.1000209.s001.doc]

**Text S1**. Defining the dominant *Anopheles* vector species (and species complexes) of human malaria.

| **Anopheline species or species complex** | **White [1]** | **Service [2,3]** | **Kiszewski [4]** | **Mouchet [5]** | **Exc.** | **Inc.** | **Region** | **TAG final** |
| --- | --- | --- | --- | --- | --- | --- | --- | --- |
| *aconitus* Dönitz, 1902: *An.* (*Cellia*) | 10 | 10 |  | 10 |  | 1 | AP | 1 |
| *albimanus* Wiedemann, 1820: *An.* (*Nyssorhynchus*) | 2, 3 | 2, 3 | 2, 3 | 2, 3 | 1 | 1 | AM | 1 |
| *albitarsis**: *An.* (*Nyssorhynchus*) | 3 | 3 |  |  |  | 1 | AM | 1 |
| *annularis*† van der Wulp, 1884: *An.* (*Cellia*) |  |  | 8, 9 |  |  | 1 | AP | 1 |
| *aquasalis* Curry, 1932: *An.* (*Nyssorhynchus*) | 2, 3 | 2, 3 | 2, 3 | 2, 3 | 1 | 1 | AM | 1 |
| *arabiensis* Patton, 1905: *An.* (*Cellia*) | 7 | 7 | 7 | 7 | 1 | 1 | AEME | 1 |
| *argyritarsis* Robineau-Desvoidy, 1827: *An.* (*Nyssorhynchus*) |  | 2 |  |  |  | 1 | AM |  |
| *atroparvus* van Thiel, 1927: *An.* (*Anopheles*) | 4, 5 | 4, 5 | 4, 5 | 4, 5 | 1 | 1 | AEME | 1 |
| *balabacensis* Baisas, 1936: *An.* (*Cellia*) | 10 | 10, 11 |  | 10 |  | 1 | AP | 1 |
| *barbirostris**: *An.* (*Anopheles*) |  |  | 9, 10 |  |  | 1 | AP | 1 |
| *bellator* Dyar & Knab, 1906: *An.* (*Kerteszia*) | 3 |  |  |  |  | 1 | AM |  |
| *campestris*† Reid, 1962: *An.* (*Anopheles*) | 10 | 10 |  |  |  | 1 | AP |  |
| *cruzii* Dyar & Knab, 1908: *An.* (*Kerteszia*) | 3 |  |  |  |  | 1 | AM |  |
| *culicifacies**: *An.* (*Cellia*) | 8 | 8 | 8 | 8 | 1 | 1 | AP | 1 |
| *darlingi* Root, 1926: *An.* (*Nyssorhynchus*) | 2, 3 | 2, 3 | 2, 3 | 2, 3 | 1 | 1 | AM | 1 |
| *dirus**: *An.* (*Cellia*) | 9, 10 | 9, 10 | 9, 10 | 9, 10 | 1 | 1 | AP | 1 |
| *donaldi* Reid, 1962: *An.* (*Anopheles*) | 10 | 10 |  |  |  | 1 | AP |  |
| *farauti**: *An.* (*Cellia*) | 12 | 12 |  | 12 |  | 1 | AP | 1 |
| *flavirostris* (Ludlow, 1914): *An.* (*Cellia*) |  | 10 | 10 | 10 |  | 1 | AP | 1 |
| *fluviatilis**: *An.* (*Cellia*) | 8, 9 | 8, 9 | 8, 9 | 8, 9 | 1 | 1 | AP | 1 |
| *freeborni* Aitken, 1939: *An.* (*Anopheles*) | 1 | 1 | 1 |  |  | 1 | AM | 1 |
| *funestus* Giles, 1900: *An.* (*Cellia*) | 7 | 7 | 7 | 7 | 1 | 1 | AEME | 1 |
| *gambiae* Giles, 1902: *An.* (*Cellia*) | 7 | 7 | 7 | 7 | 1 | 1 | AEME | 1 |
| *koliensis* Owen, 1945: *An.* (*Cellia*) |  | 12 |  | 12 |  | 1 | AP | 1 |
| *labranchiae* Falleroni, 1926: *An.* (*Anopheles*) | 5 | 5 | 5 | 5 | 1 | 1 | AEME | 1 |
| *lesteri* Baisas & Hu, 1936: *An.* (*Anopheles*) |  | 11 | 11 | 11 |  | 1 | AP | 1 |
| *letifer* Sandosham, 1944: *An.* (*Anopheles*) | 10 | 10 |  |  |  | 1 | AP |  |
| *leucosphyrus**: *An.* (*Cellia*) | 10 | 10 |  |  |  | 1 | AP | 1 |
| *ludlowae* (Theobald, 1903): *An.* (*Cellia*) | 10 | 10 |  |  |  | 1 | AP |  |
| *maculatus* Group: *An.* (*Cellia*) | 10 | 10 | 10 | 10 | 1 | 1 | AP | 1 |
| *marajoara* Galvão & Damasceno, 1942: *An.* (*Nyssorhynchus*) |  |  | 2,3 |  |  | 1 | AM | 1 |
| *melas* Theobald, 1903: *An.* (*Cellia*) | 7 |  | 7 |  |  | 1 | AEME | 1 |
| *merus* Dönitz, 1902: *An.* (*Cellia*) |  |  | 7 |  |  | 1 | AEME | 1 |
| *messeae* Falleroni, 1926: *An.* (*Anopheles*) |  |  | 4, 5 |  |  | 1 | AEME | 1 |
| *minimus**: *An.* (*Cellia*) | 9, 10 | 9, 10 | 9, 10 | 9, 10 | 1 | 1 | AP | 1 |
| *moucheti* Evans, 1925: *An.* (*Cellia*) |  |  |  | 7 |  | 1 | AEME | 1 |
| *multicolor* Cambouliu, 1902: *An.* (*Cellia*) |  |  | 6 |  |  | 1 | AEME |  |
| *nigerrimus* Giles, 1900: *An.* (*Anopheles*) | 10 | 10 |  |  |  | 1 | AP |  |
| *nili**: *An.* (*Cellia*) |  |  |  | 7 |  | 1 | AEME | 1 |
| *nuneztovari**: *An.* (*Nyssorhynchus*) | 3 | 3 | 3 | 3 | 1 | 1 | AM | 1 |
| *pharoensis*† Theobald, 1901: *An.* (*Cellia*) | 6 | 6 | 6 |  |  | 1 | AEME |  |
| *pseudopunctipennis**: *An.* (*Anopheles*) | 3 | 3 | 3 | 3 | 1 | 1 | AM | 1 |
| *pulcherrimus* Theobald, 1902: *An.* (*Cellia*) |  |  | 8 |  |  | 1 | AEME |  |
| *punctimacula* Dyar & Knab, 1906: *An.* (*Anopheles*) | 3 | 3 |  |  |  | 1 | AM |  |
| *punctulatus**: *An.* (*Cellia*) |  | 12 | 12 | 12 |  | 1 | AP | 1 |
| *quadrimaculatus* Say, 1824: *An.* (*Anopheles*) | 1 | 1 | 1 | 1 | 1 | 1 | AM | 1 |
| *sacharovi* Favre, 1903: *An.* (*Anopheles*) | 5 | 5 | 5 | 5 | 1 | 1 | AEME | 1 |
| *sergentii* (Theobald, 1907): *An.* (*Cellia*) | 6 | 6 | 6 | 6 | 1 | 1 | AEME | 1 |
| *sinensis**: *An.* (*Anopheles*) | 4, 11 | 11 | 4, 11 | 4, 11 | 1 | 1 | AP | 1 |
| *stephensi* Liston, 1901: *An.* (*Cellia*) |  |  | 8 | 8 |  | 1 | AP | 1 |
| *subpictus**: *An.* (*Cellia*) | 10 | 10, 12 |  |  |  | 1 | AP | 1 |
| *sundaicus**: *An.* (*Cellia*) | 10 | 10 | 10 | 10 | 1 | 1 | AP | 1 |
| *superpictus* Grassi, 1899: *An.* (*Cellia*) | 5 | 5 | 5 | 5 | 1 | 1 | AEME | 1 |
| Totals | 38 | 40 | 35 | 31 | 21 | 53 | 53 | 41 |

The full scientific name (genus, subgenus and species) of the anopheline species is given along with the author(s) who first described the species, including the year of publication of their description. The brackets encompassing author-date combinations indicate if the genus name has changed since the species was first described. The * denotes that a “species” is now recognized as a species complex and the † denotes that a “species” is now suspected to be a species complex. The *An.* (*Cellia*) *gambiae* complex has been separated into constituent species following the convention in the main reviews consulted (see below). The next four columns show if White [1], Service [2,3], Kiszewski [4] or Mouchet [5] designated the “nominal” species as a principal, main, dominant or principaux vector of human malaria, respectively. The numbers in each of these columns record in which Macdonald malaria epidemiology zone [6] the species can be found: 1 - North American; 2 - Central American; 3 - South American; 4 - North Eurasian; 5 - Mediterranean; 6 - Afro-Arabian; 7 - Afrotropical (formerly Ethiopian); 8 - Indo-Iranian; 9 - Indo-Chinese hills; 10 - Malaysian; 11 - Chinese and 12 - Australasian. The exclusive (Exc.) column counts those species identified in all four reviews. The inclusive (Inc.) column counts those species identified by any of the four authors. Totals are given at the bottom of the table. The candidate dominant vector species (DVS) considered for mapping are the inclusive set minus *An.* (*Nyssorhynchus*) *argyritarsis* which has since been shown to have been incorrectly incriminated as a vector of human malaria [7]. The DVS groupings are shown in the region column: the American (AM) group (12/52), the Africa, Europe and the Middle East (AEME) group (16/52) and the Asia Pacific (AP) group (24/52). The final DVS list (shaded rows) was refined during two meetings of a technical advisory group (reflected in the authorship) by excluding 11 vectors which had a restricted range and little contemporary evidence to incriminate them as vectors of malaria; leaving 9/41 in the AM group, 13/41 in the AEME group and 19/41 in the AP group.

**References**

1. White GB (1989) Malaria. Geographical distribution of arthropod-borne diseases and their principal vectors WHO/VBC/89967. Geneva: World Health Organization, Division of Vector Biology and Control. pp. 7-22.

2. Service MW (1993) The *Anopheles* vector. In: Gilles HM, Warrell DA, editors. Bruce-Chwatt's Essential Malariology. Third edition ed. London: Edward Arnold. pp. 96-123.

3. Service MW (1993) Appendix II. Characteristics of some major *Anopheles* vectors of human malaria. In: Gilles HM, Warrell DA, editors. Bruce-Chwatt's Essential Malariology. Third edition ed. London: Edward Arnold. pp. 305-310.

4. Kiszewski A, Mellinger A, Spielman A, Malaney P, Sachs SE, et al. (2004) A global index representing the stability of malaria transmission. Am J Trop Med Hyg 70: 486-498.

5. Mouchet J, Carnevale P, Coosemans M, Julvez J, Manguin S, et al. (2004) Biodiversité du paludisme dans le monde. Montrouge, France: John Libbey Eurotext. 428 p.

6. Macdonald G (1957) Local features of malaria. The epidemiology and control of malaria. London: Oxford University Press. pp. 63-99.

7. Rubio-Palis Y (1993) Is *Anopheles argyritarsis* a vector of malaria in the neotropical region? J Am Mosq Control Assoc 9: 470-471.
