# Supplementary material for: Developing Global Maps of the Dominant Anopheles Vectors of Human Malaria
Source: PLoS Med. 2010 Feb 9;7(2):e1000209. doi: 10.1371/journal.pmed.1000209 (PMC2817710; doi:10.1371/journal.pmed.1000209)
Supplement: Text S2 — Pro forma for the abstraction of occurrence data for the dominant Anopheles vectors of human malaria. (0.08 MB DOC) [file pmed.1000209.s002.doc]

**Text S2**. Pro forma for the abstraction of occurrence data for the dominant *Anopheles* vectors of human malaria.

**Overall objective**

The Malaria Atlas Project (MAP, http://www.map.ox.ac.uk) aims to search and archive information on the global distribution of the dominant *Anopheles* vector species of human malaria. These data will provide valuable information on the contemporary range of these *Anopheles* species and help guide the application of appropriate vector control.

The inclusion criteria and data abstraction procedures are outlined in this data entry pro forma. These notes should be read prior to starting the data abstraction process, and again, after a week or two into the data entry trial period. Past experience shows this will answer most of the common queries that arise.

**Literature source**

A. ENL_ID. Enter the unique record of the PDF source examined.

B. INITIALS. Enter the initials of the first author. Where there is more than one initial, separate them with a space, *e.g.* S I

C. AUTHOR. Enter the surname of the first author of the publication only.

D. YEAR. Enter the year of publication.

E. REPORT_TYPE. Enter a category from the drop-down menu. The options are journal, unpublished work, report, MoH (Ministry of Health) report, thesis, conference abstract, other.

F. PUBLISHED. Enter from the drop-down menu “yes” if it is published in the formal literature (*i.e.* by a commercial publisher and likely to have copyright restrictions) and “no” if it is in the “grey” literature (*e.g.* a doctoral thesis).

G. VDATA. Enter from the drop-down menu “yes” if it contains vector data and “no” if it does not.

Scan the article before reading in detail, concentrating particularly on the methods and results. Reading the abstract and searching for “*Anopheles*”, “*An*”, “mosquito” and “vector” with the Adobe search facility can help identify relevant data. Be careful with this scan function, however, as some PDFs do not allow searches; it should augment and NOT replace your thorough overview of the paper. If there are no relevant data, enter “no”, as instructed above and leave the rest of the fields in the row blank and move onto the next reference in the sequence. The exception is to use the NOTES_VECTOR column (column BO) to enter: (i) if the citation indicates another source for which post 31 December 1984 entomological data may exist and (ii) if the citation has an explicit link to another survey (*e.g.* where entomology work was done concurrently to the study described in the PDF, but reported elsewhere).

**Geographical scope of surveys to include and geo-positioning information**

Records of presence from any location should be incorporated. Although we are primarily concerned with the distribution of the main/dominant vectors in malaria endemic countries, their ranges will extend beyond these national boundaries and when attempting to accurately model the environmental limits of a species, we must be geographically inclusive to faithfully reflect its fundamental niche.

You will be provided with three accompanying excel spreadsheets: “00 PR geopositions.xls” which lists all the sites previously geo-positioned during the parasite prevalence data entry; “01 admin2_ID.xls” which lists identification codes for most administration level 2 locations in malaria endemic countries (see below) and “01 admin2_ID – non MEC.xls” which lists administration level 2 codes for non- malaria endemic countries (see below). Geo-positioning should follow the shaded boxed guidelines below. If there is more than one site included in the sample survey, add a new row for each location.

H. SITE_ID. First check that the site has not already been geo-positioned in “00 PR geopositions.xls”. If it has, check and confirm that the information given in “00 PR geopositions.xls” is correct for the site in question and note how you have done this in the SITE_NOTES. The sites listed in the “00 PR geopositions.xls” have corresponding area type information and “guess” status, which should also be an accurate representation of the “new” site. If either the guess status or the area type differ, the SITE_ID should not be used, and the full geo-positioning must be given. If the “new” site information corresponds to the site listed in “00 PR geopositions.xls”, copy and paste the SITE_ID number into the SITE_ID column of your datasheet and skip to the GOOD_GUESS column. Where the SITE_ID cannot be used, write the SITE_ID number in the SITE_NOTES and a short explanation of why. If the country is listed but the site is not, write “not listed” in the SITE_NOTES as this shows it has not been overlooked. The “00 PR geopositions.xls” only lists malaria endemic countries so if the country is not listed, write “N/E” (non-endemic) in the SITE_NOTES.

I. COUNTRY. Select the country name where the mosquito sampling occurred from the drop-down menu.

J. FULL_NAME. Enter the name of the site where the mosquito sampling occurred exactly as it is given in the paper. List any alternative spellings which you come across in the SITE_NOTES.

K. ADMIN1_PAPER. Enter admin level 1 if given in the paper and write “not specified” if not. Admin levels should not be inferred from any other sources. Admin level 1 is the first country sub-division, *e.g.* state in the U.S.A. or province in Kenya.

L. ADMIN2_PAPER. Enter admin level 2 if given in the paper and write “not specified” if not. Admin level 2 is the next sub-division, *e.g.* county in the U.S.A. or district in Kenya.

M. ADMIN3_PAPER. Enter admin level 3 if given in the paper, leave field blank if not. Note the Alexandria geo-referencing database (http://clients.alexandria.ucsb.edu/globetrotter/) and Wikipedia are useful tools to help identify the administrative level of an area.

N. ADMIN2_ID. Copy and paste the unique admin2 code from the “01 admin2_ID” excel spreadsheet provided. An ADMIN2_ID should always be given (see point 8 under the MAP geo-location guidelines below). Only one ID number should be entered. If a sampling site, for example a polygon, crosses two ADMIN2 areas, use Encarta to establish which admin area has the greater proportion of the site within it, and use this ID number. Make a note in the SITE_NOTES of what you have done, including the other admin area name and ID. Many non-malaria endemic countries are not included in the “01 admin2_ID.xls” sheet, for example: USA, Australia, New Zealand. For these countries select the corresponding ID from the “01 admin2_ID – non MEC.xls”

O. LAT. Enter the latitude of the mosquito sample location in decimal degrees.

P. LONG. Enter the longitude of the mosquito sample location in decimal degrees. There are seven additional sets of LAT LONG columns (Q to AD) included in the datasheet in case of multi-point records or to clarify wide area/polygon boundaries (see point 9 in the MAP geo-location guidelines below). If the site cannot be located, leave these cells blank. LAT LONG coordinates must be specific to the site listed in SITE_NAME, no substitute coordinates should be entered. Enter detailed information explaining how the coordinates were found and checked in the SITE_NOTES.

AE. LATLONG_SOURCE. Select the geo-positioning source from the drop-down menu. The options are: the original paper (“paper”), personal communication (“pers.comm”), Microsoft Encarta (“encarta”), Geonames (“geoname”), any other web database (“webdb”), any other paper map (“map”), a combination of techniques (“combination”) or others (“other”). If the site is not listed in Encarta, but you have used Encarta to estimate the coordinates using information from the article, this is “combination”. If coordinates are given in the paper and the site is also in Encarta, the LATLONG_SOURCE will be Encarta. Specify in the SITE_NOTES that the paper gives the same coordinates as Encarta.

AF. GOOD_GUESS. Enter “yes” or “no” from the drop-down menu. A location is entered as a good guess when it is not listed in any of the geo-location sources but ancillary information from the article provides a good guide to locate it with some degree of accuracy (*e.g.* article states that the site is 4 km north on the road between town x and town y; this can be easily located in Encarta using the ruler tool). A rule of thumb is: using the information from the article, if you are confident that the estimated lat/longs fall within a 5km radius of the true site location, this is a good guess.

AG. BAD_GUESS. Enter “yes” or “no” from the drop-down menu. In contrast, a bad guess is entered when the location is not listed in any of the geo-location sources and the ancillary information from the article is more vague (*e.g.* article states that the site is near town x towards the north but no distance is specified). A rule of thumb is: using the information from the article, if you are confident that the estimated lat longs fall outside a 5 km radius of the true site location, this is a bad guess.

AH. SITE_NOTES. Note clearly any decisions you made while geo-referencing that will help a third person understand and enable them to repeat the choices you have made. Also, if the LATLONG_SOURCE used is not listed in the drop-down menu (see above) specify your source here after selecting “other” or name the web database used when selecting “webdb”, *e.g.* Alexandria.

AI. AREA_TYPE. Enter the estimated size of the sampling area. It is not often detailed in the source and may need to be inferred from information in Encarta or other sources. The options are: “point” (≤ 10 km^2^), “wide-area” (>10 and ≤ 25 km^2^), “small polygon” (>25 and ≤ 100 km^2^) and “large polygon” (>100 km^2^). The area type must accurately represent the site. For example if the study was done in a village (a “point”) that cannot be geo-located, all the information in the data line must still accurately represent this site, but with the lat/longs left blank. Do not enter information for a polygon that will encompass the site as, although this means the site is included in some form, precision is lost. We will contact authors at a later date to find the specific site coordinates for missing information.

AJ. RURAL-URBAN. Select either “urban”, “rural” or “”n.a.” from the drop-down menu. The rural/urban definitions are very subjective so unless the article explicitly defines a site as rural/urban, enter “n.a.” in this field.

AK. FOREST. Select “yes”, “no” or “n.a.” from the drop-down menu, again this information, similarly for point AL below, should not be inferred and should only be entered if stated in the article.

AL. RICE. Select “yes”, “no” or “n.a.” from the drop-down menu. The ecological descriptors are not mutually exclusive, for example, a site can be rural and have areas of forest and rice fields.

**MAP geo-location guidelines**

The following are steps we have adopted to geo-position our points:

****Encarta is the standard tool with which we either locate or confirm geographic coordinates****

****Check and double check all site coordinates in Encarta****

****Remember to pay particular attention to the sign in front of all decimal degree coordinates and check that the latitude and longitude are typed in the correct cell. These are the two most common mistakes. In decimal degree format, North and East are positive and South and West are negative****

****We aim to record at least two decimal places of precision in decimal degrees, but preferably four****

1) Check the source pdf for a description of the site/area/community to be geo-positioned. This information is essential because often there are duplicate site names within a region and the correct choice of geo-position is guided by this ancillary information. Examples are “the community is 14 km southeast from the capital” or “by the delta of the Shebelle river”. Note down all the information provided in the SITE_NOTES using as much detail as possible. The source pdf may also include a map of the area. Such maps are often the most important means of positioning validation. The source pdf may also record some general coordinate information. For instance, it may describe a group of villages situated between 6 N, 40 E and 5.9 N, 40.5 E. Keep a record of this information as it is also a useful verification tool.

2) Use Microsoft Encarta Premium Edition 2007 as the gold standard for geo-positioning. When searching for a site in Encarta, the location listed NEEDS to be consistent with the other geographical information recorded from the source (see point 1) as there are often duplicate site names. If you are unsure you have the correct site, leave the coordinates blank and give a full explanation why in the SITE_NOTES. If a map is available in the source pdf, the positioning of the lat/longs in Encarta should resemble this map, as closely as possible.

3) Encarta will often either list many close matches for the record or no close match at all. In such cases use alternative online resources (see point 6) and the ancillary information recorded (see point 1). Using the example above we would locate the capital city and investigate what is found approximately 14 km to the southeast of it. Here you may find the site listed with an alternative spelling (different spellings may not be indexed by Encarta as the search keyword), which, after some investigation, is obviously a match. Make sure to note the full process of checks you have made to confirm this is the correct site, and any alternative spellings, in the SITE_NOTES.

4) Authors often give the co-ordinates of the survey location. However, lat/longs cited in a paper may use a different projection/datum and hence would appear in a slightly different location in Encarta. Encarta uses a global lat/long projection that we need for our mapping so always use coordinates from Encarta in preference. If you are completely sure that the point in the paper is different from the one shown in Encarta then use the information given in the paper. Double check the coordinates in Encarta (*i.e.* place the cursor on the map where the “dynamic sensor” reads the lat/longs stated) and check if this location complies with the description or map in the article (see point 1); there have been cases in which the lat/longs given by the authors are not very precise and a more accurate option is found in Encarta by using this approach.

5) Online thesauruses such as the GeoNet Names Server, Alexandria, GoogleEarth and Fallingrain, among others, can be useful for sites not found in Encarta. Wikipedia and Maplandia can also be useful, particularly to identify admin divisions within a country. Again, always map the lat/longs in Encarta to double check ancillary information and see if the location resembles the description of the source.

http://clients.alexandria.ucsb.edu/globetrotter/

http://www.fallingrain.com/world/

http://gnswww.nga.mil/geonames/GNS/index.jsp

http://www.getty.edu/research/conducting_research/vocabularies/tgn/

http://www.maplandia.com/

6) Our last resort is to lookup place names in Google (www.google.com). This is a rather powerful strategy for difficult places. From experience the best keyword combination is: *“name of place” + “name of country” + “name of administrative unit” +* the word *“map”*. If lucky we find the coordinates in a document published on the web or on other web resources. Useful information can also be found in maps contained in other papers, documents, reports, travel guides, *etc.* Remember to always cross-check any information found from other sources in Encarta and to list in the SITE_NOTES any details which will help anyone checking the geo-positioning.

7) It is not uncommon to have to make an informed guess about the geo-positioning. To allow for the uncertainty introduced, we indicate whether best guess decisions are either “good” or “bad”. These decisions have varying degrees of uncertainty, but as a rule of thumb, any site for which there is no unambiguous match in any source (*i.e.* no specific lat/longs were found for the specific name) a “good” guess reflects where the lat/longs chosen are very likely to correspond (within 5 km) to the site in question (*e.g.* village 10 km North East of Brazzaville). In a “bad” guess the lat/longs are likely to be in the correct broad area (not within 5 km) but unlikely to be in the right place (*e.g.* village near Brazzaville, to the North East of the city). Give a full description in the SITE_NOTES of how and why the site has been categorised as a “good” or “bad” guess.

8) The ADMIN2_ID field SHOULD ALWAYS contain a number pasted from the relevant Excel file. This number is picked in a progressive manner from the list of administrative units. First “Auto-filter” by country, then by admin1, and from the options remaining, select the admin2 that matches the information given in the article and copy/paste the ADMIN2_ID number into the ADMIN2_ID column in your datasheet. If no match is found, pick “not found” from the list, and if no admin2 is specified in the paper, then select “not specified”. If an admin1 cannot be found on the list for the first filtering but you still have an admin2 to look for, then filter by country only and look among all listed admin2s for that country. If, however, neither admin1 nor admin2 are specified, select the admin id number that corresponds to “Not specified” for both admin1 and admin2. Some of the ADMIN2_ID sites have ID numbers listed from several sources. The order in which to use the ID numbers is: GAUL, SALB, FAO, then any others.

9) Wide-areas or polygons should give the smallest area possible - try to avoid large polygons. For example, if an article presents amalgamated data from several village sites within a district and further investigation shows the villages to be grouped in a small area within this district, keep the spatial resolution as high as possible by geo-positioning all the villages, or as many as can be located rather than entering the district as a large polygon. Measure the distance between the most distant villages using the ruler tool in Encarta and use this information to allocate the correct area type. [point (≤ 10 km^2^), wide-area (>10 and ≤ 25 km^2^), small polygon (>25 and ≤100 km^2^) and large polygon (>100 km^2^)]. The villages’ coordinates should be entered into the additional LAT LONG columns in the datasheet to quantify the polygon/wide-area boundaries. Up to eight sets of coordinates can be entered. Make a note of what has been done and of any SITE_ID numbers for the villages if they are available. If you have no further information regarding the polygon, just a district name, and cannot make a more detailed polygon, then one set of coordinates for the centroid of the district should be used – again, make clear notes of how the coordinates were sourced and placed.

**Time of survey**

All surveys conducted before 01 January 1985 are excluded, with the exception of any studies that begin prior to 1985 but carry through into and/or post 1985. We have constrained the sample to consider only the last 25 years since these are more representative of the contemporary distribution of *Anopheles* vectors and include the most recent taxonomic classifications and more modern techniques for accurate identification.

AM. MONTH_STVEC. Enter the month the mosquito sampling started from the drop-down menu.

AN. MONTH_ENVEC. Enter the month the mosquito sampling ended from the drop-down menu.

AO. YEAR_STVEC. Enter the year the mosquito sampling started from the drop-down menu.

AP. YEAR_ENVEC. Enter the year the mosquito sampling ended from the drop-down menu.

The strategy for dealing with multiple and longitudinal surveys is involved. The goal to remember when making data abstracting decisions is to record as much information as possible without making any assumptions. If authors report a monthly longitudinal survey over three years and only present summary data over the whole survey, this would be recorded as one survey of 36 months’ duration. If for the same survey, however, they reported the data by month this would be recorded as 36 surveys, each of a month’s duration. This means that we will also retain seasonality information for future studies. As such it is useful to include the months where a particular species, present in previous months, is no longer present. For example if a study gives data showing that 25 *An. darlingi* individuals were collected on human bait in March but none were found at that site in April, include this data line but with “0” in the “N” column next to the “Man-biting (MB)” sampling technique. If surveys are conducted before and after control regimes are introduced, these should be entered as separate surveys and the control study information indicated in the CONTROL and CONTROL_TYPE columns.

**Species type**

AQ. SPECIES1. Select one of 41 main/dominant vector species or species complexes from the drop-down menu.

AR. *s.s.*/*s.l.* Specify if the authors designate *sensu lato* (in the general sense) or *sensu stricto* (in the strict sense). If not specified enter “n.a.”

AS. ASSI. Select “yes” from the drop-down menu if there is any additional species specific information (ASSI) given. This could be the mention of different chromosomal forms, genotypes, sub-species, molecular types *etc*. that designate some kind of sub-species complex variation. At present we only have a specified sub-species complex column for *An. gambiae* (see below). For this species complex there is often further information (e.g. “*An. gambiae* *s.l.* was identified morphologically from the collection, then using PCR the 2 siblings *An. arabiensis* and *An. gambiae* *s.s.* were identified. Of the *An. gambiae* *s.s.* Savannah and Mopti forms were found”). The Savannah and Mopti information would then be given in the ASSI notes (see below for more information about *An. gambiae* *s.l.* and ASSI).

AT. NOTES_ASSI. Document the type of species specific information available.

AU. SPECIES2. Select one of six vector species from the drop-down menu from the *An. gambiae* complex if they have been specifically identified in the current study, otherwise enter “n.a.”

If there is more than one species collected include a new row for each new species. The ENL_ID field is identical for each new row. The 41 *Anopheles* species and species complexes that are listed in SPECIES1 are: *An. aconitus*, *An. albimanus*, *An. albitarsis*, *An. anthropophagus* (*An. anthropophagus* has recently been shown to be a synonym for *An lesteri* – mention this in the vector notes when either of these species names are listed), *An annularis, An. aquasalis*, *An. arabiensis*, *An. atroparvus*, *An. balabacensis*, *An. barbirostris*, *An. culicifacies*, *An. darlingi*, *An. dirus*, *An. farauti*, *An. flavirostris*, *An. fluviatilis*, *An. freeborni*, *An. funestus*, *An. gambiae*, *An. koliensis*, *An. labranchiae*, *An. leucosphyrus*, *An. maculatus*, *An. marajoara*, *An. melas*, *An. merus*, *An. messeae*, *An. minimus, An. moucheti, An. nili*, *An. nuneztovari*, *An. pseudopunctipennis*, *An. punctulatus*, *An. quadrimaculatus*, *An. sacharovi*, *An. sergentii*, *An. sinensis*, *An. stephensi*, *An. subpictus*, *An. sundaicus* and *An. superpictus*.

***Anopheles gambiae* complex**

The unique importance of the *An. gambiae* complex, in containing the world’s most efficient vectors of human malaria, requires that we collate as much epidemiologically relevant taxonomic information as is documented for the species complex. We may consider extending this level of detail to other species complexes and will provide further instructions if necessary.

If no sibling information is given for the complex simply enter *An. gambiae* and *s.l.* as instructed above in SPECIES1. If further identification has been done, for example, the study states that “*An. gambiae* *s.l.* were identified using morphological characteristics and then, using PCR, the samples were further identified”; enter the sibling species’ name (*An. gambiae* *s.s.*, *An. arabiensis, An. quadriannulatus,* *An. quadriannulatus* species B*, An. bwambae,* *An. melas* or *An. merus*) in the SPECIES2 column. In the case of *Anopheles gambiae s.s.* please also note chromosomal form (Forest, Bissau, Bamako, Savanna and Mopti) or molecular form (M/S) if this has been recorded, in the NOTES_ASSI field. If the article simply states the presence of one of the species within the *An*. *gambiae* complex, without first referring to *An. gambiae* *s.l.*, enter the given species into the SPECIES1 column (where listed) and n.a. in the SPECIES2 column.

**ASSI**

*Anopheles gambiae* is not the only anopheline species that has additional specific species information (ASSI) that we need to record. Where a species has split into two or more closely related sibling species it is very difficult to distinguish between them using morphological characteristics (this is a main definition of sibling species). These sibling species can only be distinguished using molecular techniques, for example using RAPD primers (a DNA probing technique).

The term “subspecies” tends to refer to populations of a species that have been geographically separated for such a period of time as to have evolved genetic differences yet they are still able to reproduce with the original source species (like a race or plant variety). These can often be distinguished using morphological characteristics; however, there can always be a level of subjectivity with morphological identification so molecular techniques are often used to help distinguish subspecies.

EXAMPLE: A species is described in an article as *sensu lato*, such as *An. minimus* complex for instance, and the authors state that they have further identified the species (just as they would with *gambiae* complex) using RAPD primers to find they are all of the subspecies *An. harrisoni* (previously *An.* *minimus* sp C)*.* You would choose “yes” in the ASSI, and write the subspecies name into the ASSI_notes. You would do the same if the authors say they further identified a species and found, for example, all to be of the molecular form A (a sibling species or type).

**Sampling / collection / trapping method**

AV. MOSSAMP_TECH1. Select the first mosquito sampling method listed in the article from the drop-down menu.

AW.N1. Enter the total number collected from the first mosquito sampling method where specified. If not specified highlight yellow.

AX. MOSSAMP_TECH2. Select the second mosquito sampling method listed in the article from the drop-down menu.

AY. N2. Enter the total number collected from the second mosquito sampling method where specified. If not specified highlight yellow.

AZ. MOSSAMP_TECH3. Select the third mosquito sampling method listed in the article from the drop-down menu.

BA. N3. Enter the total number collected from the third mosquito sampling method where specified. If not specified highlight yellow.

BB. MOSSAMP_TECH4. Select the fourth mosquito sampling method listed in the article from the drop-down menu.

BC. N4. Enter the total number collected from the fourth mosquito sampling method where specified. If not specified highlight yellow.

BD. ALLN. Enter the sum of the numbers in columns N1, N2 etc to show the total collected from all mosquito sampling methods.

BE. ALLNCHECK. This field automatically checks if the paper totals tally. Check if not zero but leave if a non-zero value reflects a genuine mistake in the original paper.

The following mosquito sampling techniques can be selected: animal biting (“AB”), animal shed resting (“ARD”), animal baited net trap (“ABN”), animal biting outdoors (“ABO”), carbon dioxide baited net trap (“CO2”), house resting inside (“HRI”), human baited net trap (“HBN”), indoor light trap (“ILT”), larval collection (“L”), light trap (“LT”), man biting indoors (“MBI”), man biting outdoors (“MBO”), outdoor light trap (“OLT”), outlet window trap (“OWT”), outside shelter (“OS”), outside shelter artificial (“OSA”), outside shelter natural (“OSN”), resting outdoors (“RO”) or unknown (“?”). If the sampling method used in the study is not one of the methods listed or if it is unclear, enter “?” (unknown) in MOSSAMP_TECH1 and note it in the NOTES_VECTOR column. If it is not mentioned at all, enter “n.a.” (not answered). If more than four sampling techniques were used, record the four predominant ones in the relevant columns and any additional ones in the NOTES_VECTOR column.

**Identification technique(s)**

BF. MOS_ID1. Select the first mosquito identification method listed in the article from the drop-down menu.

BG. MOS_ID2. Select the second mosquito identification method listed in the article from the drop-down menu.

BH. MOS_ID3. Select the third mosquito identification method listed in the article from the drop-down menu.

BI. MOS_ID4. Select the fourth mosquito identification method listed in the article from the drop-down menu.

The following mosquito identification techniques can be selected: chromosomal banding sequences (“CBS”), cross mating experiment (“CME”), DNA probe (“DNA”), isoenzyme electrophoresis (“IE”), morphology (“M”), polymerase chain reaction method (“PCR”), salinity tolerance tests (“STT”) or unknown (“?”). If the identification technique is given but is not one of the methods listed or it is unclear, enter “?” (unknown) in MOS_ID1 and note it in the NOTES_VECTOR column. If it is not given in the article, enter “n.a.” (not answered). Do not assume that a particular method has been used unless specifically stated (in the case of morphological identification, if there is reference to identification keys, “morphology” can be assumed).

**Control fields**

BJ. CONTROL. Select “yes”/ “no”/“n.a.” from the drop-down menu.

BK. CONTROL_TYPE. If “yes” select control type from the drop-down menu.

These fields are used to indicate if control measures are being used in the area. For example if the article states that insecticide residual spraying is carried out annually in the area, list this as “yes” and “irs” in the control-type column and make a note of the details in the NOTES_VECTOR column. The fields can also be used to indicate if the study is part of an insecticide or bed net trial, for example, if one half of a village has been provided with insecticide treated bednets (control “yes”, control type “itn”), with the other side having untreated bednets (control “no”, control type “n.a.”). If you have two sets of data, record both pre- and post- control species abundances. Note any additional details that might be of use.

**Data entry ID**

The overall aim is accuracy and thoroughness NOT speed. All data will be checked by an independent data entry clerk and then by a data moderator.

BL. DEC_ID. Enter your initials in the data-entry clerk identification field.

BM. DEC_CHECK. Enter your initials in the data-entry clerk check field.

BN. MAP_CHECK. The data moderator will enter her initials after each datasheet has been reviewed, any queries resolved and before passing it on to be uploaded to the database.

**Other issues to consider**

BO. NOTES_VECTOR. This field is used to record any additional useful information. This can include queries to discuss with data moderators or information on other useful data sources *etc*. All other *Anopheles* malaria vectors that have been mentioned in the paper but are not in our 41 main vectors list should be listed here, in the appropriate dataline to correspond to the site where they were found. Additionally, note if you think the author may have useful data that are not in the publication and is worth contacting. Finally, if the article is not published in English, specify its language.

**General data information**

Consider each PDF as an independent, separate data source. For example if you come across two papers which are clearly describing the same study, or a journal publication taken from a thesis you have previously entered, enter the data from each article as if you are unaware of the other study. Do not make assumptions or use information you are aware of through reading other articles, unless specifically referenced in the article.

**Final notes and comments**

Use the notes columns to add information relevant to the study as these will be transferred to the database when the datasheets are downloaded. Use the “insert comment” function only for queries regarding data entry for the attention of whoever will be checking the datasheet – comments will not automatically be transferred onto the database.
