# Supplementary material for: Developing Global Maps of the Dominant Anopheles Vectors of Human Malaria
Source: PLoS Med. 2010 Feb 9;7(2):e1000209. doi: 10.1371/journal.pmed.1000209 (PMC2817710; doi:10.1371/journal.pmed.1000209)
Supplement: Text S3 — Maps of expert opinion distribution and species occurrence records for the dominant Anopheles vector species (and species complexes) of human malaria in the Americas. (1.82 MB PDF) [file pmed.1000209.s003.pdf]

Text S3. Maps of expert opinion distribution and species occurrence records for the dominant *Anopheles* vector species (and species complexes) of human malaria in the Americas.

The following maps are of the final nine dominant *Anopheles* vector species (DVS) and species complexes of human malaria in the Americas, as defined in the main text and Text S1. Each map is titled with the full scientific name (see Text S1). The maps are then arranged alphabetically by the species component of the full scientific name.

Each map shows an extent most appropriate to display the hypothesised range of each of the species or species complexes. The hypothesised range encompasses expert opinion (EO, in orange) and the species occurrence records collected from the searches of the formally published literature outlined in the main text and Text S2. The EO was digitised from a representative source and in every case substantially modified by; (i) incorporating verified occurrence records outside the EO; (ii) incorporating the advice of the technical advisory group and; (iii) taking into consideration known species-specific habitat requirements as revealed by elevation surfaces [1], satellite imagery [2] and land cover maps [3]. The occurrence records are coded grey for present and white for absent. They are mapped so that presence points overlay absences and multiple presence points in an area appear darker. The latter is important to convey the clustering of survey observations at the regional and continental scale of the maps. The first paragraph of the legend summarizes the number of presence and absence points displayed, along with the date range encompassed by those observations. Major rivers and other inland water bodies are shown in blue and coastlines and national borders in black. Latitude and longitude grids, scale bars and north arrows are presented on all maps along with a creative commons copyright declaration and suggested citation.

These maps are not the definitive product of this project. They are a useful first step in systematically gathering the species distribution information and are released with the EOs to highlight those areas where intensive searching of the informal literature and consultation of local experts is required. A note in each legend highlights areas where we are particularly keen to augment the occurrence records to provide the best “training data” and thus increase chances of accurately mapping the geographical distribution. The maps have been degraded to 200 dpi to restrict their size and thus facilitate rapid download and can be found at higher spatial resolution on the Malaria Atlas Project (MAP) website (http://www.map.ox.ac.uk).

References

1. Slater JA, Garvey G, Johnston C, Haase J, Heady B, et al. (2006) The SRTM data "finishing" process and products. Photogramm Eng Remote Sens 72: 237-247.
2. Scharlemann JPW, Benz D, Hay SI, Purse BV, Tatem AJ, et al. (2008) Global data for ecology and epidemiology: a novel algorithm for temporal Fourier processing MODIS data. PLoS One 3: e1408.
3. Bicheron P, Defourny P, Brockmann C, Vancutsem C, Huc M, et al. (2008) GLOBCOVER: Products Description and Validation Report Toulouse, France MEDIAS-France.

Anopheles (*Nyssorhynchus*) *albimanus* Wiedemann, 1820

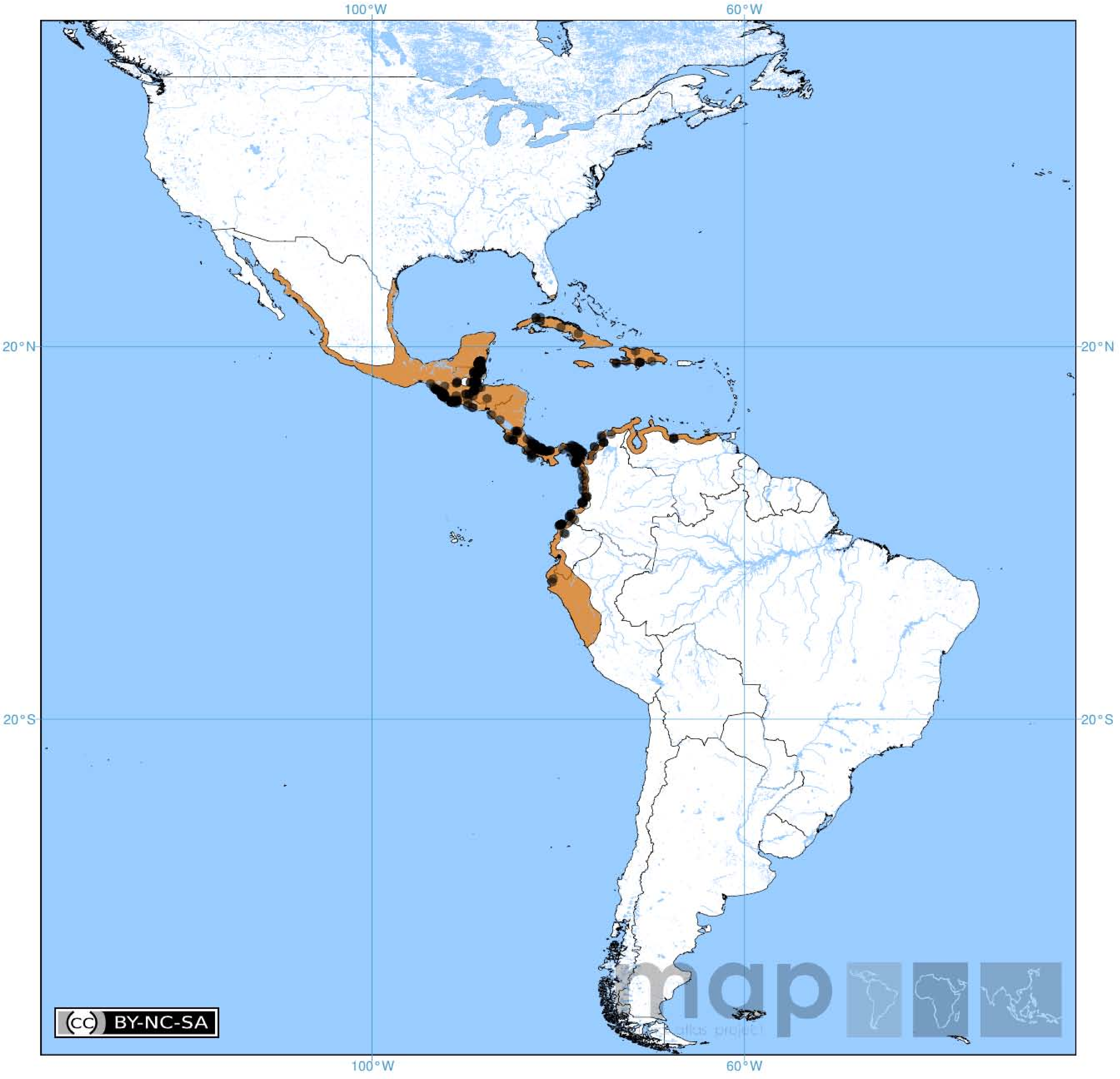

0 5000 10000 Kilometres

The 445 records for *Anopheles* (*Nyssorhynchus*) *albimanus* Wiedemann, 1820, were found in 16 countries between 1983 and 2006. There were 364 records of occurrence and 81 records of true absence.

Note: We are particularly keen to augment this map with occurrence records from the northerly extremes of the distribution in Mexico, as well as with information from Nicaragua, Jamaica and the southern and western reaches of its range in Peru and Venezuela respectively. Please e-mail map.vector@zoo.ox.ac.uk if you have any useful information to share on the distribution of this species.

Copyright: Licensed to the Malaria Atlas Project (MAP; www.map.ox.ac.uk) under a Creative Commons Attribution 3.0 License (http://creativecommons.org/).

Citation: Hay, S.I. *et al.* (2009). Developing global maps of the dominant *Anopheles* vectors of human malaria. *PLoS Medicine* **6**: in press.

EO range

Present

Absent

N

Anopheles (*Nyssorhynchus*) *albitarsis*

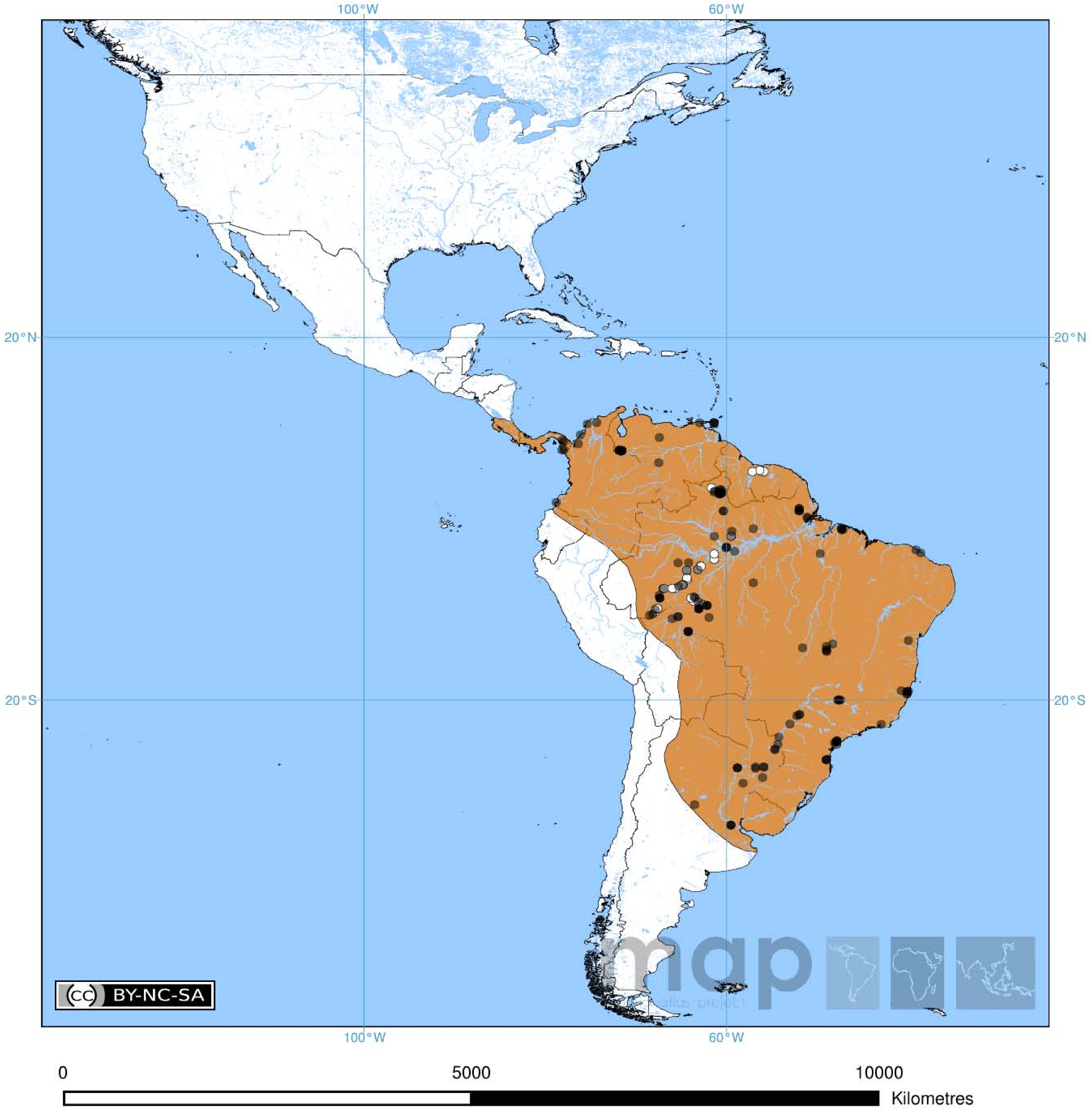

The 191 records for *Anopheles* (*Nyssorhynchus*) *albitarsis*, were found in 9 countries between 1983 and 2006. There were 161 records of occurrence and 30 records of true absence.

Note: We are particularly keen to augment this map with occurrence records from the northern extent of the distribution in Costa Rica and Panama, as well as from its south westerly extent in Colombia, Brazil, Bolivia and Argentina. Please e-mail map.vector@zoo.ox.ac.uk if you have any useful information to share on the distribution of this species complex. This species complex is to be further stratified with focus on *An. albitarsis* (Sp. A), *An. oryzalimnetes* (Sp. B), *An. deaneorum* (Sp. D), *An. janconnae* (Sp. E) and *An. albitarsis* (Sp. F). *An. marajoara* (Sp. C) is an established, known vector and is therefore mapped separately.

N

EO range

Present

Absent

Copyright: Licensed to the Malaria Atlas Project (MAP; www.map.ox.ac.uk) under a Creative Commons Attribution 3.0 License (http://creativecommons.org/).

Citation: Hay, S.I. *et al.* (2009). Developing global maps of the dominant *Anopheles* vectors of human malaria. *PLoS Medicine* **6**: in press.

Anopheles (*Nyssorhynchus*) *aquasalis* Curry, 1932

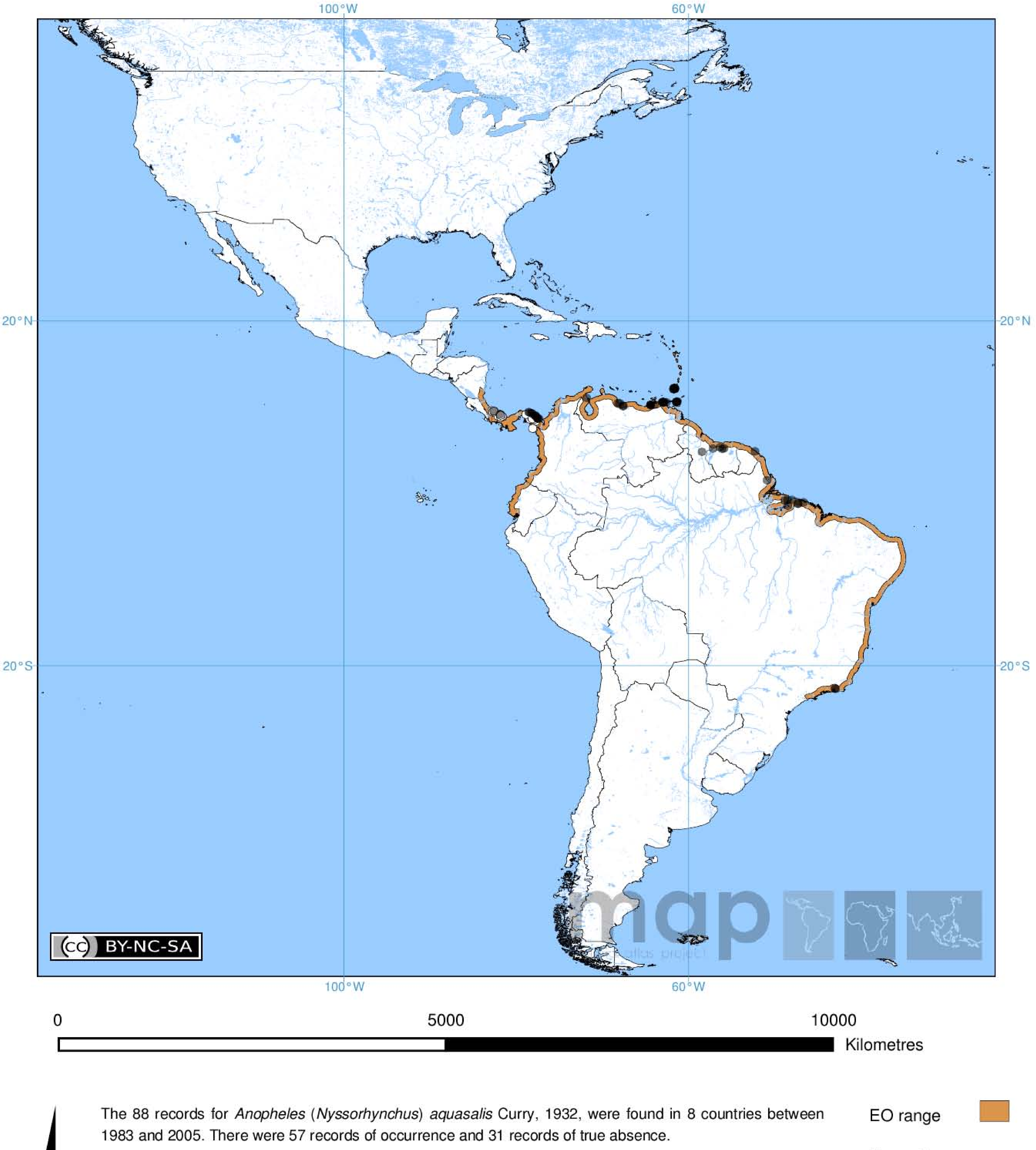

The 88 records for *Anopheles* (*Nyssorhynchus*) *aquasalis* Curry, 1932, were found in 8 countries between 1983 and 2005. There were 57 records of occurrence and 31 records of true absence.

Note: We are particularly keen to augment this map with occurrence records from the northern extent of the distribution in Costa Rica and Nicaragua, as well as from its southern reaches in Ecuador and Brazil respectively. Please e-mail map.vector@zoo.ox.ac.uk if you have any useful information to share on the distribution of this species.

EO range

Present

Absent

Copyright: Licensed to the Malaria Atlas Project (MAP; www.map.ox.ac.uk) under a Creative Commons Attribution 3.0 License (http://creativecommons.org/).

Citation: Hay, S.I. *et al.* (2009). Developing global maps of the dominant *Anopheles* vectors of human malaria. *PLoS Medicine* **6**: in press.

Anopheles (*Nyssorhynchus*) *darlingi* Root, 1926

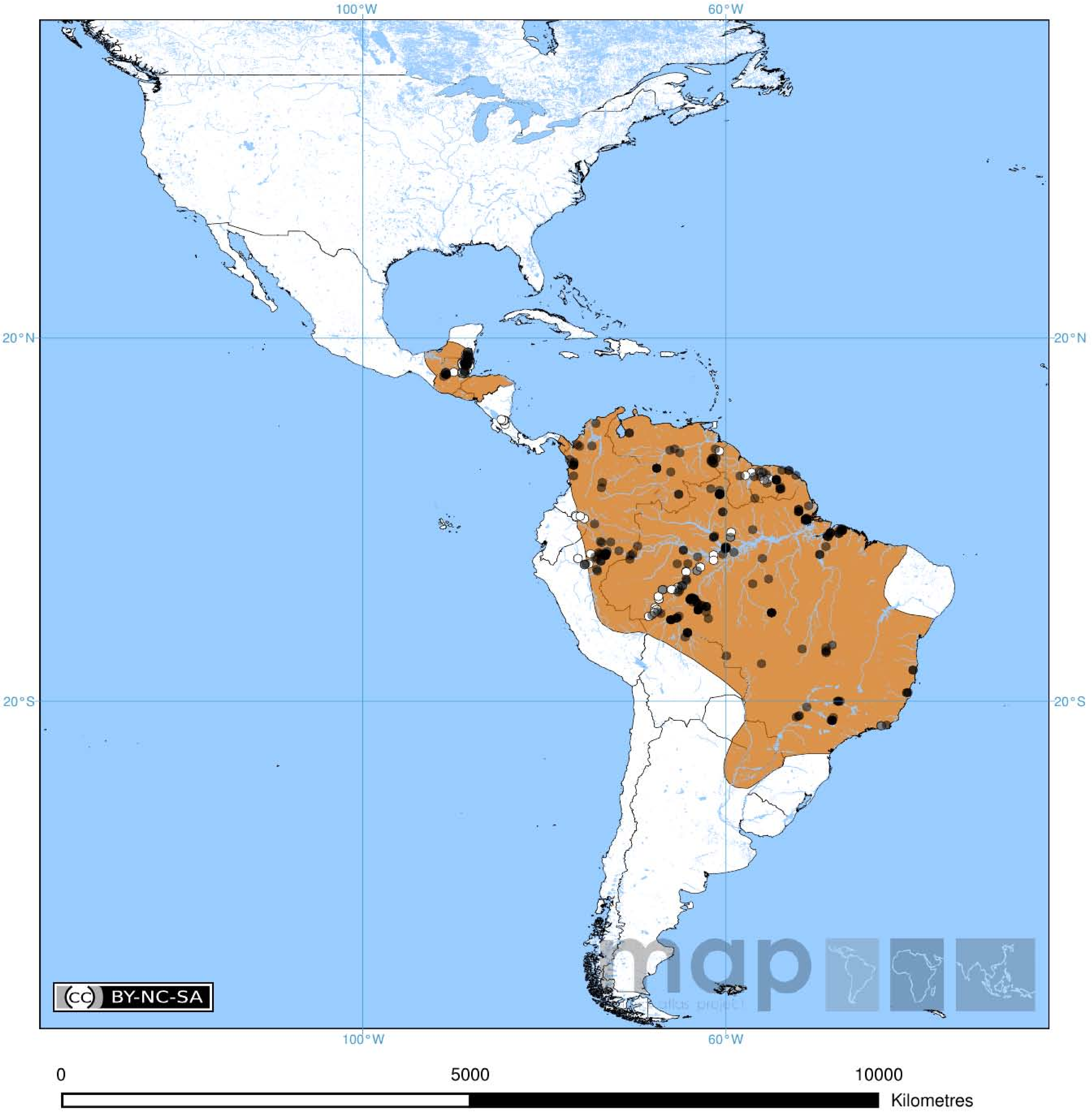

The 513 records for *Anopheles* (*Nyssorhynchus*) *darlingi* Root, 1926, were found in 12 countries between 1983 and 2007. There were 318 records of occurrence and 195 records of true absence.

Note: We are particularly keen to augment this map with occurrence records from the northern extent of the species range in southern Mexico, Belize, Guatemala, El Salvador and Honduras, as well as with records from the southerly extent in Bolivia, Brazil, Paraguay and Argentina. Please e-mail map.vector@zoo.ox.ac.uk if you have any useful information to share on the distribution of this species.

Copyright: Licensed to the Malaria Atlas Project (MAP; www.map.ox.ac.uk) under a Creative Commons Attribution 3.0 License (http://creativecommons.org/).

Citation: Hay, S.I. *et al.* (2009). Developing global maps of the dominant *Anopheles* vectors of human malaria. *PLoS Medicine* **6**: in press.

EO range

Present

Absent

Anopheles (*Anopheles*) *freeborni* Aitken, 1939

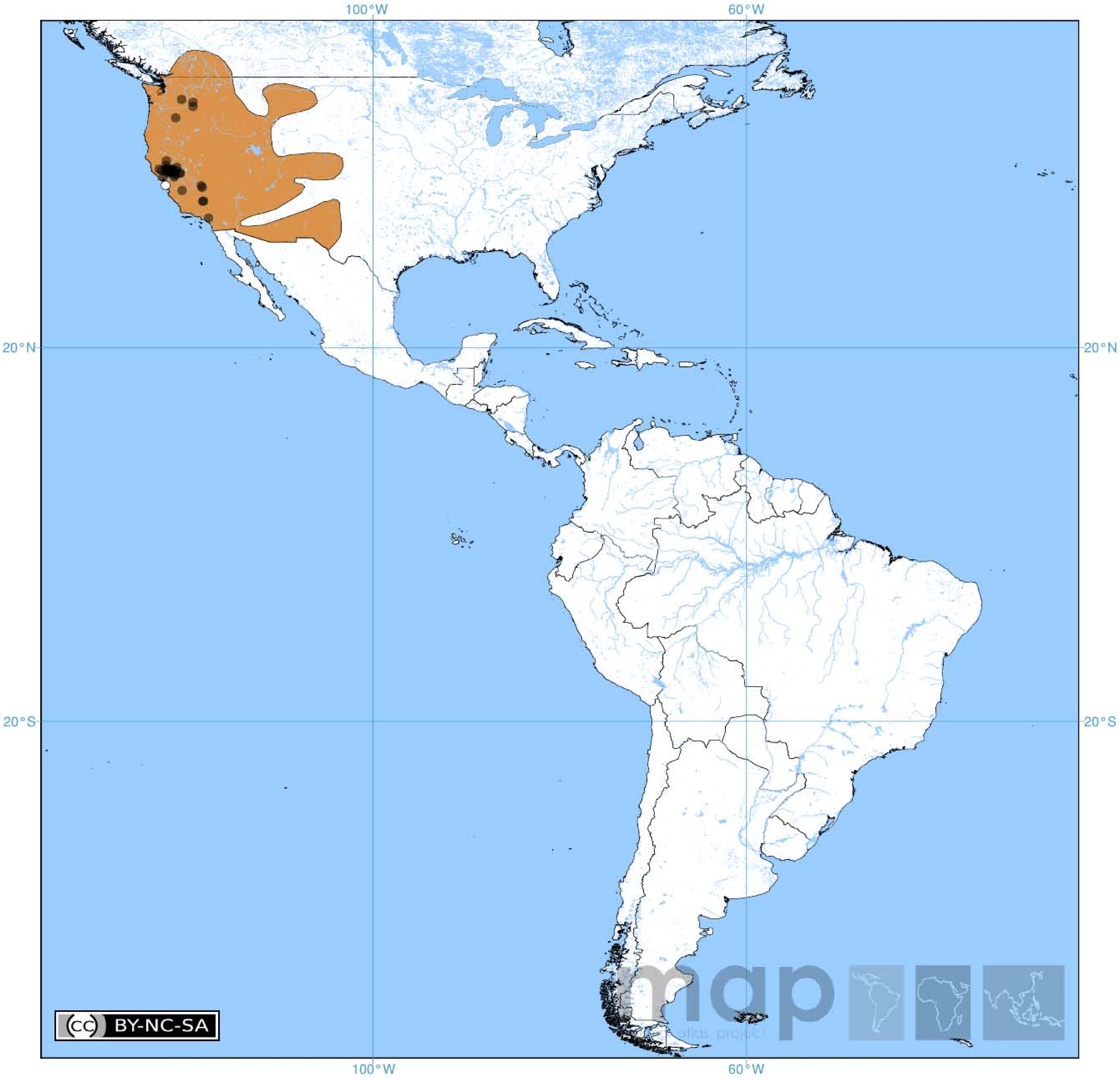

0 5000 10000
Kilometres

N

The 38 records for *Anopheles* (*Anopheles*) *freeborni* Aitken, 1939, were found in 1 countries between 1985 and 1990. There were 37 records of occurrence and 1 records of true absence.

Note: We are particularly keen to augment this map with occurrence records from the eastern extent of the species range in the United States of America. Please e-mail map.vector@zoo.ox.ac.uk if you have any useful information to share on the distribution of this species.

Copyright: Licensed to the Malaria Atlas Project (MAP; www.map.ox.ac.uk) under a Creative Commons Attribution 3.0 License (http://creativecommons.org/).

Citation: Hay, S.I. *et al.* (2009). Developing global maps of the dominant *Anopheles* vectors of human malaria. *PLoS Medicine* **6**: in press.

EO range

Present

Absent

Anopheles (*Nyssorhynchus*) *marajoara* Galvão & Damasceno, 1942

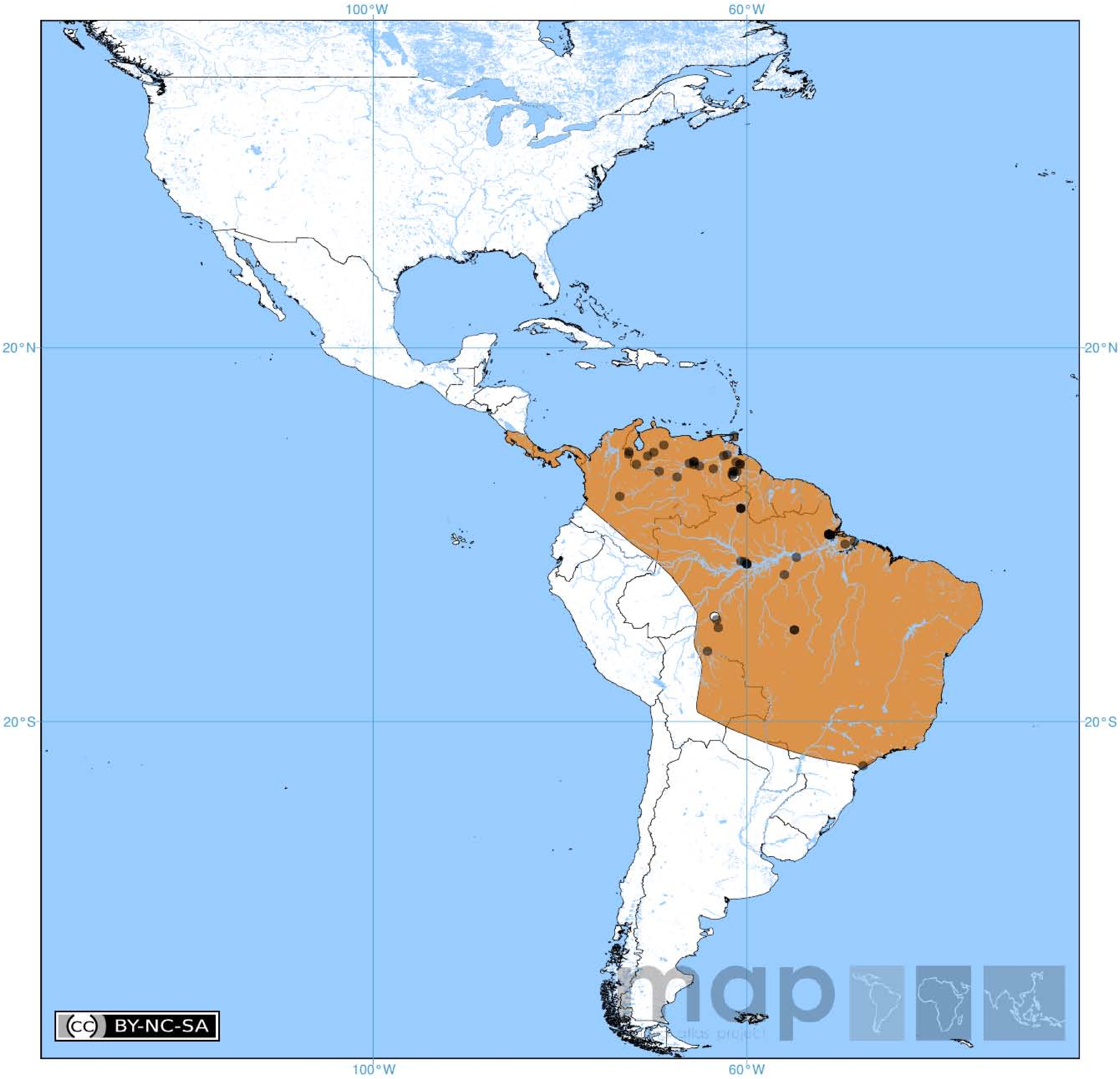

0 5000 10000
Kilometres

The 58 records for *Anopheles* (*Nyssorhynchus*) *marajoara* Galvão & Damasceno, 1942, were found in 4 countries between 1988 and 2005. There were 55 records of occurrence and 3 records of true absence.

Note: We are particularly keen to augment this map with occurrence records from the northern extent of the distribution in Costa Rica, Panama, Colombia, Guyana, Suriname and French Guiana, as well as, its southerly extent in Bolivia, Paraguay and Brazil. Please e-mail map.vector@zoo.ox.ac.uk if you have any useful information to share on the distribution of this species.

Copyright: Licensed to the Malaria Atlas Project (MAP; www.map.ox.ac.uk) under a Creative Commons Attribution 3.0 License (http://creativecommons.org/).

Citation: Hay, S.I. *et al.* (2009). Developing global maps of the dominant *Anopheles* vectors of human malaria. *PLoS Medicine* **6**: in press.

EO range

Present

Absent

N

Anopheles (*Nyssorhynchus*) *nuneztovari*

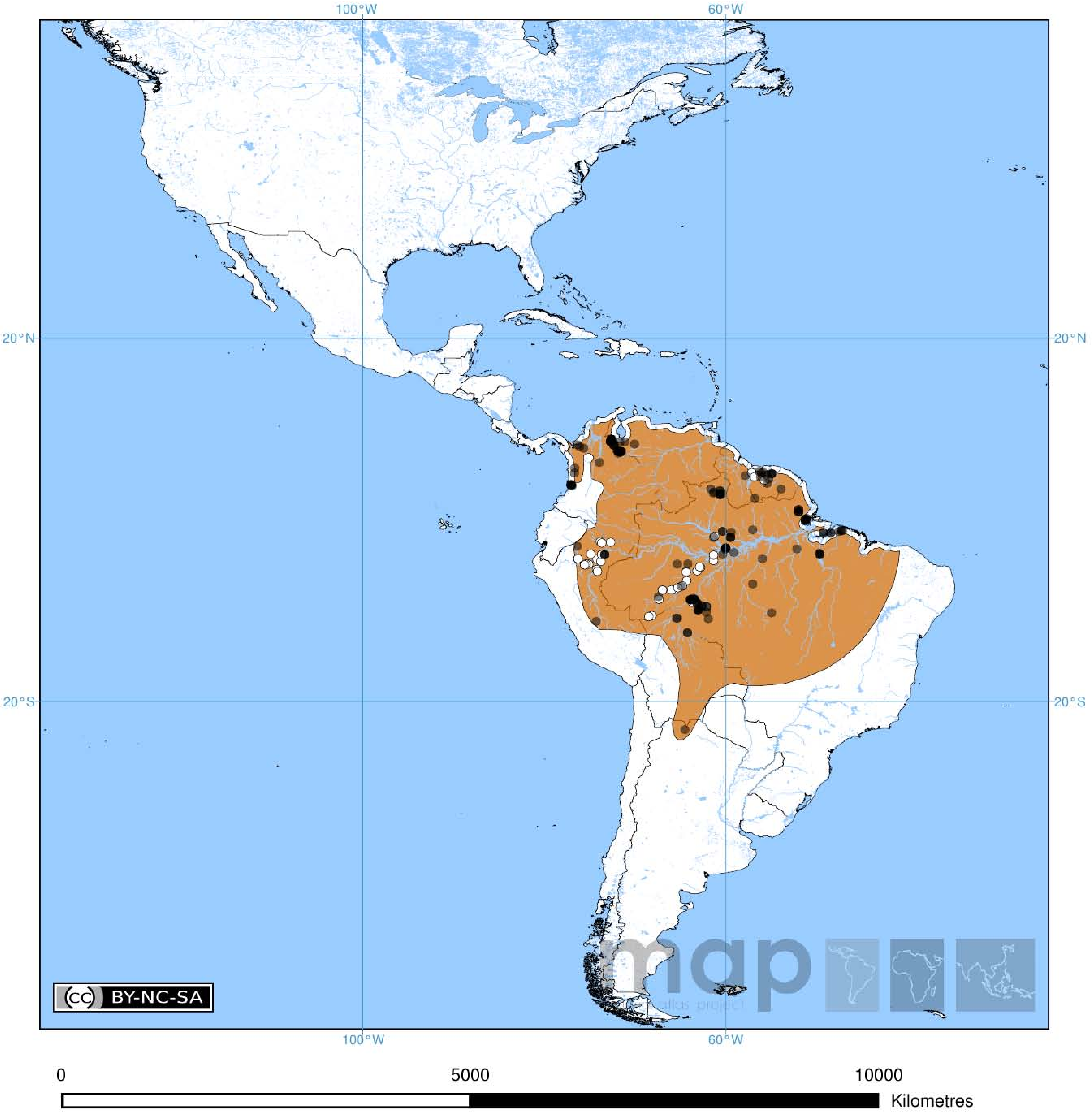

The 210 records for *Anopheles* (*Nyssorhynchus*) *nuneztovari*, were found in 8 countries between 1983 and 2007. There were 171 records of occurrence and 39 records of true absence.

Note: We are particularly keen to augment this map with occurrence records from the northern extent of the distribution in Colombia, and Venezuela, as well as in its southerly extent in Bolivia and Brazil. Please e-mail map.vector@zoo.ox.ac.uk if you have any useful information to share on the distribution of this species complex.

Copyright: Licensed to the Malaria Atlas Project (MAP; www.map.ox.ac.uk) under a Creative Commons Attribution 3.0 License (http://creativecommons.org/).

Citation: Hay, S.I. *et al.* (2009). Developing global maps of the dominant *Anopheles* vectors of human malaria. *PLoS Medicine* **6**: in press.

EO range
Present
Absent

N

Anopheles (*Anopheles*) *pseudopunctipennis*

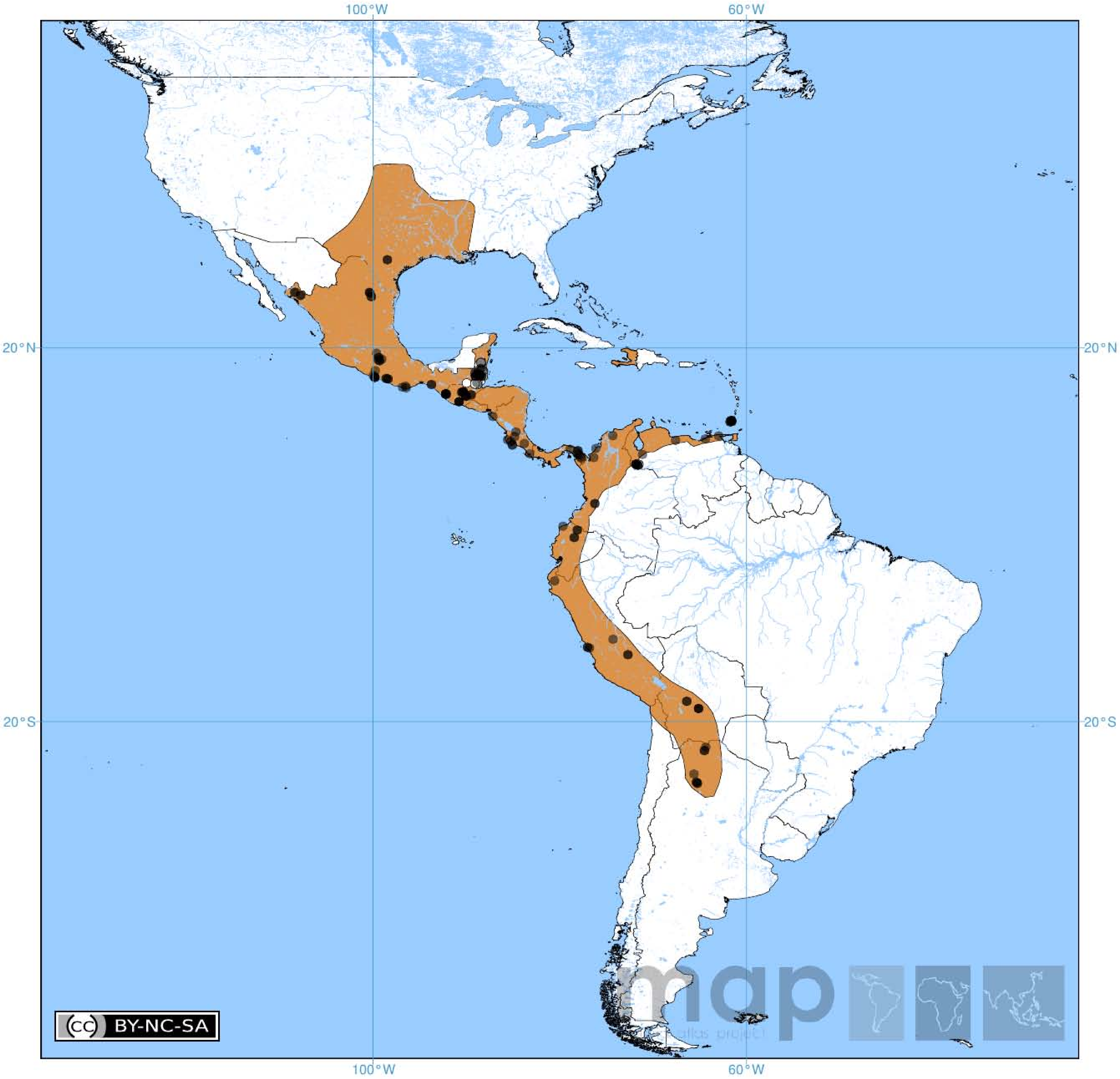

0 5000 10000
Kilometres

The 292 records for *Anopheles* (*Anopheles*) *pseudopunctipennis*, were found in 15 countries between 1985 and 2006. There were 157 records of occurrence and 135 records of true absence.

Note: We are particularly keen to augment this map with occurrence records from its northern extent in the United States of America and Mexico, as well as, Honduras, Nicaragua, Chile and the east coast of Peru. Please e-mail map.vector@zoo.ox.ac.uk if you have any useful information to share on the distribution of this species complex.

Copyright: Licensed to the Malaria Atlas Project (MAP; www.map.ox.ac.uk) under a Creative Commons Attribution 3.0 License (http://creativecommons.org/).

Citation: Hay, S.I. *et al.* (2009). Developing global maps of the dominant *Anopheles* vectors of human malaria. *PLoS Medicine* **6**: in press.

N

EO range

Present

Absent

Anopheles (*Anopheles*) *quadrimaculatus* Say, 1824

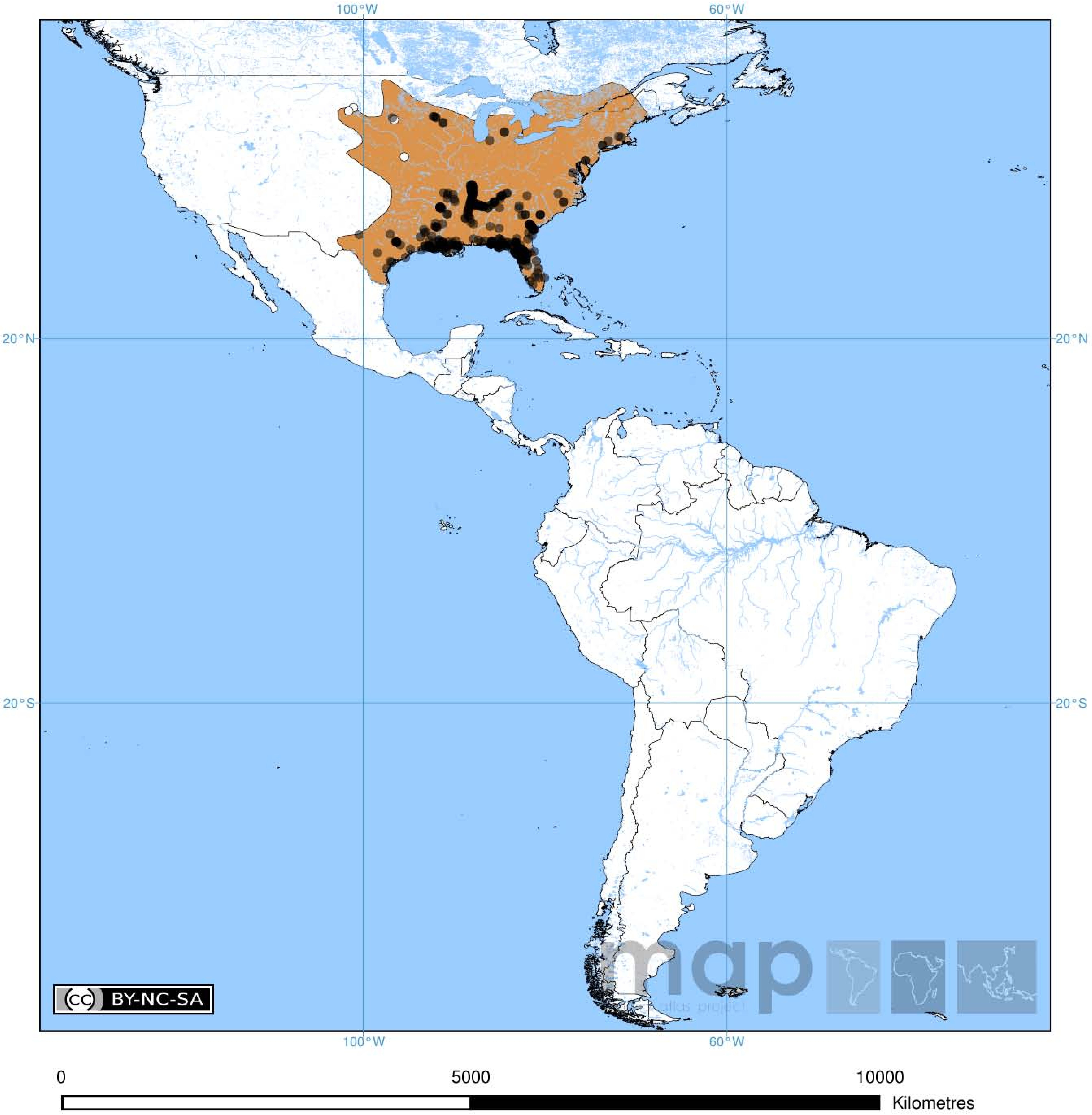

The 383 records for *Anopheles* (*Anopheles*) *quadrimaculatus* Say, 1824, were found in 3 countries between 1983 and 2005. There were 379 records of occurrence and 4 records of true absence.

Note: We are particularly keen to augment this map with occurrence records from its central and northern distribution in the United States of America including Kansas, Missouri, Illinois, Indiana, Ohio, Pennsylvania, new York and Maine, as well as, Quebec and Ontario in Canada. Please e-mail map.vector@zoo.ox.ac.uk if you have any useful information to share on the distribution of this species.

Copyright: Licensed to the Malaria Atlas Project (MAP; www.map.ox.ac.uk) under a Creative Commons Attribution 3.0 License (http://creativecommons.org/).

Citation: Hay, S.I. *et al.* (2009). Developing global maps of the dominant *Anopheles* vectors of human malaria. *PLoS Medicine* **6**: in press.

N

EO range

Present

Absent
