# Supplementary material for: Developing Global Maps of the Dominant Anopheles Vectors of Human Malaria
Source: PLoS Med. 2010 Feb 9;7(2):e1000209. doi: 10.1371/journal.pmed.1000209 (PMC2817710; doi:10.1371/journal.pmed.1000209)
Supplement: Text S4 — Maps of expert opinion distribution and species occurrence records for the dominant Anopheles vector species (and species complexes) of human malaria in Africa, Europe, and the Middle East. (3.02 MB PDF) [file pmed.1000209.s004.pdf]

Text S4. Maps of expert opinion distribution and species occurrence records for the dominant *Anopheles* vector species (and species complexes) of human malaria in Africa, Europe and the Middle East.

The following maps are of the final 13 dominant *Anopheles* vector species (DVS) and species complexes of human malaria in Africa, Europe and Middle East, as defined in the main text and Text S1. Each map is titled with the full scientific name (see Text S1). The maps are then arranged alphabetically by the species component of the full scientific name.

Each map shows an extent most appropriate to display the hypothesised range of each of the species or species complexes. The hypothesised range encompasses expert opinion (EO, in orange) and the species occurrence records collected from the searches of the formally published literature outlined in the main text and Text S2. The EO was digitised from a representative source and in every case substantially modified by; (i) incorporating verified occurrence records outside the EO; (ii) incorporating the advice of the technical advisory group and; (iii) taking into consideration known species-specific habitat requirements as revealed by elevation surfaces [1], satellite imagery [2] and land cover maps [3]. The occurrence records are coded grey for present and white for absent. They are mapped so that presence points overlay absences and multiple presence points in an area appear darker. The latter is important to convey the clustering of survey observations at the regional and continental scale of the maps. The first paragraph of the legend summarizes the number of presence and absence points displayed, along with the date range encompassed by those observations. Major rivers and other inland water bodies are shown in blue and coastlines and national borders in black. Latitude and longitude grids, scale bars and north arrows are presented on all maps along with a creative commons copyright declaration and suggested citation.

These maps are not the definitive product of this project. They are a useful first step in systematically gathering the species distribution information and are released with the EOs to highlight those areas where intensive searching of the informal literature and consultation of local experts is required. A note in each legend highlights areas where we are particularly keen to augment the occurrence records to provide the best “training data” and thus increase chances of accurately mapping the geographical distribution. The maps have been degraded to 200 dpi to restrict their size and thus facilitate rapid download and can be found at higher spatial resolution on the Malaria Atlas Project (MAP) website (http://www.map.ox.ac.uk).

References

1. Slater JA, Garvey G, Johnston C, Haase J, Heady B, et al. (2006) The SRTM data "finishing" process and products. Photogramm Eng Remote Sens 72: 237-247.
2. Scharlemann JPW, Benz D, Hay SI, Purse BV, Tatem AJ, et al. (2008) Global data for ecology and epidemiology: a novel algorithm for temporal Fourier processing MODIS data. PLoS One 3: e1408.
3. Bicheron P, Defourny P, Brockmann C, Vancutsem C, Huc M, et al. (2008) GLOBCOVER: Products Description and Validation Report Toulouse, France MEDIAS-France.

Anopheles (*Cellia*) *arabiensis* Patton, 1905

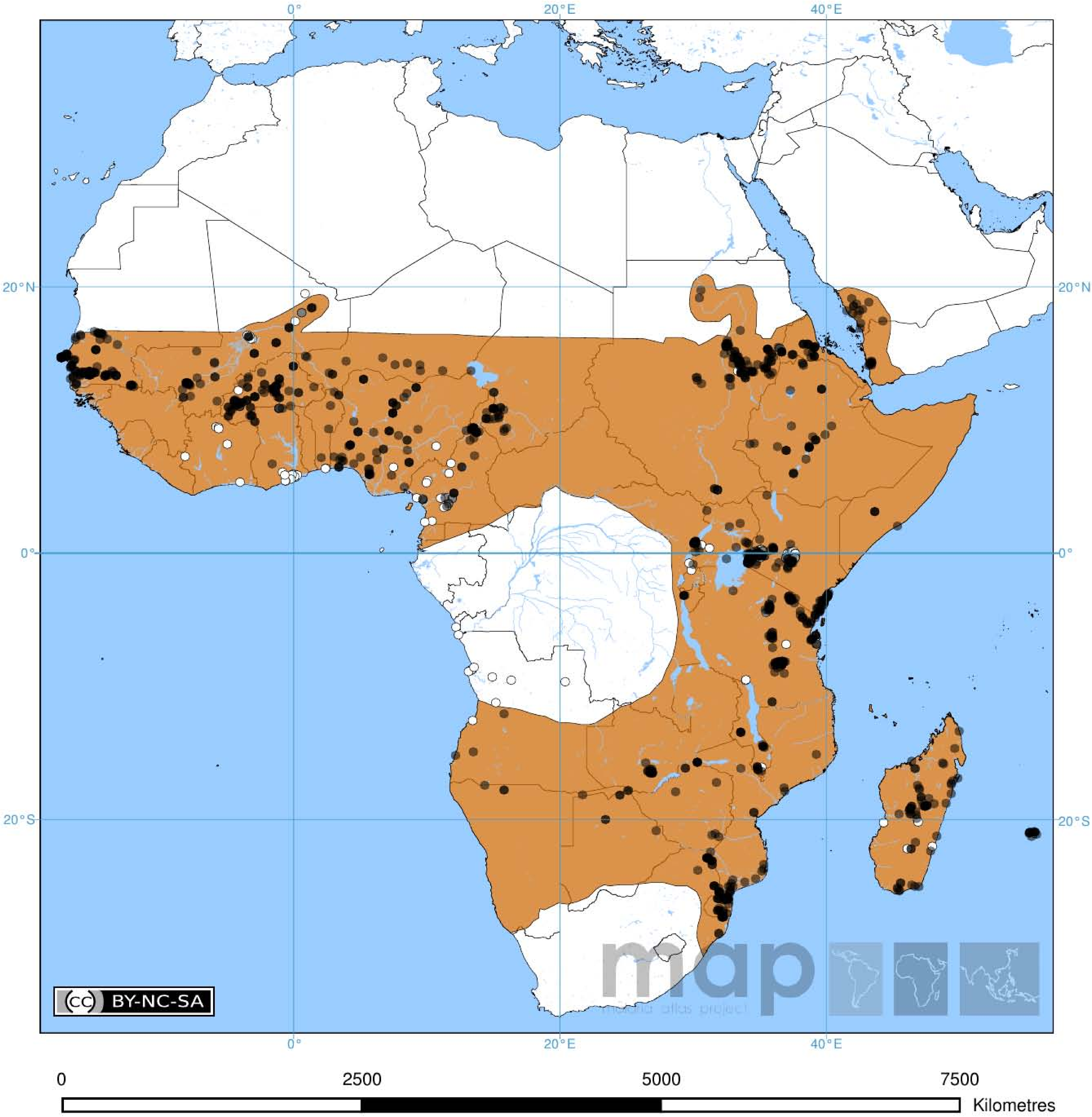

N

EO range

Present

Absent

The 1353 records for *Anopheles* (*Cellia*) *arabiensis* Patton, 1905, were found in 32 countries between 1974 and 2008. There were 1194 records of occurrence and 159 records of true absence.

Note: We are particularly keen to augment this map with occurrence records from the northern extent of the distribution in the Sahel, the Central African Republic, as well as from its southern reaches in Botswana, Namibia and Angola. Please e-mail map.vector@zoo.ox.ac.uk if you have any useful information to share on the distribution of this species.

Copyright: Licensed to the Malaria Atlas Project (MAP; www.map.ox.ac.uk) under a Creative Commons Attribution 3.0 License (http://creativecommons.org/).

Citation: Hay, S.I. *et al.* (2009). Developing global maps of the dominant *Anopheles* vectors of human malaria. *PLoS Medicine* **6**: in press.

Anopheles (*Anopheles*) *atroparvus* van Thiel, 1927

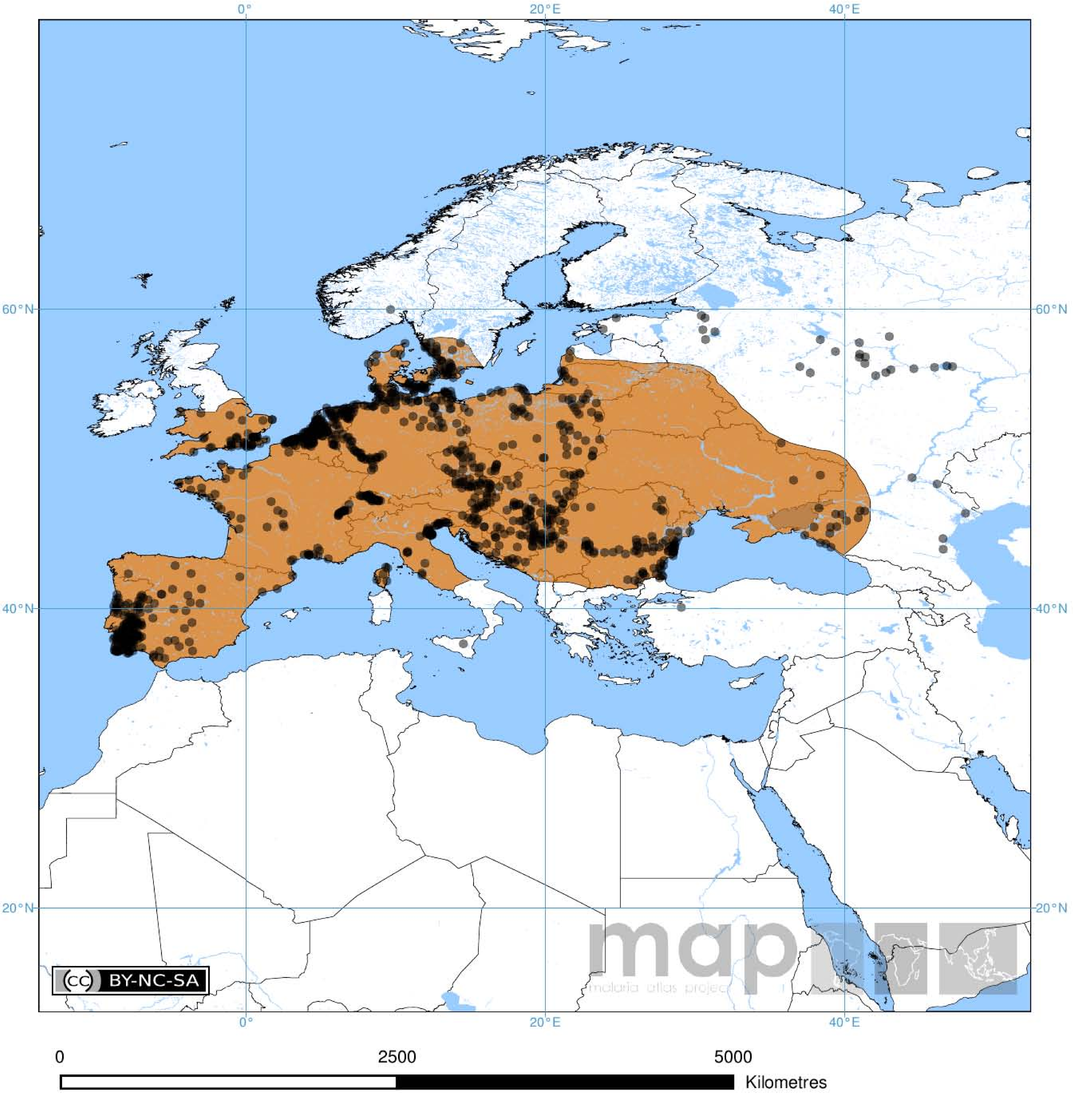

The 1049 records for *Anopheles* (*Anopheles*) *atroparvus* van Thiel, 1927, were found in 30 countries between 1982 and 2006. There were 1044 records of occurrence and 5 records of true absence.

Note: We are particularly keen to augment this map with occurrence records from Germany and the eastern extent of the distribution in Lithuania, Belarus, Ukraine and Romania. Please e-mail map.vector@zoo.ox.ac.uk if you have any useful information to share on the distribution of this species.

Copyright: Licensed to the Malaria Atlas Project (MAP; www.map.ox.ac.uk) under a Creative Commons Attribution 3.0 License (http://creativecommons.org/).

Citation: Hay, S.I. *et al.* (2009). Developing global maps of the dominant *Anopheles* vectors of human malaria. *PLoS Medicine* **6**: in press.

EO range

Present

Absent

Anopheles (*Cellia*) *funestus* Giles, 1900

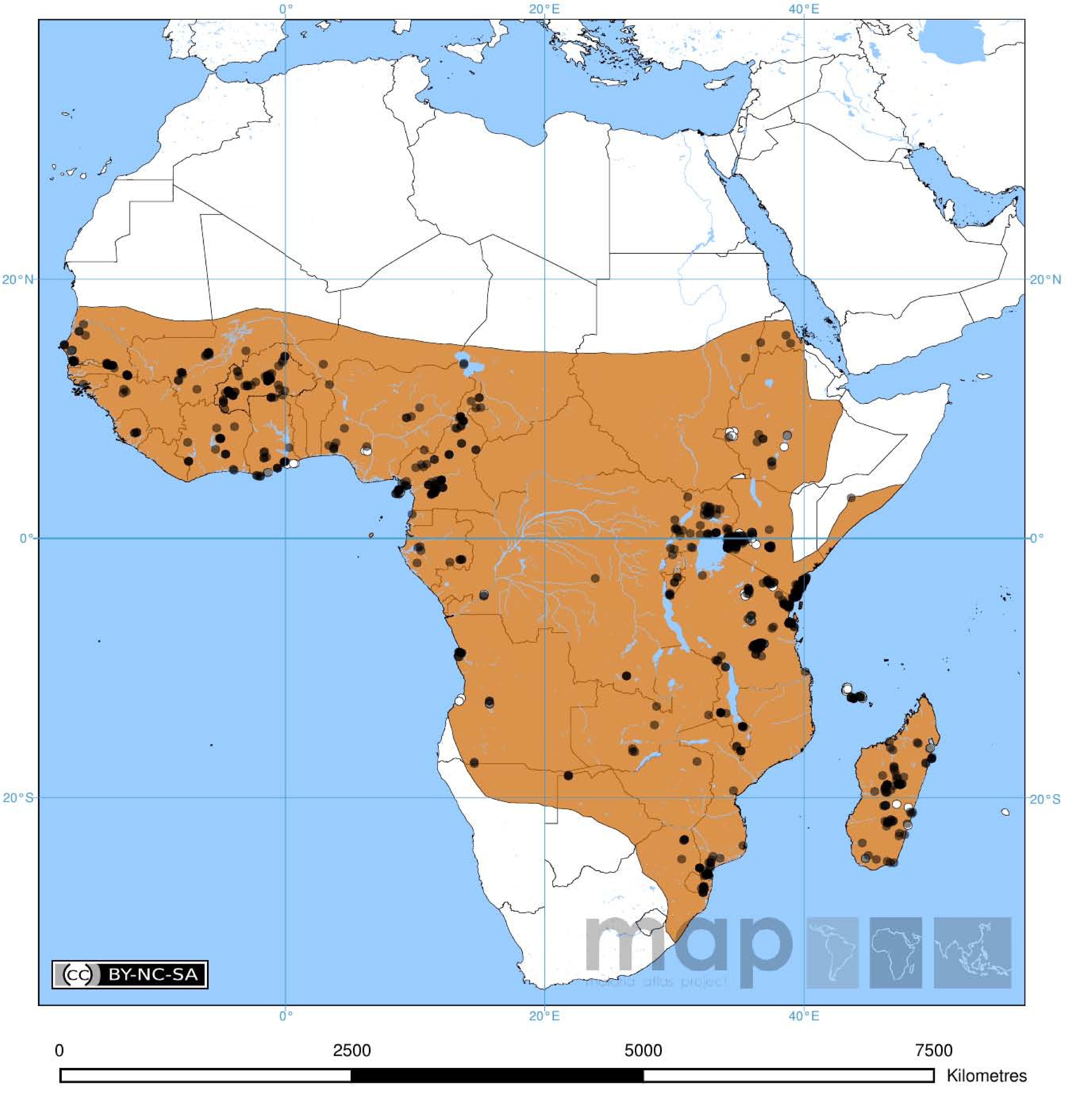

The 1039 records for *Anopheles* (*Cellia*) *funestus* Giles, 1900, were found in 31 countries between 1980 and 2008. There were 920 records of occurrence and 119 records of true absence.

EO range

Present

Absent

Note: We are particularly keen to augment this map with occurrence records from the northern extent of the distribution in the Sahel, the Central African Republic, and the Democratic republic of Congo, Ethiopia, as well as, its southern reaches in South Africa, Botswana, Namibia and Angola. Please e-mail map.vector@zoo.ox.ac.uk if you have any useful information to share on the distribution of this species.

Copyright: Licensed to the Malaria Atlas Project (MAP; www.map.ox.ac.uk) under a Creative Commons Attribution 3.0 License (http://creativecommons.org/).

Citation: Hay, S.I. *et al.* (2009). Developing global maps of the dominant *Anopheles* vectors of human malaria. *PLoS Medicine* **6**: in press.

Anopheles (*Cellia*) *gambiae* Giles, 1902

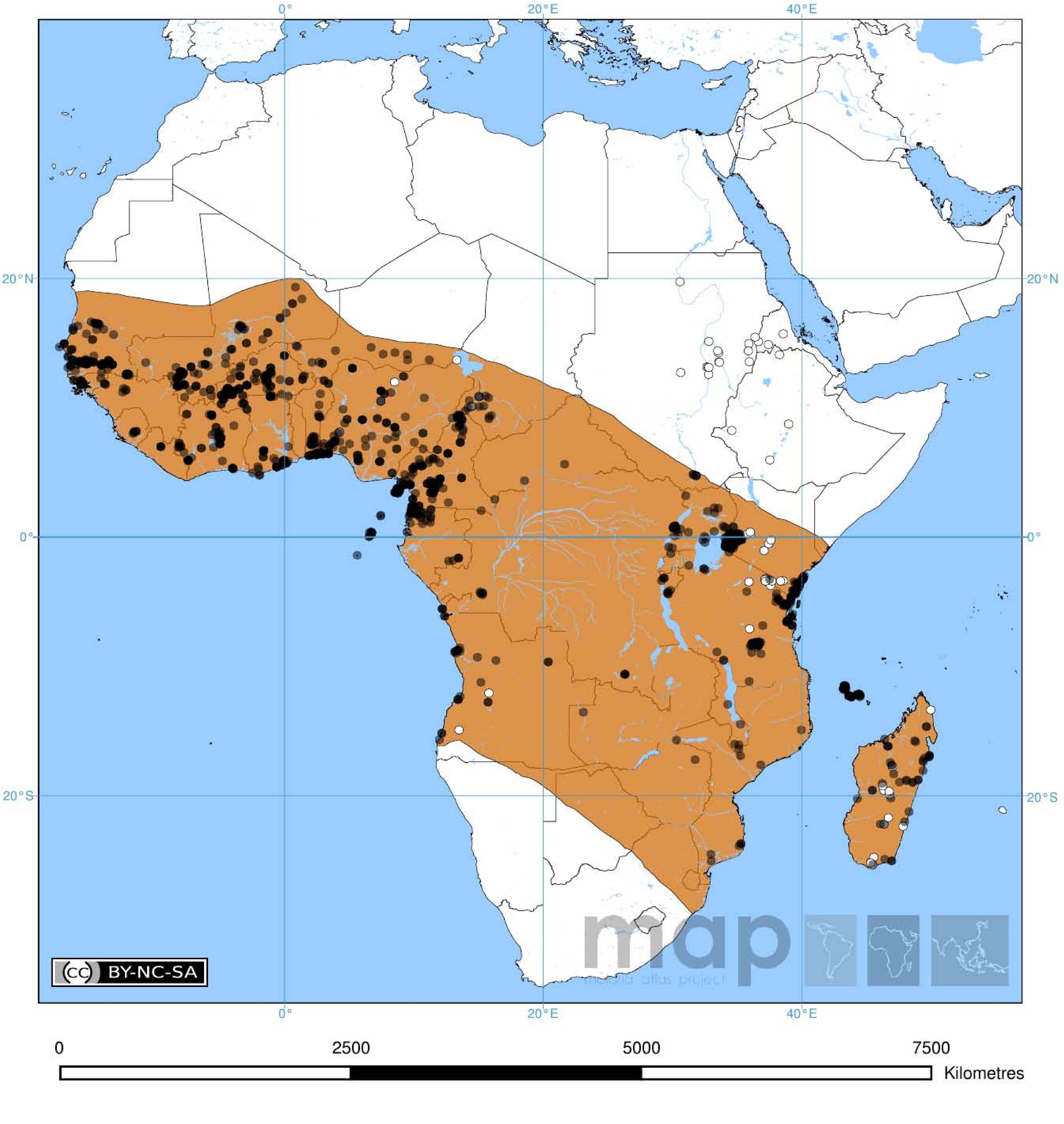

The 1515 records for *Anopheles* (*Cellia*) *gambiae* Giles, 1902, were found in 36 countries between 1974 and 2009. There were 1443 records of occurrence and 72 records of true absence.

EO range

Present

Absent

Note: We are particularly keen to augment this map with occurrence records from the Democratic republic of Congo, as well as from its southern reaches in South Africa, Botswana and Namibia. Please e-mail map.vector@zoo.ox.ac.uk if you have any useful information to share on the distribution of this species.

Copyright: Licensed to the Malaria Atlas Project (MAP; www.map.ox.ac.uk) under a Creative Commons Attribution 3.0 License (http://creativecommons.org/).

Citation: Hay, S.I. *et al.* (2009). Developing global maps of the dominant *Anopheles* vectors of human malaria. *PLoS Medicine* **6**: in press.

Anopheles (*Anopheles*) *labranchiae* Falleroni, 1926

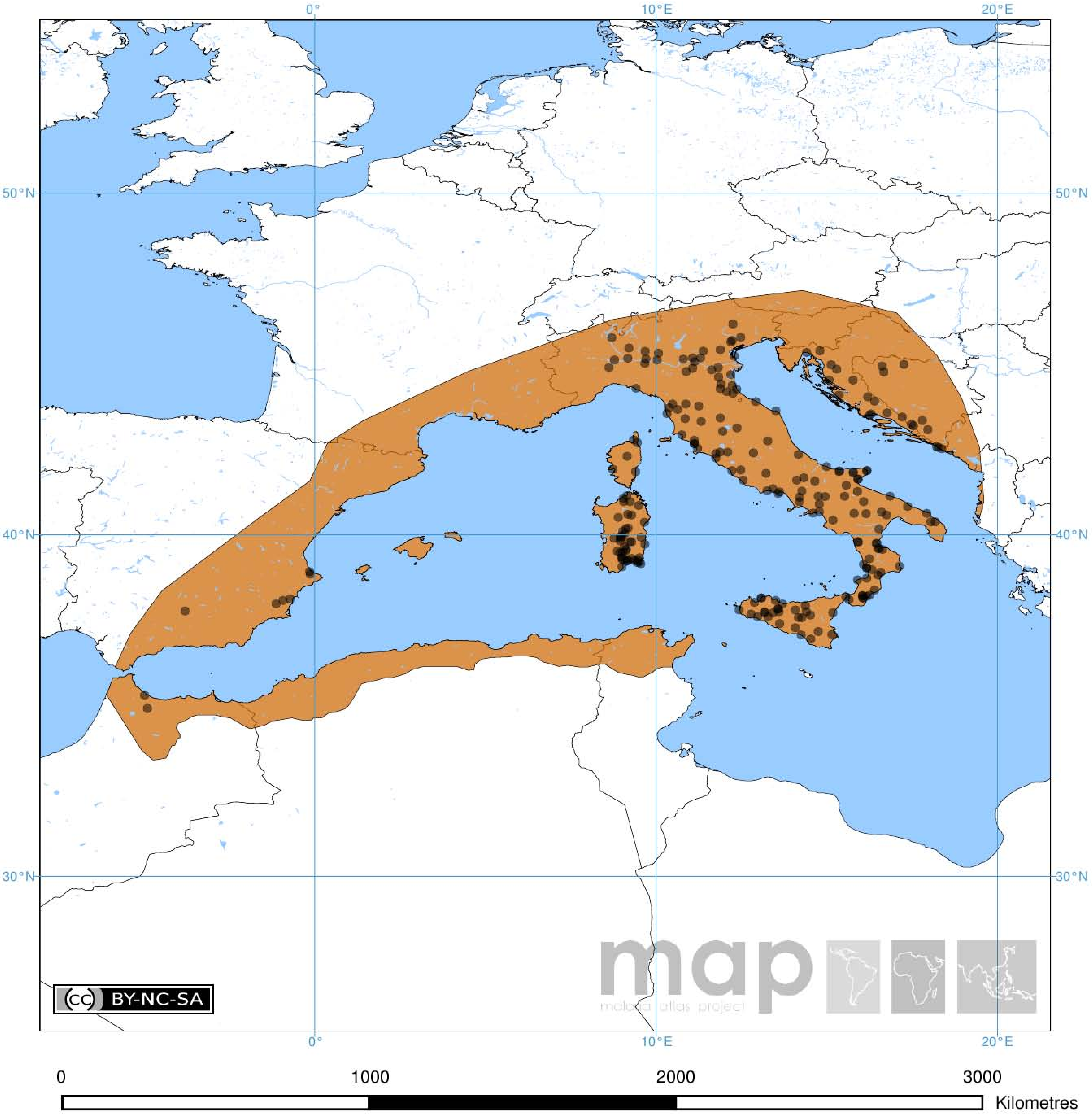

N

The 234 records for *Anopheles* (*Anopheles*) *labranchiae* Falleroni, 1926, were found in 6 countries between 1992 and 2005. There were 234 records of occurrence and 0 records of true absence.

Note: We are particularly keen to augment this map with occurrence records from Morocco, Algeria and Tunisia. Please e-mail map.vector@zoo.ox.ac.uk if you have any useful information to share on the distribution of this species.

Copyright: Licensed to the Malaria Atlas Project (MAP; www.map.ox.ac.uk) under a Creative Commons Attribution 3.0 License (http://creativecommons.org/).

Citation: Hay, S.I. *et al.* (2009). Developing global maps of the dominant *Anopheles* vectors of human malaria. *PLoS Medicine* **6**: in press.

EO range

Present

Absent

Anopheles (*Cellia*) *melas* Theobald, 1903

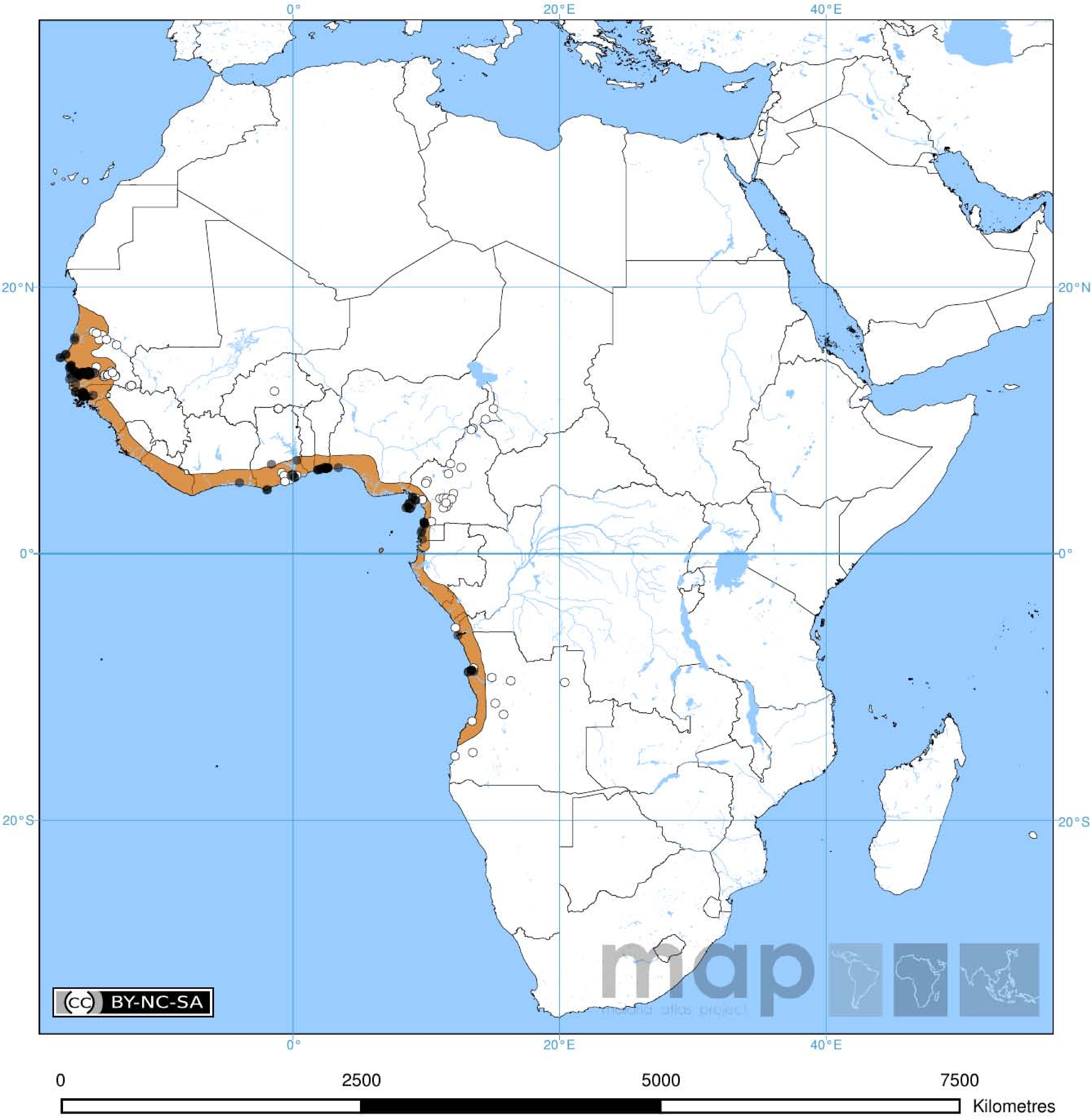

N

The 238 records for *Anopheles* (*Cellia*) *melas* Theobald, 1903, were found in 11 countries between 1982 and 2007. There were 149 records of occurrence and 89 records of true absence.

Note: We are particularly keen to augment this map with occurrence records from the coastal distribution of this species in Mauritania, Guinea, Sierra Leone, Liberia, Nigeria, Equatorial Guinea, Gabon, Congo and Angola. Please e-mail map.vector@zoo.ox.ac.uk if you have any useful information to share on the distribution of this species.

Copyright: Licensed to the Malaria Atlas Project (MAP; www.map.ox.ac.uk) under a Creative Commons Attribution 3.0 License (http://creativecommons.org/).

Citation: Hay, S.I. *et al.* (2009). Developing global maps of the dominant *Anopheles* vectors of human malaria. *PLoS Medicine* **6**: in press.

EO range

Present

Absent

Anopheles (*Cellia*) *merus* Dönitz, 1902

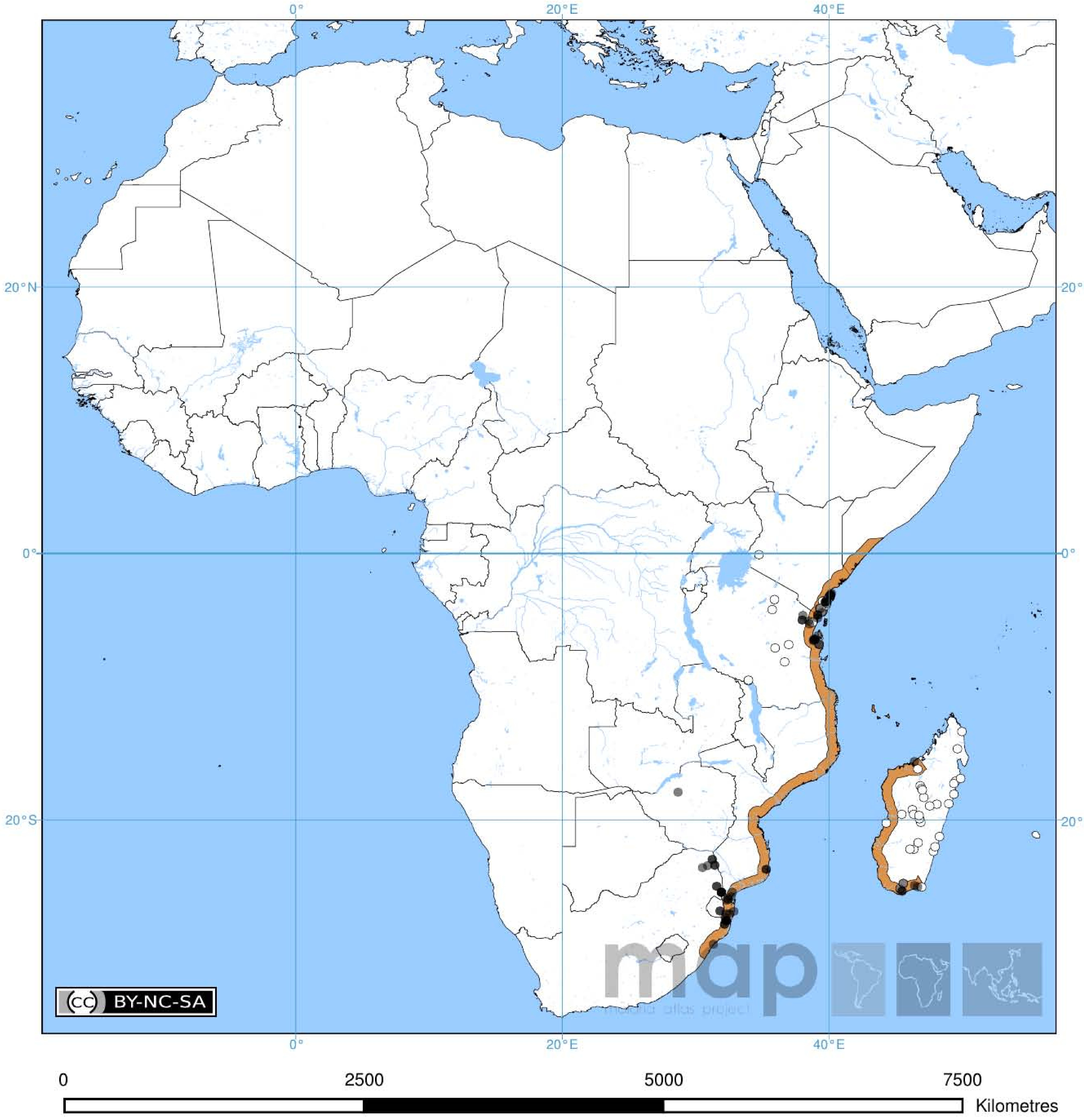

The 136 records for *Anopheles* (*Cellia*) *merus* Dönitz, 1902, were found in 7 countries between 1982 and 2008. There were 73 records of occurrence and 63 records of true absence.

Note: We are particularly keen to augment this map with occurrence records from the coastal distribution of this species in Somalia, Tanzania, Mozambique and Madagascar. Please e-mail map.vector@zoo.ox.ac.uk if you have any useful information to share on the distribution of this species. This is a saltwater larvae species that lives in lagoons and swamps along the coast of East Africa. Occasionally it is found in inland habitats in Tanzania, South Africa and Zimbabwe. The inland points on this map have been checked and confirmed as correct.

Copyright: Licensed to the Malaria Atlas Project (MAP; www.map.ox.ac.uk) under a Creative Commons Attribution 3.0 License (http://creativecommons.org/).

Citation: Hay, S.I. *et al.* (2009). Developing global maps of the dominant *Anopheles* vectors of human malaria. *PLoS Medicine* **6**: in press.

EO range

Present

Absent

Anopheles (*Anopheles*) *messeae* Falleroni, 1926

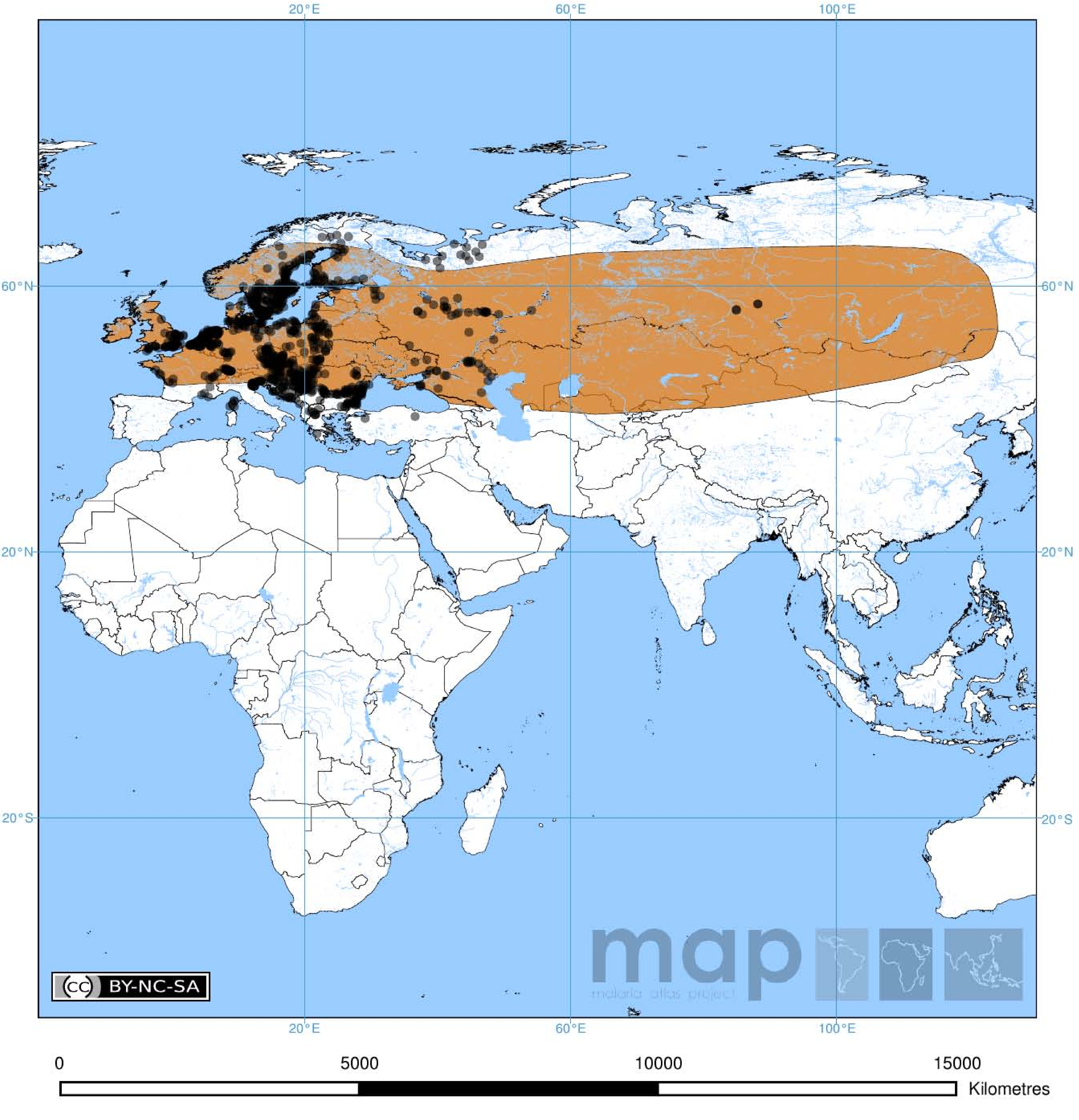

The 915 records for *Anopheles* (*Anopheles*) *messeae* Falleroni, 1926, were found in 31 countries between 1982 and 2006. There were 903 records of occurrence and 12 records of true absence.

Note: We are particularly keen to augment this map with occurrence records throughout the easterly extent of its range in Asia. Please e-mail map.vector@zoo.ox.ac.uk if you have any useful information to share on the distribution of this species.

Copyright: Licensed to the Malaria Atlas Project (MAP; www.map.ox.ac.uk) under a Creative Commons Attribution 3.0 License (http://creativecommons.org/).

Citation: Hay, S.I. *et al.* (2009). Developing global maps of the dominant *Anopheles* vectors of human malaria. *PLoS Medicine* **6**: in press.

EO range

Present

Absent

Anopheles (*Cellia*) *moucheti* Evans, 1925

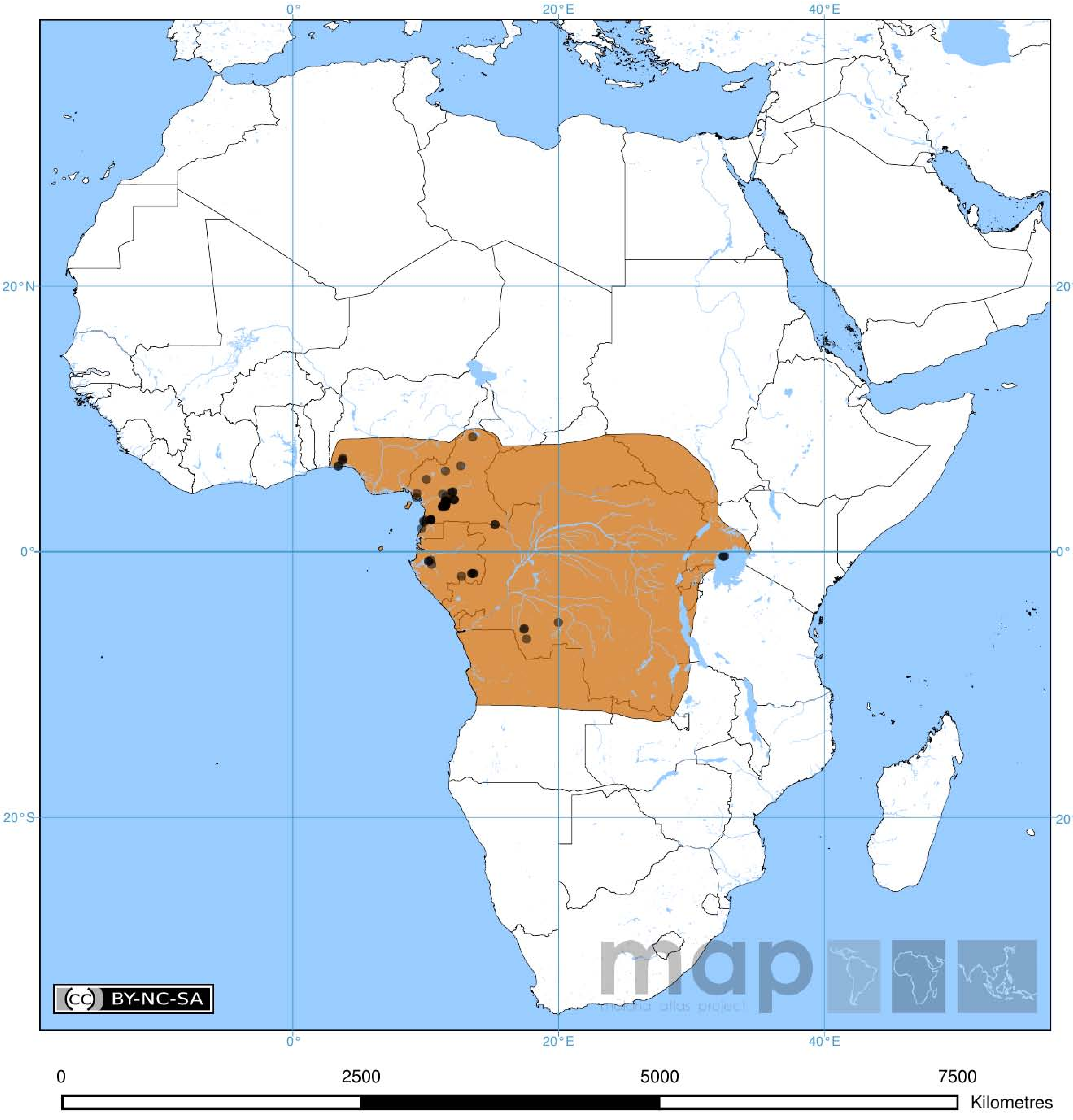

The 69 records for *Anopheles* (*Cellia*) *moucheti* Evans, 1925, were found in 6 countries between 1991 and 2006. There were 69 records of occurrence and 0 records of true absence.

Note: We are particularly keen to augment this map with occurrence records from Nigeria, Central African Republic, Democratic Republic of Congo, Uganda, Rwanda, Burundi, Zambia and Angola. Please e-mail map.vector@zoo.ox.ac.uk if you have any useful information to share on the distribution of this species.

Copyright: Licensed to the Malaria Atlas Project (MAP; www.map.ox.ac.uk) under a Creative Commons Attribution 3.0 License (http://creativecommons.org/).

Citation: Hay, S.I. *et al.* (2009). Developing global maps of the dominant *Anopheles* vectors of human malaria. *PLoS Medicine* **6**: in press.

EO range

Present

Absent

N

Anopheles (*Cellia*) *nili*

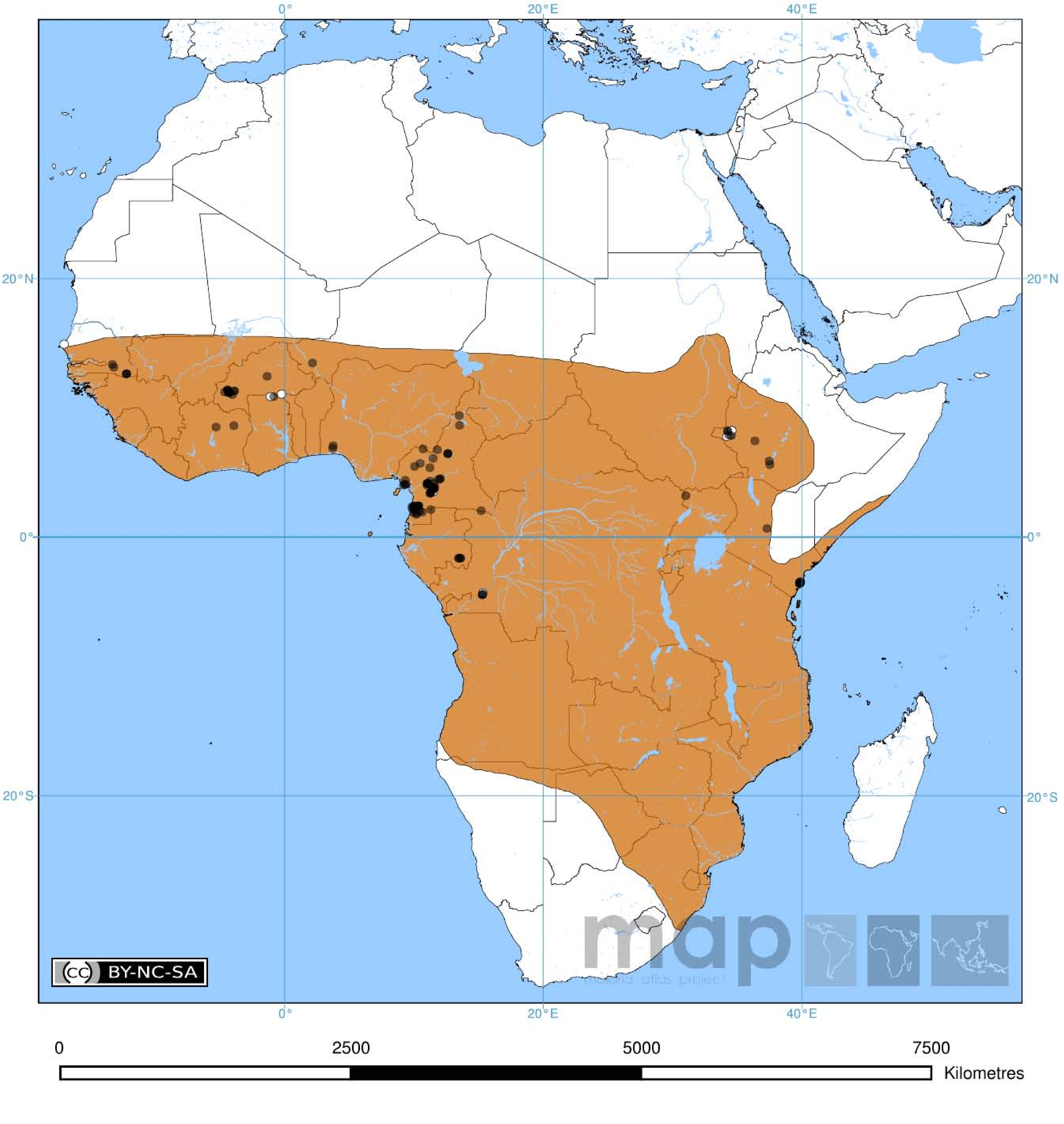

The 114 records for *Anopheles* (*Cellia*) *nili*, were found in 13 countries between 1984 and 2007. There were 105 records of occurrence and 9 records of true absence.

Note: We are particularly keen to augment this map with any occurrence records from the southern half of the distribution in Africa; at present there is a poor prospect of modelling the distribution due to the paucity of information. Please e-mail map.vector@zoo.ox.ac.uk if you have any useful on the distribution of this species compex.

Copyright: Licensed to the Malaria Atlas Project (MAP; www.map.ox.ac.uk) under a Creative Commons Attribution 3.0 License (http://creativecommons.org/).

Citation: Hay, S.I. *et al.* (2009). Developing global maps of the dominant *Anopheles* vectors of human malaria. *PLoS Medicine* **6**: in press.

Anopheles (*Anopheles*) *sacharovi* Favre, 1903

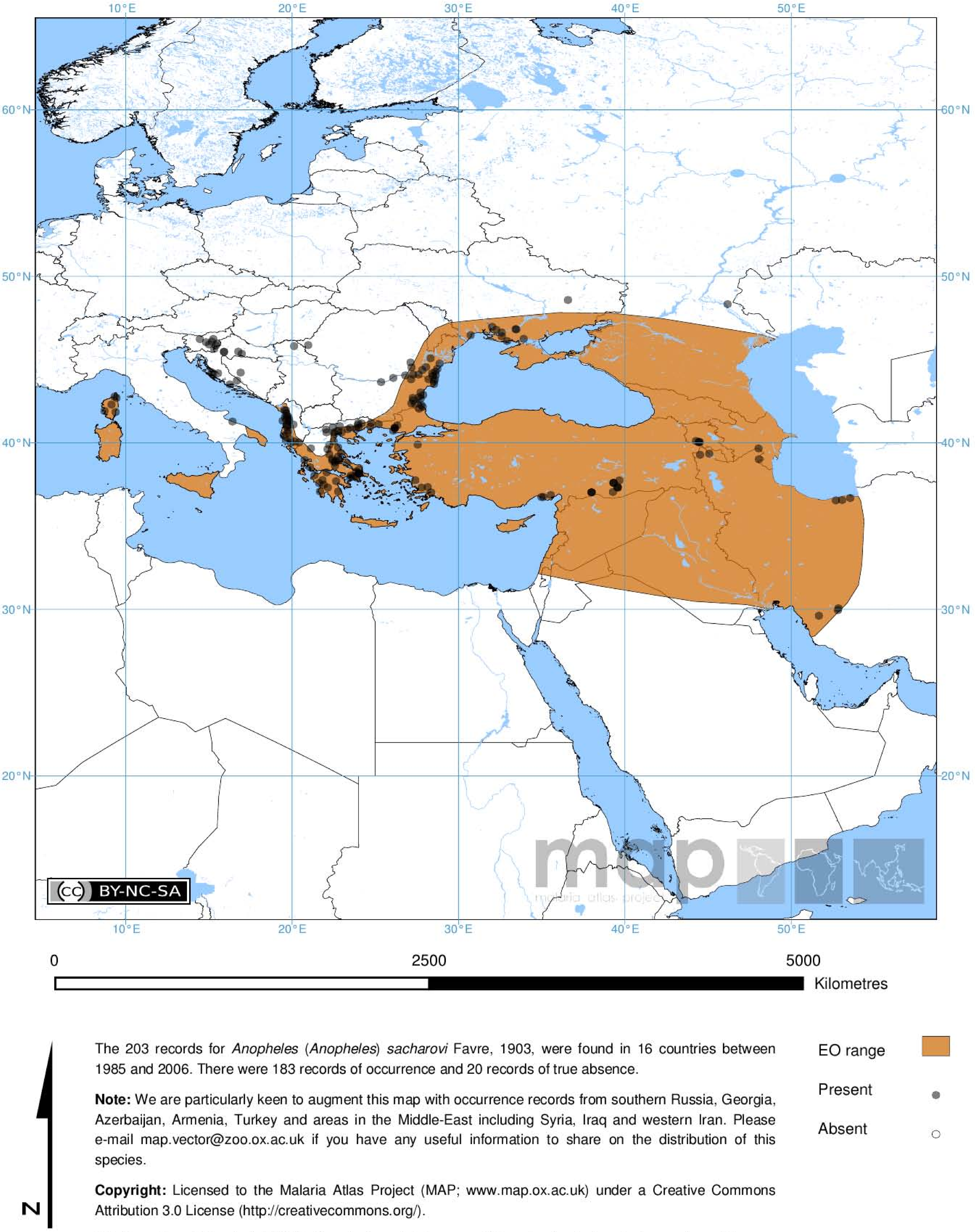

The 203 records for *Anopheles* (*Anopheles*) *sacharovi* Favre, 1903, were found in 16 countries between 1985 and 2006. There were 183 records of occurrence and 20 records of true absence.

Note: We are particularly keen to augment this map with occurrence records from southern Russia, Georgia, Azerbaijan, Armenia, Turkey and areas in the Middle-East including Syria, Iraq and western Iran. Please e-mail map.vector@zoo.ox.ac.uk if you have any useful information to share on the distribution of this species.

Copyright: Licensed to the Malaria Atlas Project (MAP; www.map.ox.ac.uk) under a Creative Commons Attribution 3.0 License (http://creativecommons.org/).

Citation: Hay, S.I. *et al.* (2009). Developing global maps of the dominant *Anopheles* vectors of human malaria. *PLoS Medicine* **6**: in press.

EO range

Present

Absent

Anopheles (*Cellia*) *sergentii* (Theobald, 1907)

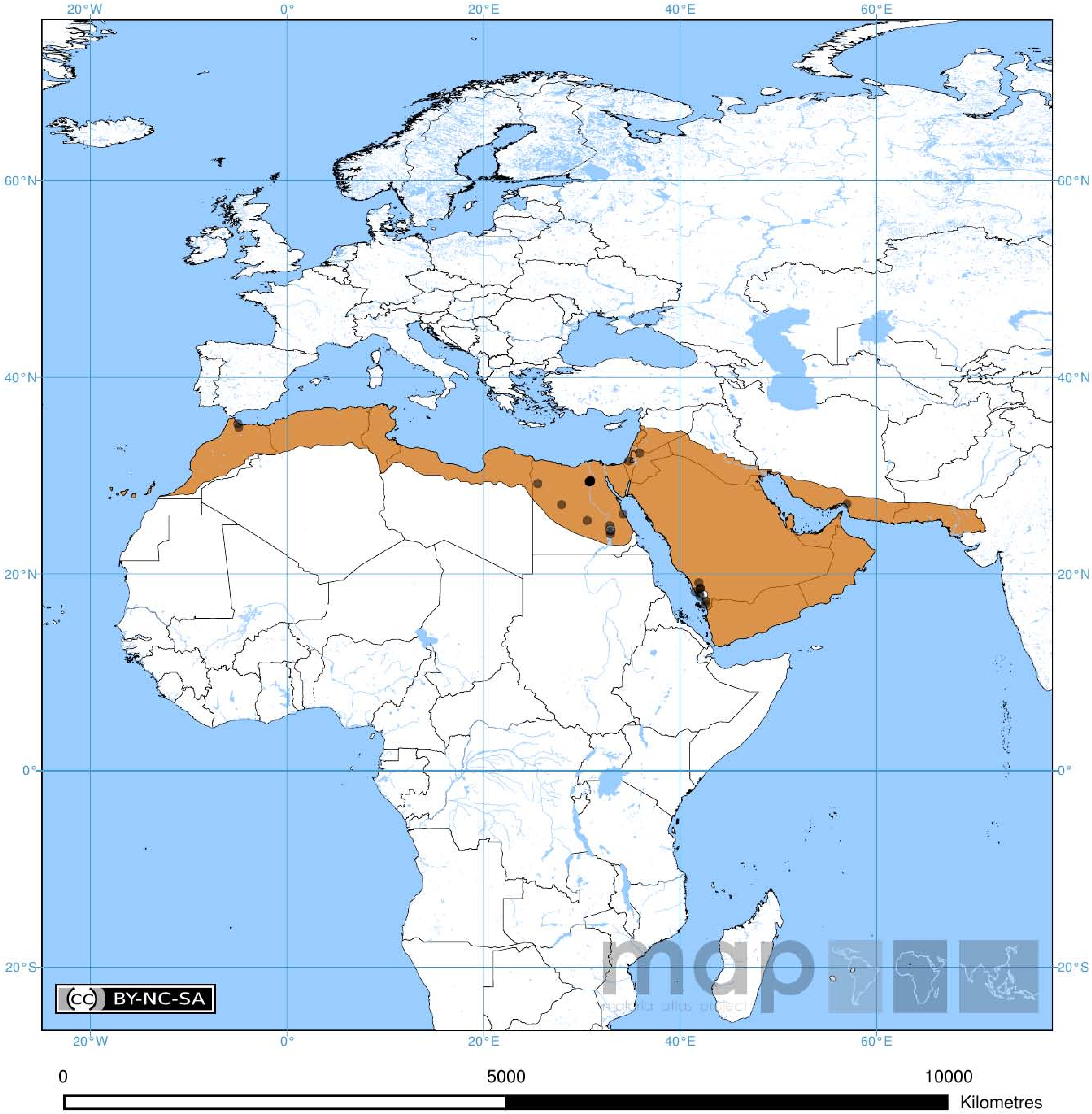

N

The 39 records for *Anopheles* (*Cellia*) *sergentii* (Theobald, 1907), were found in 6 countries between 1982 and 2005. There were 35 records of occurrence and 4 records of true absence.

Note: We are particularly keen to augment this map with occurrence records from the countries of North Africa, Israel, Jordan, Lebanon, Syria, Iraq, Saudi Arabia, the United Arab Emirates, Oman and Yemen, as well as, southern Iran and southern Pakistan. Please e-mail map.vector@zoo.ox.ac.uk if you have any useful information to share on the distribution of this species.

Copyright: Licensed to the Malaria Atlas Project (MAP; www.map.ox.ac.uk) under a Creative Commons Attribution 3.0 License (http://creativecommons.org/).

Citation: Hay, S.I. *et al.* (2009). Developing global maps of the dominant *Anopheles* vectors of human malaria. *PLoS Medicine* **6**: in press.

EO range

Present

Absent

Anopheles (*Cellia*) *superpictus* Grassi, 1899

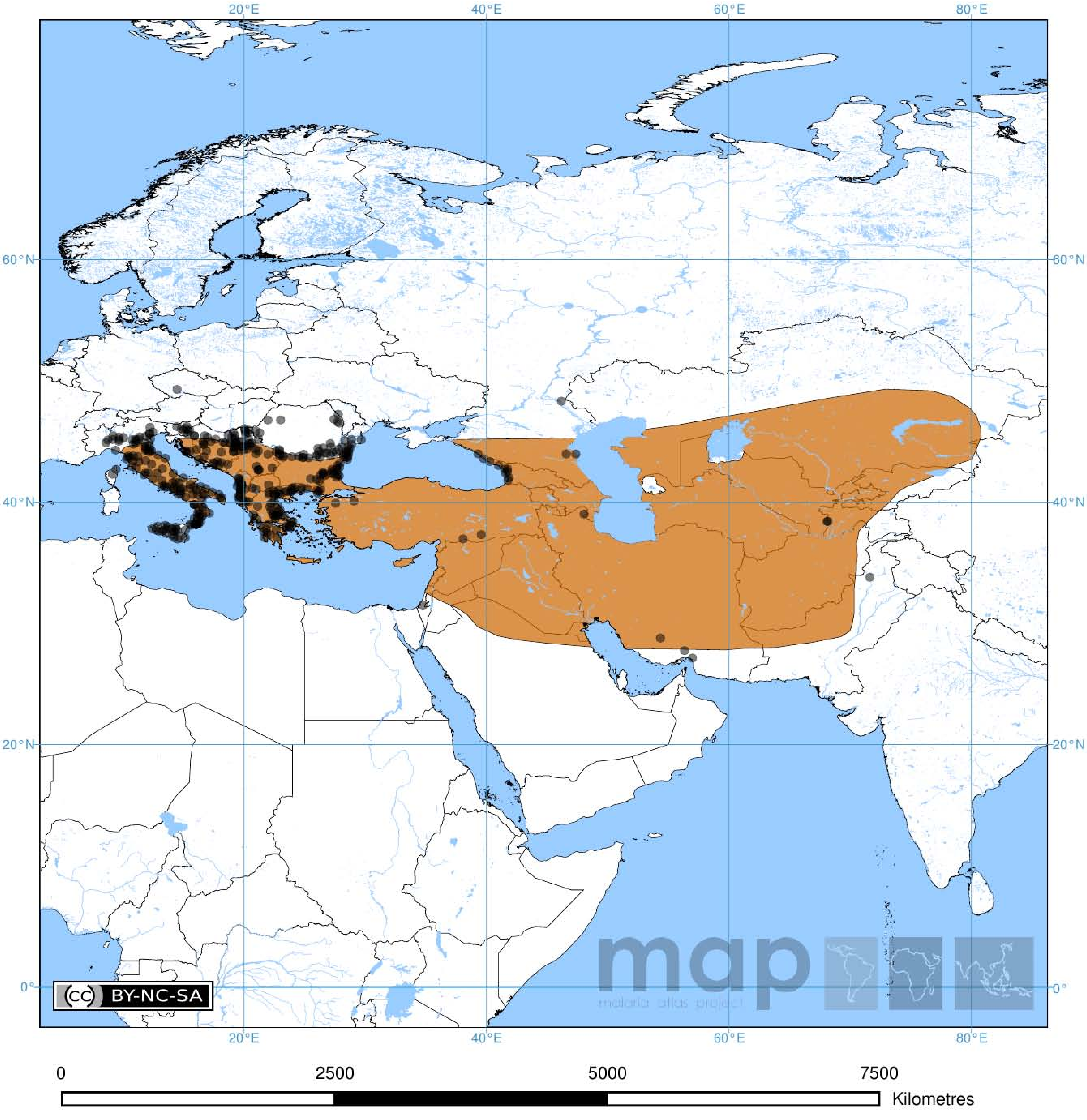

The 394 records for *Anopheles* (*Cellia*) *superpictus* Grassi, 1899, were found in 20 countries between 1984 and 2007. There were 385 records of occurrence and 9 records of true absence.

EO range

Present

Absent

Note: We are particularly keen to augment this map with occurrence records across its distribution in Turkey and the Balkans, the Middle-East, as well as, Turkmenistan, Uzbekistan and Kazakhstan in the north west to Afghanistan in the South. Please e-mail map.vector@zoo.ox.ac.uk if you have any useful information to share on the distribution of this species.

Copyright: Licensed to the Malaria Atlas Project (MAP; www.map.ox.ac.uk) under a Creative Commons Attribution 3.0 License (http://creativecommons.org/).

Citation: Hay, S.I. *et al.* (2009). Developing global maps of the dominant *Anopheles* vectors of human malaria. *PLoS Medicine* **6**: in press.
