# Supplementary material for: Developing Global Maps of the Dominant Anopheles Vectors of Human Malaria
Source: PLoS Med. 2010 Feb 9;7(2):e1000209. doi: 10.1371/journal.pmed.1000209 (PMC2817710; doi:10.1371/journal.pmed.1000209)
Supplement: Text S5 — Maps of expert opinion distribution and species occurrence records for the dominant Anopheles vector species (and species complexes) of human malaria in the Asia-Pacific region. (4.11 MB PDF) [file pmed.1000209.s005.pdf]

Text S5. Maps of expert opinion distribution and species occurrence records for the dominant *Anopheles* vector species (and species complexes) of human malaria in the Asia Pacific region.

The following maps are of the final 19 dominant *Anopheles* vector species (DVS) and species complexes of human malaria in the Asia Pacific region, as defined in the main text and Text S1. Each map is titled with the full scientific name (see Text S1). The maps are then arranged alphabetically by the species component of the full scientific name.

Each map shows an extent most appropriate to display the hypothesised range of each of the species or species complexes. The hypothesised range encompasses expert opinion (EO, in orange) and the species occurrence records collected from the searches of the formally published literature outlined in the main text and Text S2. The EO was digitised from a representative source and in every case substantially modified by; (i) incorporating verified occurrence records outside the EO; (ii) incorporating the advice of the technical advisory group and; (iii) taking into consideration known species-specific habitat requirements as revealed by elevation surfaces [1], satellite imagery [2] and land cover maps [3]. The occurrence records are coded grey for present and white for absent. They are mapped so that presence points overlay absences and multiple presence points in an area appear darker. The latter is important to convey the clustering of survey observations at the regional and continental scale of the maps. The first paragraph of the legend summarizes the number of presence and absence points displayed, along with the date range encompassed by those observations. Major rivers and other inland water bodies are shown in blue and coastlines and national borders in black. Latitude and longitude grids, scale bars and north arrows are presented on all maps along with a creative commons copyright declaration and suggested citation.

These maps are not the definitive product of this project. They are a useful first step in systematically gathering the species distribution information and are released with the EOs to highlight those areas where intensive searching of the informal literature and consultation of local experts is required. A note in each legend highlights areas where we are particularly keen to augment the occurrence records to provide the best “training data” and thus increase chances of accurately mapping the geographical distribution. The maps have been degraded to 200 dpi to restrict their size and thus facilitate rapid download and can be found at higher spatial resolution on the Malaria Atlas Project (MAP) website (http://www.map.ox.ac.uk).

References

1. Slater JA, Garvey G, Johnston C, Haase J, Heady B, et al. (2006) The SRTM data "finishing" process and products. Photogramm Eng Remote Sens 72: 237-247.
2. Scharlemann JPW, Benz D, Hay SI, Purse BV, Tatem AJ, et al. (2008) Global data for ecology and epidemiology: a novel algorithm for temporal Fourier processing MODIS data. PLoS One 3: e1408.
3. Bicheron P, Defourny P, Brockmann C, Vancutsem C, Huc M, et al. (2008) GLOBCOVER: Products Description and Validation Report Toulouse, France MEDIAS-France.

Anopheles (*Cellia*) *aconitus* Dönitz, 1902

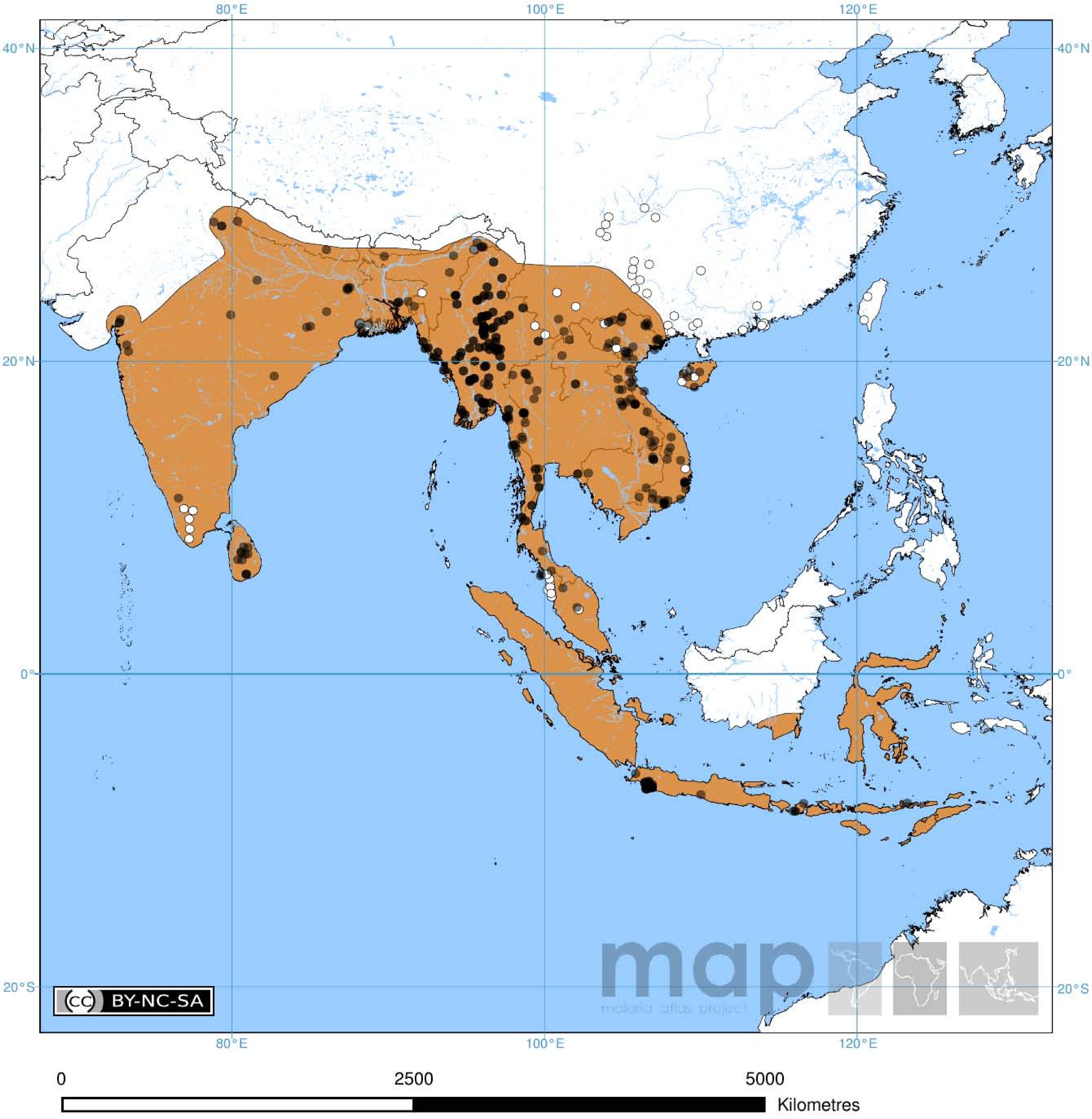

The 490 records for *Anopheles* (*Cellia*) *aconitus* Dönitz, 1902, were found in 13 countries between 1983 and 2007. There were 424 records of occurrence and 66 records of true absence.

Note: We are particularly keen to augment this map with occurrence records from India, central Thailand and Cambodia, as well as Indonesia (Sumatra and Sulawesi). Please e-mail map.vector@zoo.ox.ac.uk if you have any useful information to share on the distribution of this species.

Copyright: Licensed to the Malaria Atlas Project (MAP; www.map.ox.ac.uk) under a Creative Commons Attribution 3.0 License (http://creativecommons.org/).

Citation: Hay, S.I. *et al.* (2009). Developing global maps of the dominant *Anopheles* vectors of human malaria. *PLoS Medicine* **6**: in press.

EO range

Present

Absent

N

Anopheles (*Cellia*) *annularis* van der Wulp, 1884

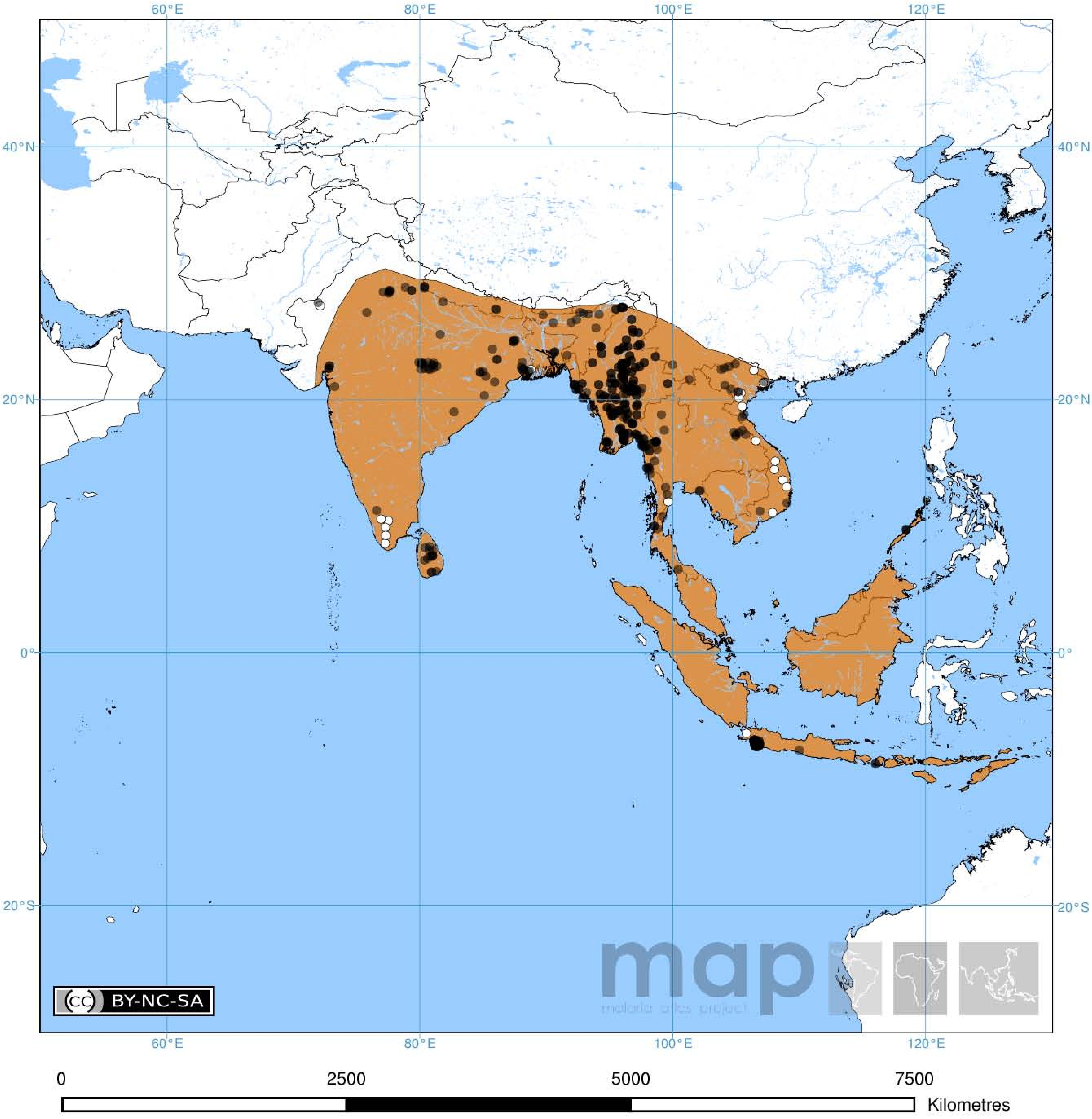

The 521 records for *Anopheles* (*Cellia*) *annularis* van der Wulp, 1884, were found in 11 countries between 1983 and 2006. There were 496 records of occurrence and 25 records of true absence.

Note: We are particularly keen to augment this map with occurrence records from Pakistan, central and southern India, central Thailand, Cambodia, Malaysia and Indonesia. Please e-mail map.vector@zoo.ox.ac.uk if you have any useful information to share on the distribution of this species.

Copyright: Licensed to the Malaria Atlas Project (MAP; www.map.ox.ac.uk) under a Creative Commons Attribution 3.0 License (http://creativecommons.org/).

Citation: Hay, S.I. *et al.* (2009). Developing global maps of the dominant *Anopheles* vectors of human malaria. *PLoS Medicine* **6**: in press.

EO range

Present

Absent

Anopheles (*Cellia*) *balabacensis* Baisas, 1936

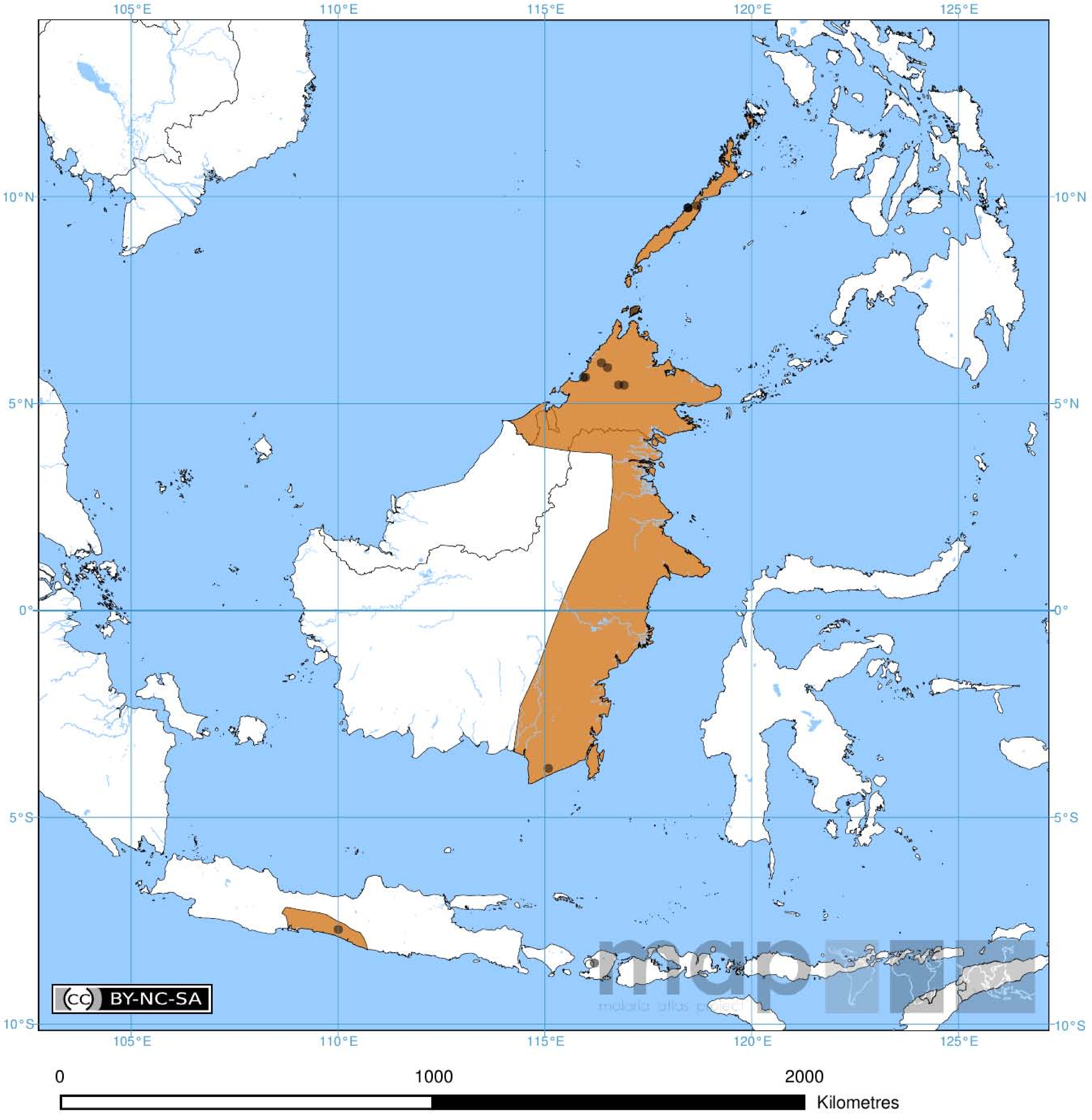

The 16 records for *Anopheles* (*Cellia*) *balabacensis* Baisas, 1936, were found in 3 countries between 1984 and 2003. There were 14 records of occurrence and 2 records of true absence.

EO range

Present

Absent

Note: We are particularly keen to augment this map with occurrence records from Indonesia (particularly Borneo) and Malaysia (Sarawak and Sabah). Please e-mail map.vector@zoo.ox.ac.uk if you have any useful information to share on the distribution of this species.

Copyright: Licensed to the Malaria Atlas Project (MAP; www.map.ox.ac.uk) under a Creative Commons Attribution 3.0 License (http://creativecommons.org/).

Citation: Hay, S.I. *et al.* (2009). Developing global maps of the dominant *Anopheles* vectors of human malaria. *PLoS Medicine* **6**: in press.

Anopheles (*Anopheles*) *barbirostris*

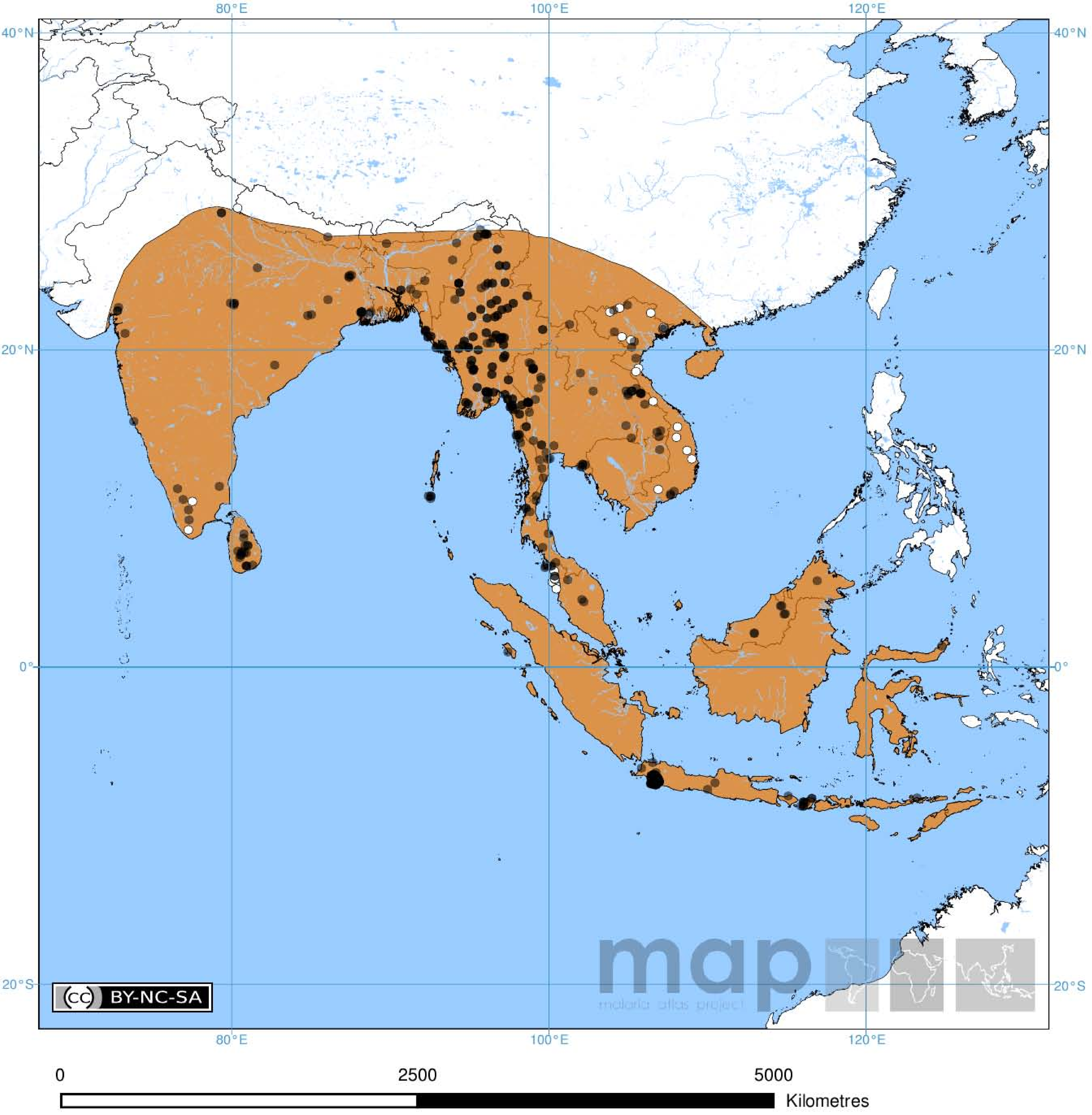

The 902 records for *Anopheles* (*Anopheles*) *barbirostris*, were found in 12 countries between 1983 and 2008. There were 872 records of occurrence and 30 records of true absence.

Note: We are particularly keen to augment this map with occurrence records from central India, Cambodia and Thailand, as well as, Indonesia (particularly Sumatra, Borneo and Sulawesi) and Malaysia (particularly Sarawak and Sabah). Please e-mail map.vector@zoo.ox.ac.uk if you have any useful information to share on the distribution of this species complex.

Copyright: Licensed to the Malaria Atlas Project (MAP; www.map.ox.ac.uk) under a Creative Commons Attribution 3.0 License (http://creativecommons.org/).

Citation: Hay, S.I. *et al.* (2009). Developing global maps of the dominant *Anopheles* vectors of human malaria. *PLoS Medicine* **6**: in press.

EO range

Present

Absent

Anopheles (*Cellia*) *culicifacies*

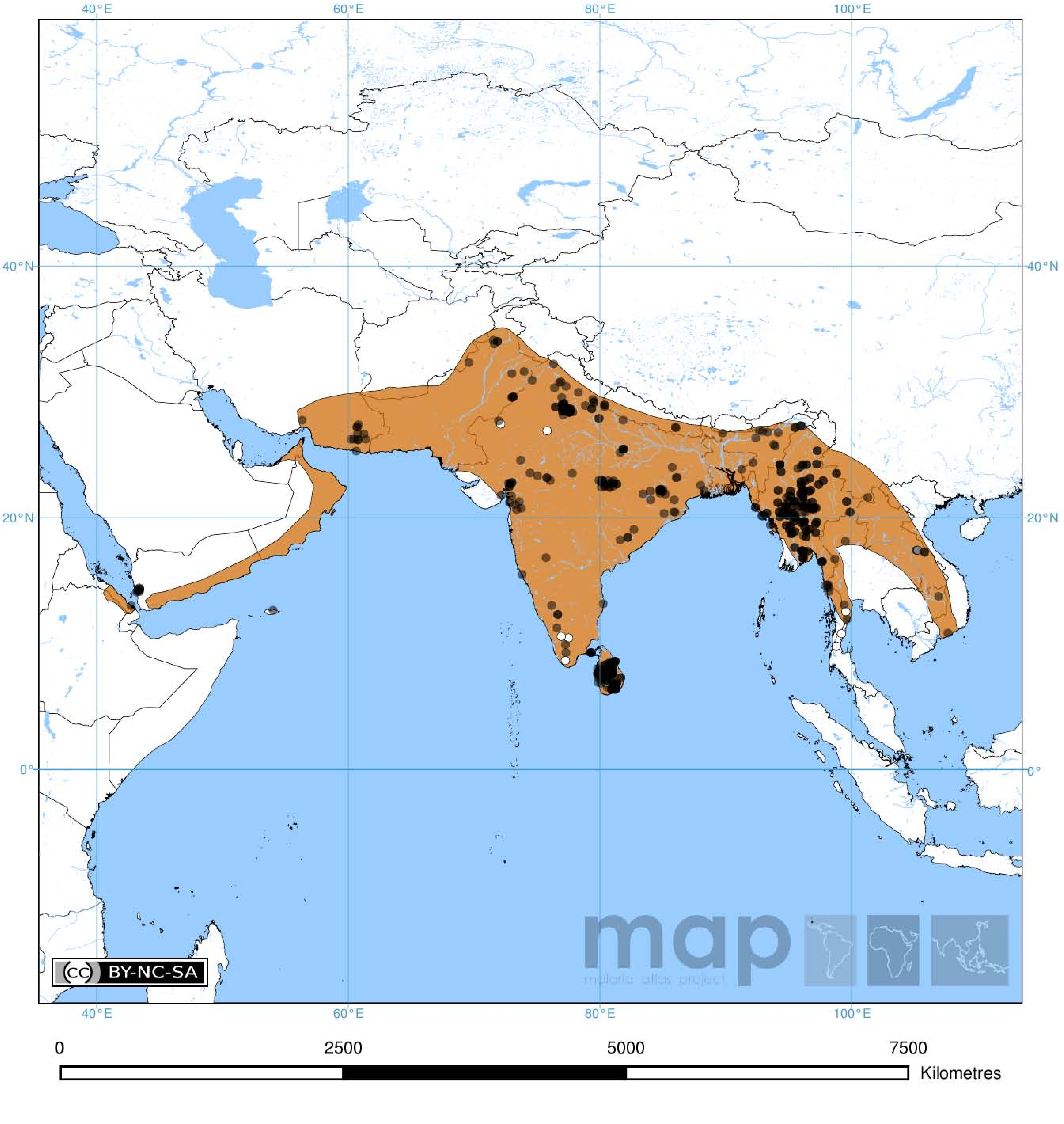

The 564 records for *Anopheles* (*Cellia*) *culicifacies*, were found in 13 countries between 1983 and 2007. There were 550 records of occurrence and 14 records of true absence.

Note: We are particularly keen to augment this map with occurrence records from the western extent of the species range in Yemen, Oman, United Arab Emirates, Iran and Pakistan, as well as records from the easterly extent in Thailand, Laos, Cambodia and Vietnam. Please e-mail map.vector@zoo.ox.ac.uk if you have any useful information to share on the distribution of this species complex. This species complex is to be further stratified with focus on *An. culicifacies* Sp. A, C, D and E.

Copyright: Licensed to the Malaria Atlas Project (MAP; www.map.ox.ac.uk) under a Creative Commons Attribution 3.0 License (http://creativecommons.org/).

Citation: Hay, S.I. *et al.* (2009). Developing global maps of the dominant *Anopheles* vectors of human malaria. *PLoS Medicine* **6**: in press.

EO range

Present

Absent

Anopheles (*Cellia*) *dirus*

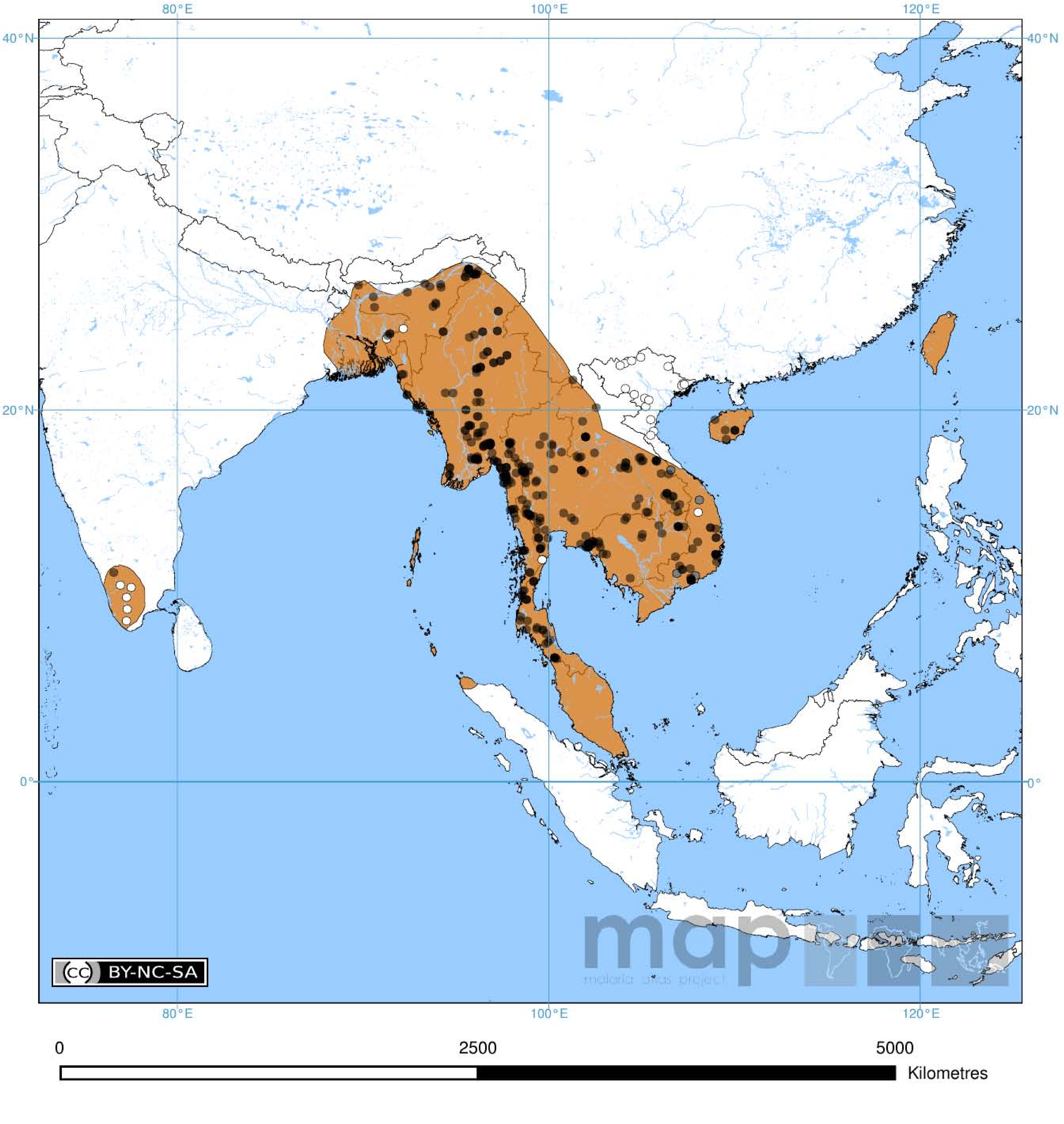

The 403 records for *Anopheles* (*Cellia*) *dirus*, were found in 8 countries between 1983 and 2008. There were 372 records of occurrence and 31 records of true absence.

Note: We are particularly keen to augment this map with occurrence records from Bangladesh and Laos. Please e-mail map.vector@zoo.ox.ac.uk if you have any useful information to share on the distribution of this species complex. This species complex is to be further stratified with focus on *An. dirus* and *An. baimaii*.

Copyright: Licensed to the Malaria Atlas Project (MAP; www.map.ox.ac.uk) under a Creative Commons Attribution 3.0 License (http://creativecommons.org/).

Citation: Hay, S.I. *et al.* (2009). Developing global maps of the dominant *Anopheles* vectors of human malaria. *PLoS Medicine* **6**: in press.

EO range

Present

Absent

Anopheles (*Cellia*) *farauti*

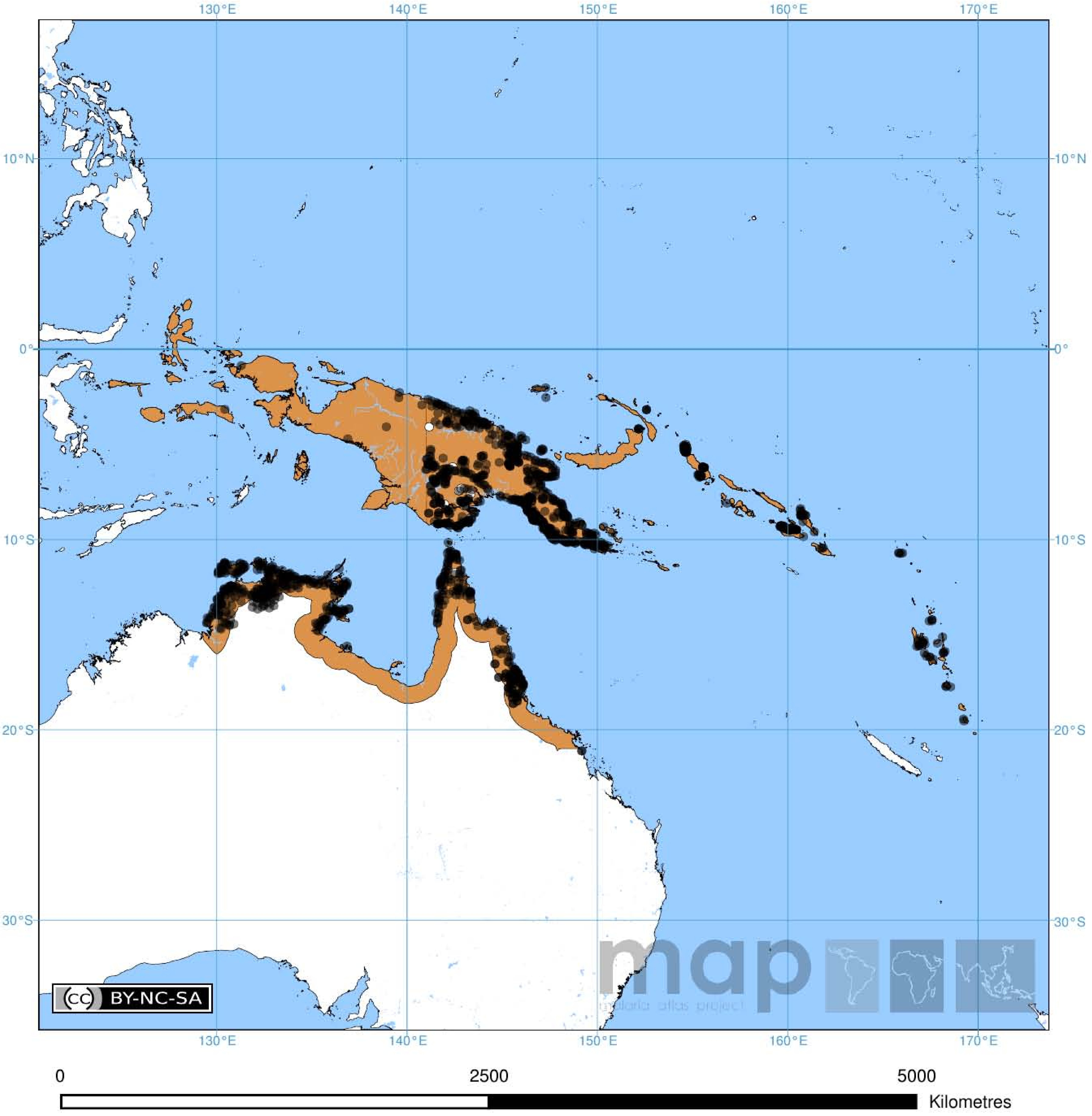

The 1507 records for *Anopheles* (*Cellia*) *farauti*, were found in 5 countries between 1983 and 2007. There were 1465 records of occurrence and 42 records of true absence.

Note: We are particularly keen to augment this map with occurrence records from Indonesian Papua, as well as records from the southerly extent of the range in Australia. Please e-mail map.vector@zoo.ox.ac.uk if you have any useful information to share on the distribution of this species complex.

Copyright: Licensed to the Malaria Atlas Project (MAP; www.map.ox.ac.uk) under a Creative Commons Attribution 3.0 License (http://creativecommons.org/).

Citation: Hay, S.I. *et al.* (2009). Developing global maps of the dominant *Anopheles* vectors of human malaria. *PLoS Medicine* **6**: in press.

EO range

Present

Absent

N

Anopheles (*Cellia*) *flavirostris* (Ludlow, 1914)

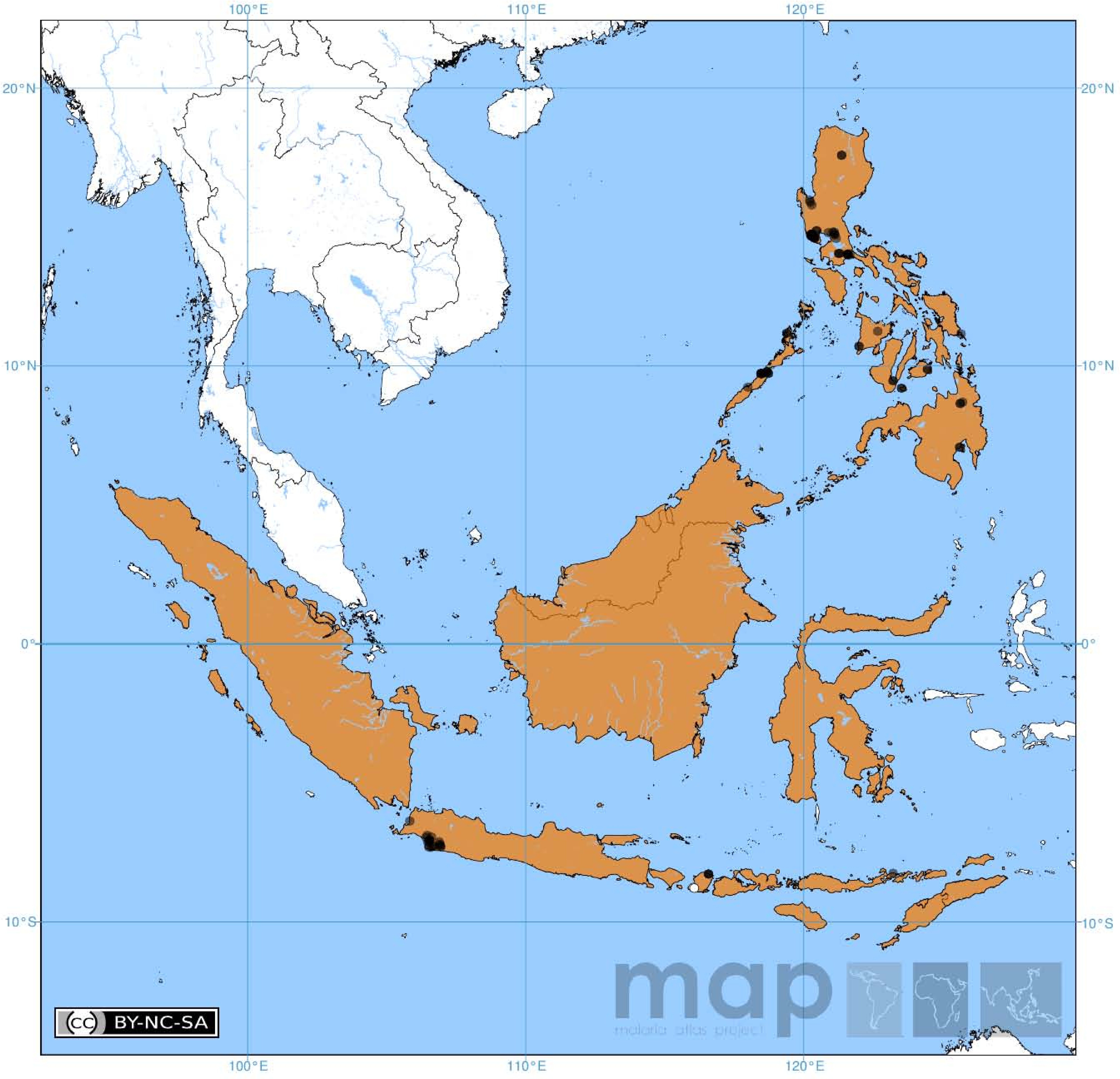

0 1000 2000 3000 Kilometres

N

The 105 records for *Anopheles* (*Cellia*) *flavirostris* (Ludlow, 1914), were found in 2 countries between 1986 and 2005. There were 103 records of occurrence and 2 records of true absence.

Note: We are particularly keen to augment this map with occurrence records from Indonesia (particularly Sumatra, Borneo and Sulawesi) and Malaysia (particularly Sarawak and Sabah). Please e-mail map.vector@zoo.ox.ac.uk if you have any useful information to share on the distribution of this species.

Copyright: Licensed to the Malaria Atlas Project (MAP; www.map.ox.ac.uk) under a Creative Commons Attribution 3.0 License (http://creativecommons.org/).

Citation: Hay, S.I. *et al.* (2009). Developing global maps of the dominant *Anopheles* vectors of human malaria. *PLoS Medicine* **6**: in press.

EO range

Present

Absent

Anopheles (*Cellia*) *fluviatilis*

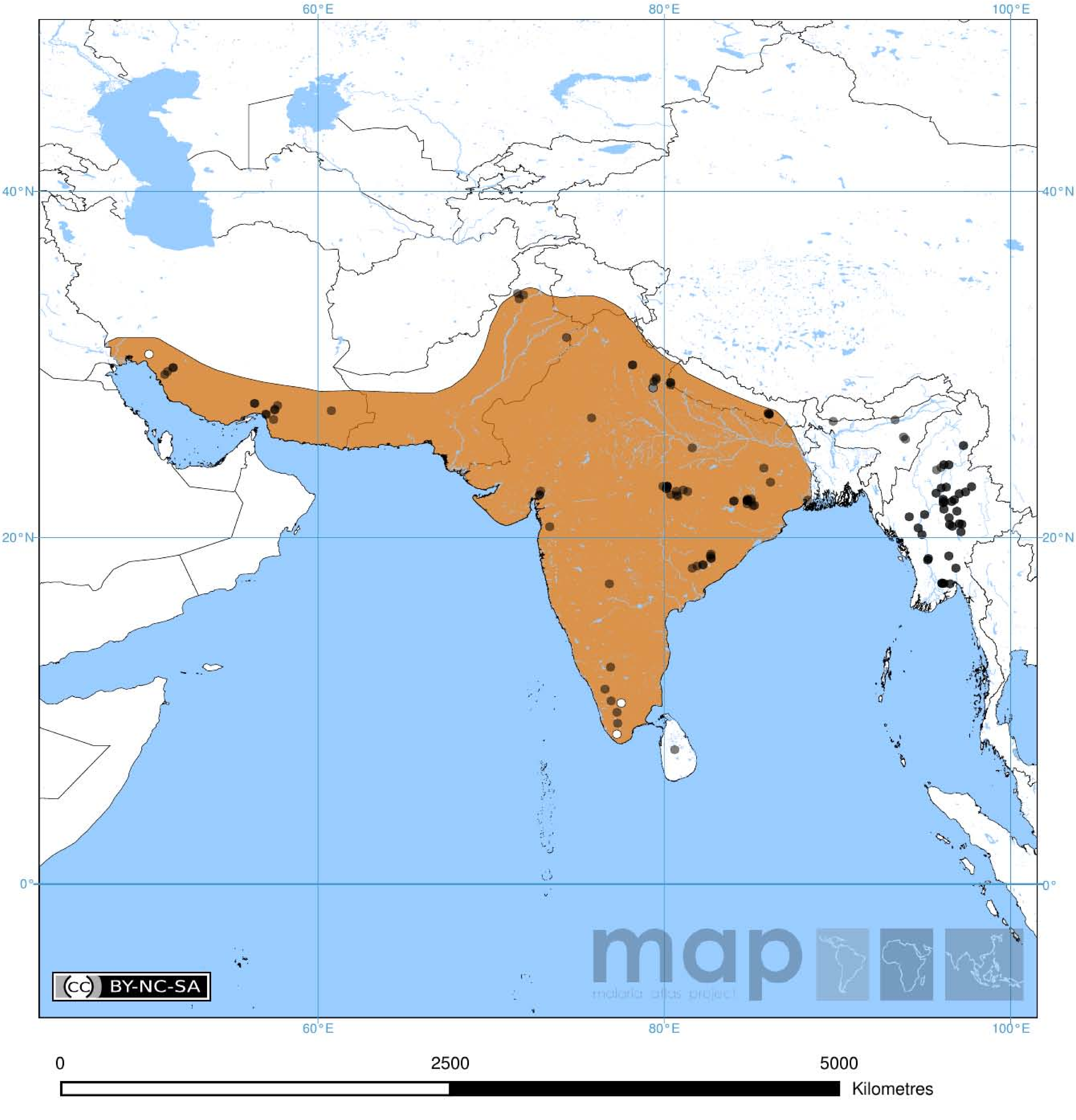

The 158 records for *Anopheles* (*Cellia*) *fluviatilis*, were found in 6 countries between 1983 and 2007. There were 154 records of occurrence and 4 records of true absence.

EO range

Present

Absent

Note: We are particularly keen to augment this map with occurrence records from the western extent of the species range in Iran and Pakistan, as well as from India and Sri Lanka. Please e-mail map.vector@zoo.ox.ac.uk if you have any useful information to share on the distribution of this species complex.

Copyright: Licensed to the Malaria Atlas Project (MAP; www.map.ox.ac.uk) under a Creative Commons Attribution 3.0 License (http://creativecommons.org/).

Citation: Hay, S.I. *et al.* (2009). Developing global maps of the dominant *Anopheles* vectors of human malaria. *PLoS Medicine* **6**: in press.

Anopheles (*Cellia*) *koliensis* Owen, 1945

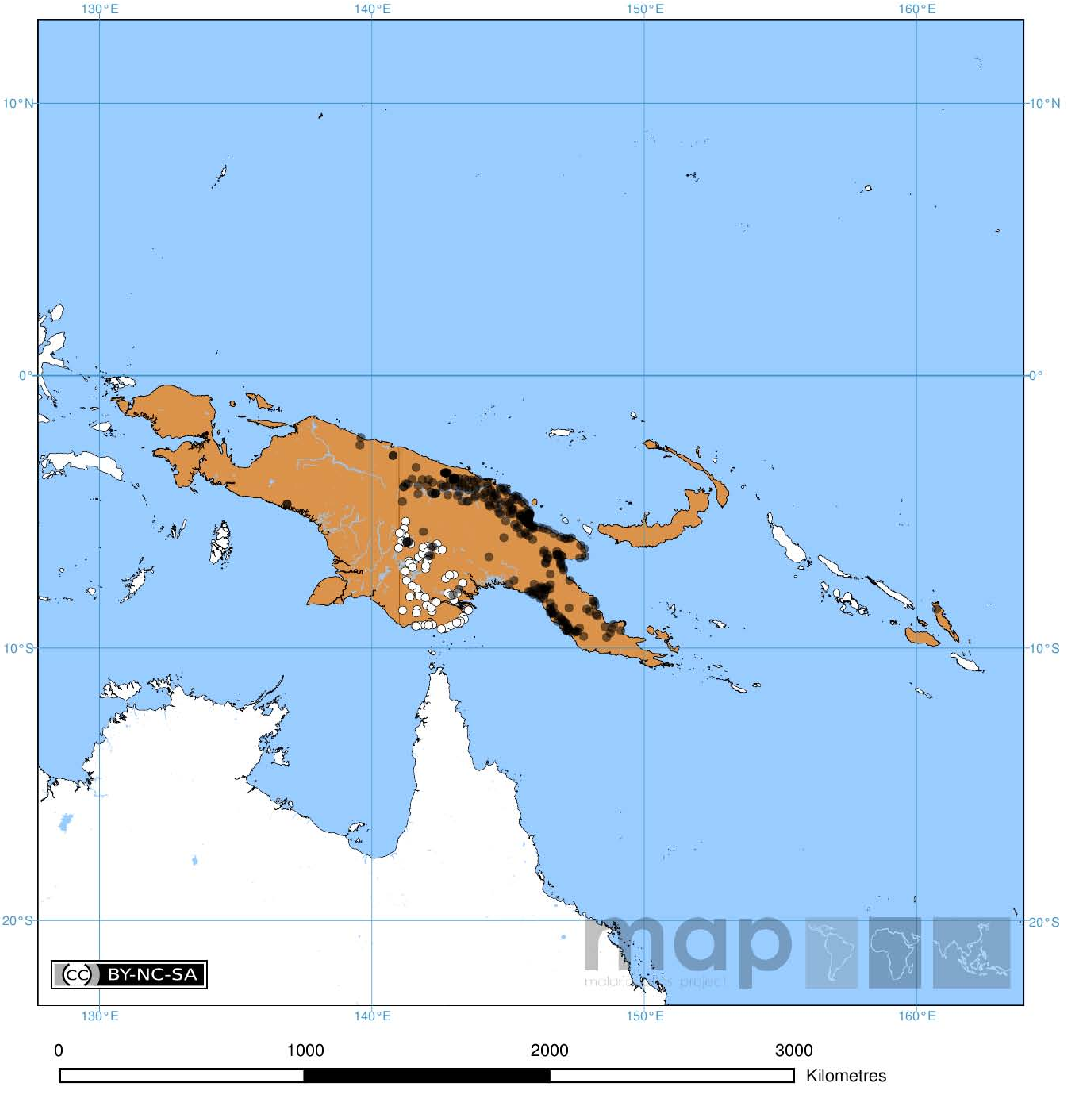

The 393 records for *Anopheles* (*Cellia*) *koliensis* Owen, 1945, were found in 3 countries between 1983 and 1999. There were 325 records of occurrence and 68 records of true absence.

Note: We are particularly keen to augment this map with occurrence records from the Indonesian side of Papua, as well as records from New Britain and the Island of the Solomon Sea. Please e-mail map.vector@zoo.ox.ac.uk if you have any useful information to share on the distribution of this species.

Copyright: Licensed to the Malaria Atlas Project (MAP; www.map.ox.ac.uk) under a Creative Commons Attribution 3.0 License (http://creativecommons.org/).

Citation: Hay, S.I. *et al.* (2009). Developing global maps of the dominant *Anopheles* vectors of human malaria. *PLoS Medicine* **6**: in press.

EO range

Present

Absent

Anopheles (*Anopheles*) *lesteri* Baisas & Hu, 1936

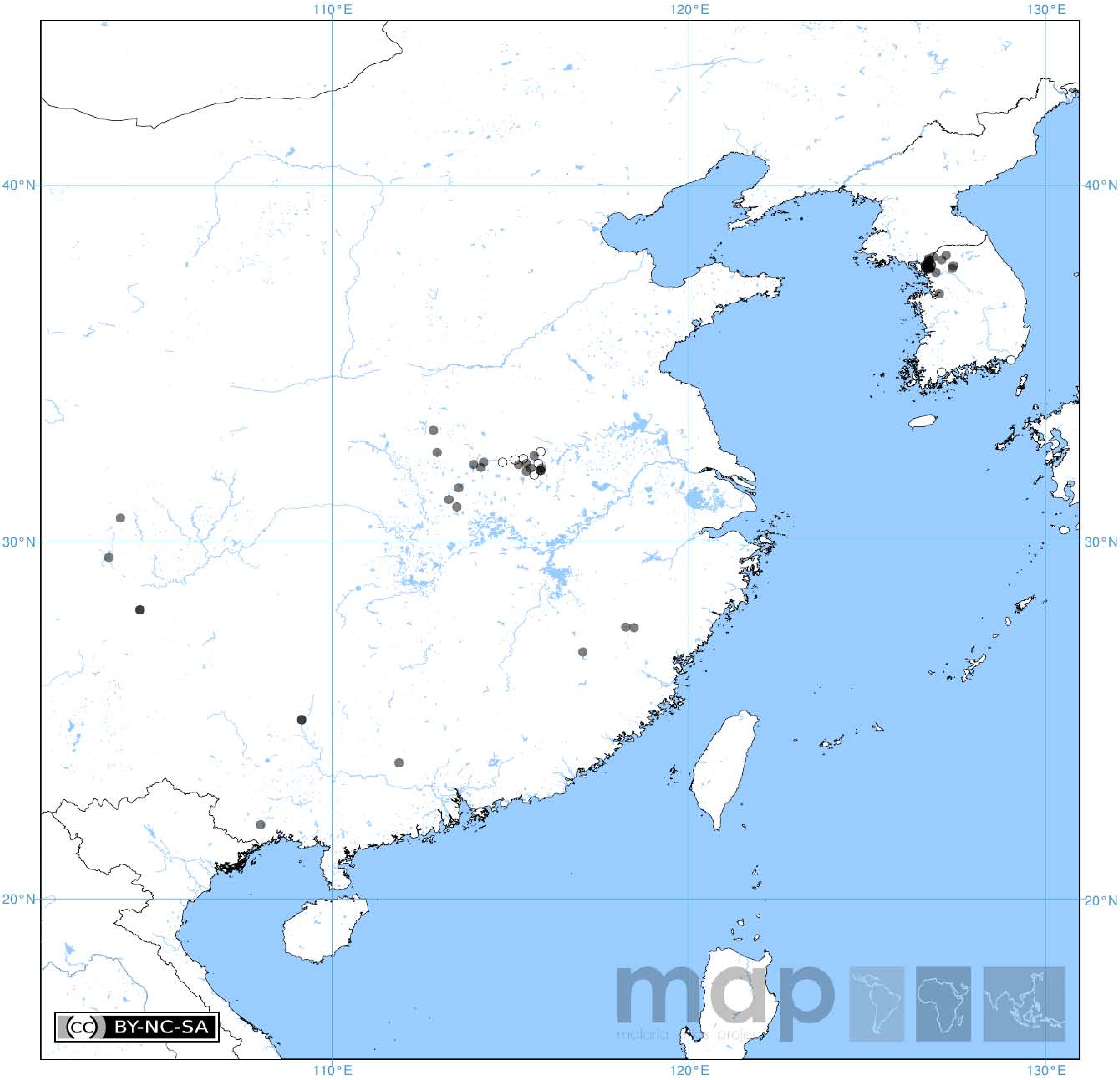

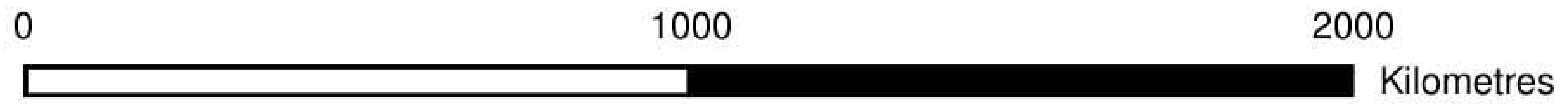

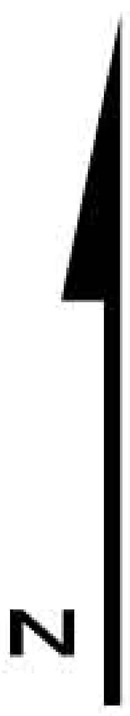

The 56 records for *Anopheles* (*Anopheles*) *lesteri* Baisas & Hu, 1936, were found in 3 countries between 1983 and 2005. There were 47 records of occurrence and 9 records of true absence.

No EO available

Present

Absent

Note: We are particularly keen to augment this map with any occurrence records and solicit advice on its expert opinion distribution. Please e-mail map.vector@zoo.ox.ac.uk if you have any useful information to share on the distribution of this species.

Copyright: Licensed to the Malaria Atlas Project (MAP; www.map.ox.ac.uk) under a Creative Commons Attribution 3.0 License (http://creativecommons.org/).

Citation: Hay, S.I. *et al.* (2009). Developing global maps of the dominant *Anopheles* vectors of human malaria. *PLoS Medicine* **6**: in press.

Anopheles (*Cellia*) *leucosphyrus*

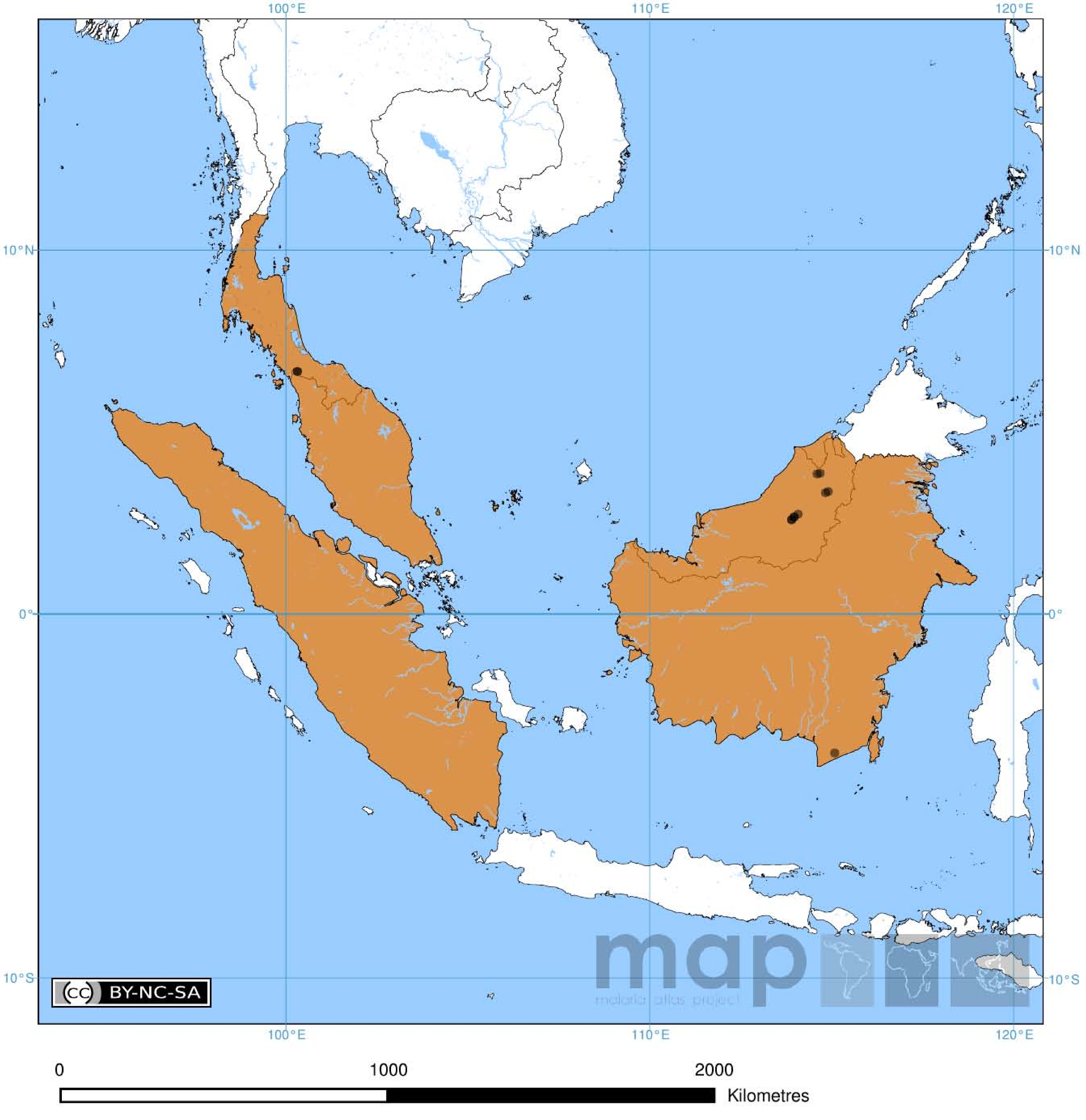

The 12 records for *Anopheles* (*Cellia*) *leucosphyrus*, were found in 3 countries between 1986 and 1994. There were 12 records of occurrence and 0 records of true absence.

EO range

Present

Absent

Note: We are particularly keen to augment this map with any occurrence records from Thailand, Malaysia and Indonesia; at present there is no prospect of modelling the distribution of this species due to the paucity of distribution information. Please e-mail map.vector@zoo.ox.ac.uk if you have any useful information to share on the distribution of this species.

Copyright: Licensed to the Malaria Atlas Project (MAP; www.map.ox.ac.uk) under a Creative Commons Attribution 3.0 License (http://creativecommons.org/).

Citation: Hay, S.I. *et al.* (2009). Developing global maps of the dominant *Anopheles* vectors of human malaria. *PLoS Medicine* **6**: in press.

Anopheles (*Cellia*) *maculatus* Group

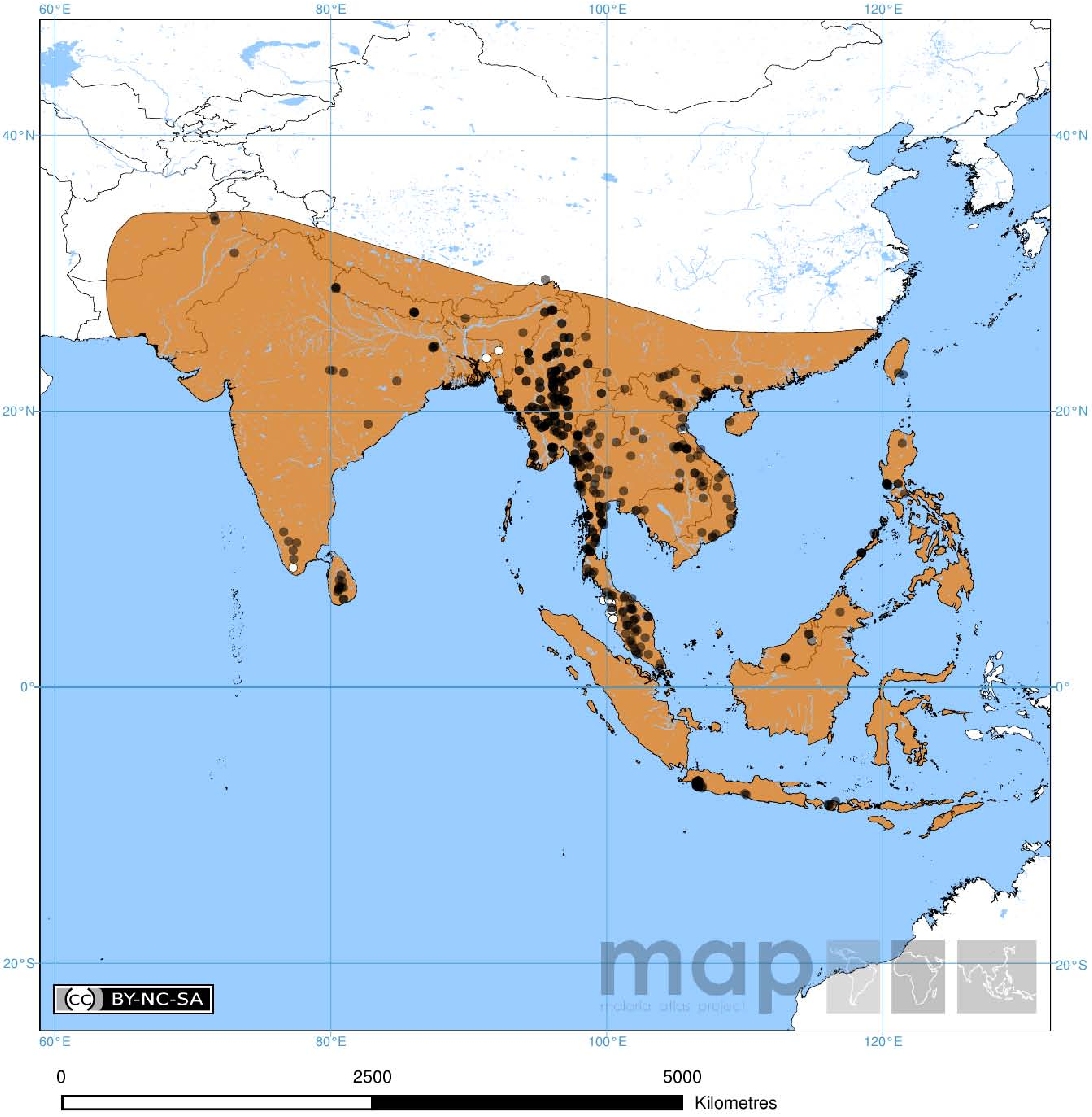

The 490 records for *Anopheles* (*Cellia*) *maculatus* Group, were found in 15 countries between 1983 and 2008. There were 471 records of occurrence and 19 records of true absence.

Note: We are particularly keen to augment this map with occurrence records from Pakistan, India and China, as well as, Indonesia (Sumatra, Borneo and Sulawesi) and Malaysia (Sarawak and Sabah). Please e-mail map.vector@zoo.ox.ac.uk if you have any useful information to share on the distribution of this species group.

Copyright: Licensed to the Malaria Atlas Project (MAP; www.map.ox.ac.uk) under a Creative Commons Attribution 3.0 License (http://creativecommons.org/).

Citation: Hay, S.I. *et al.* (2009). Developing global maps of the dominant *Anopheles* vectors of human malaria. *PLoS Medicine* **6**: in press.

N

EO range

Present

Absent

Anopheles (*Cellia*) *minimus*

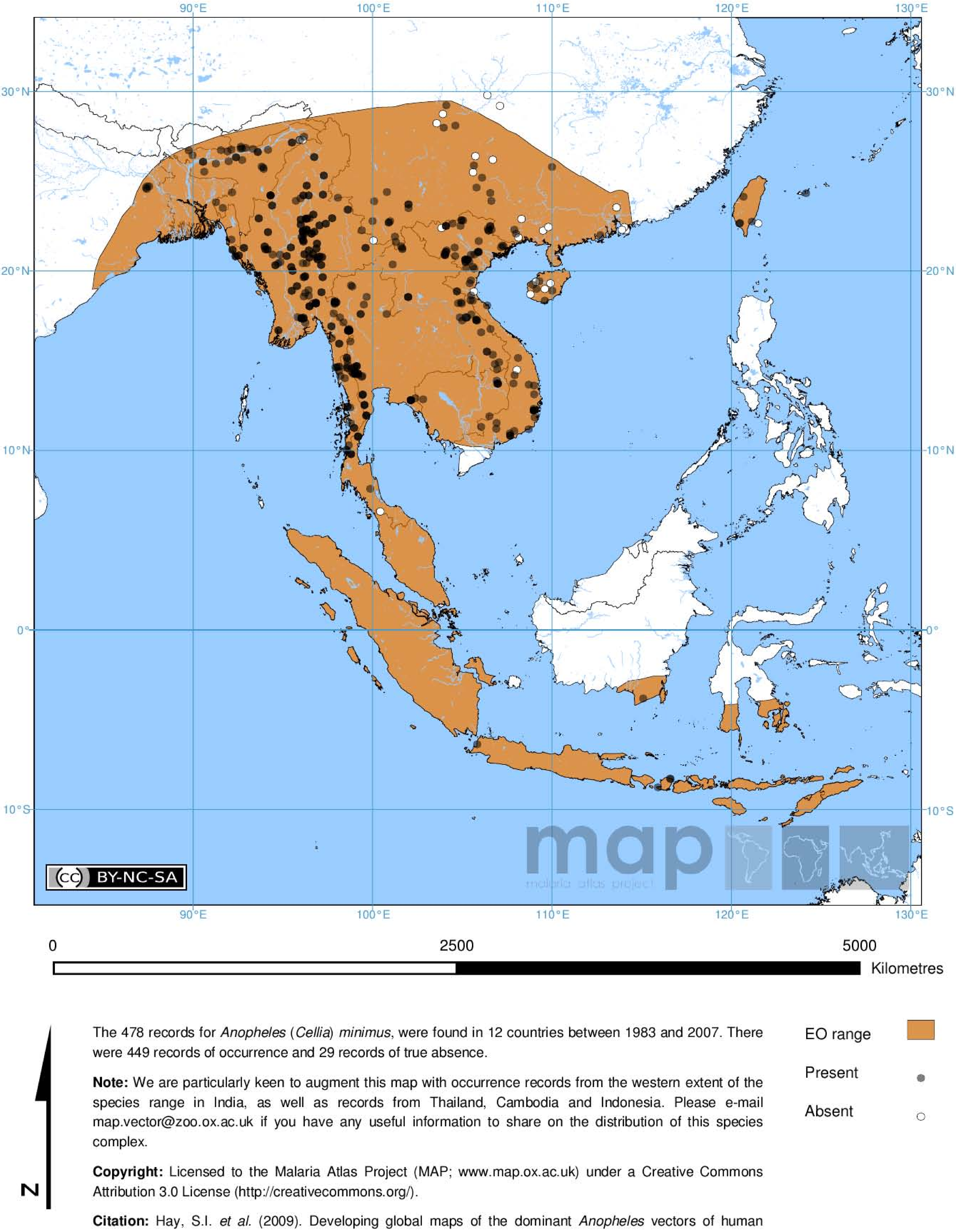

EO range

Present

Absent

The 478 records for *Anopheles* (*Cellia*) *minimus*, were found in 12 countries between 1983 and 2007. There were 449 records of occurrence and 29 records of true absence.

Note: We are particularly keen to augment this map with occurrence records from the western extent of the species range in India, as well as records from Thailand, Cambodia and Indonesia. Please e-mail map.vector@zoo.ox.ac.uk if you have any useful information to share on the distribution of this species complex.

Copyright: Licensed to the Malaria Atlas Project (MAP; www.map.ox.ac.uk) under a Creative Commons Attribution 3.0 License (http://creativecommons.org/).

Citation: Hay, S.I. *et al.* (2009). Developing global maps of the dominant *Anopheles* vectors of human malaria. *PLoS Medicine* **6**: in press.

Anopheles (*Cellia*) *punctulatus*

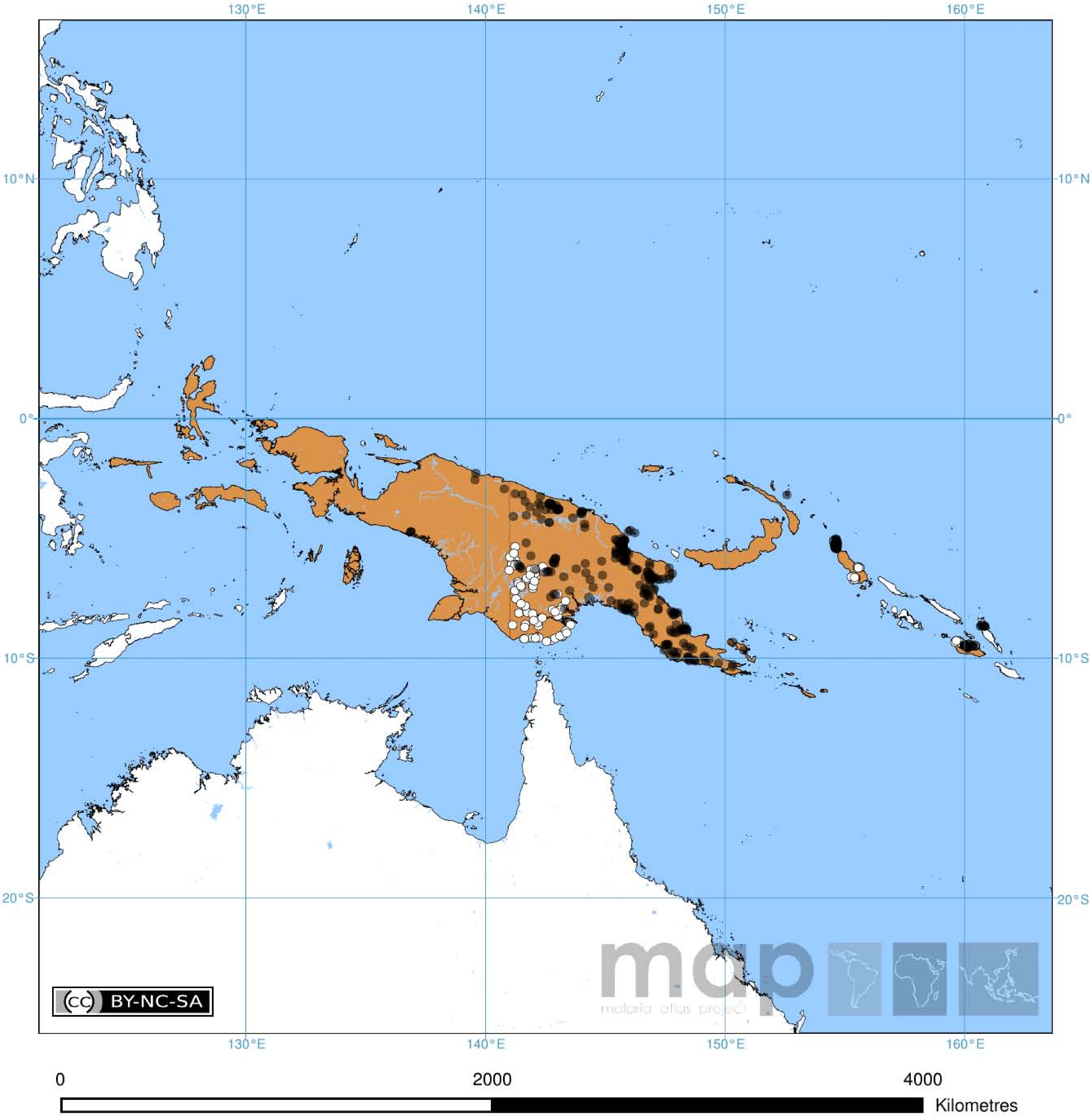

The 532 records for *Anopheles* (*Cellia*) *punctulatus*, were found in 3 countries between 1983 and 2001. There were 379 records of occurrence and 153 records of true absence.

Note: We are particularly keen to augment this map with occurrence records from western Indonesia, including Papua and the Moluccas, as well as in its eastern extent in Papua New Guinea, specifically New Britain. Please e-mail map.vector@zoo.ox.ac.uk if you have any useful information to share on the distribution of this species complex.

Copyright: Licensed to the Malaria Atlas Project (MAP; www.map.ox.ac.uk) under a Creative Commons Attribution 3.0 License (http://creativecommons.org/).

Citation: Hay, S.I. *et al.* (2009). Developing global maps of the dominant *Anopheles* vectors of human malaria. *PLoS Medicine* **6**: in press.

EO range

Present

Absent

N

Anopheles (*Anopheles*) *sinensis*

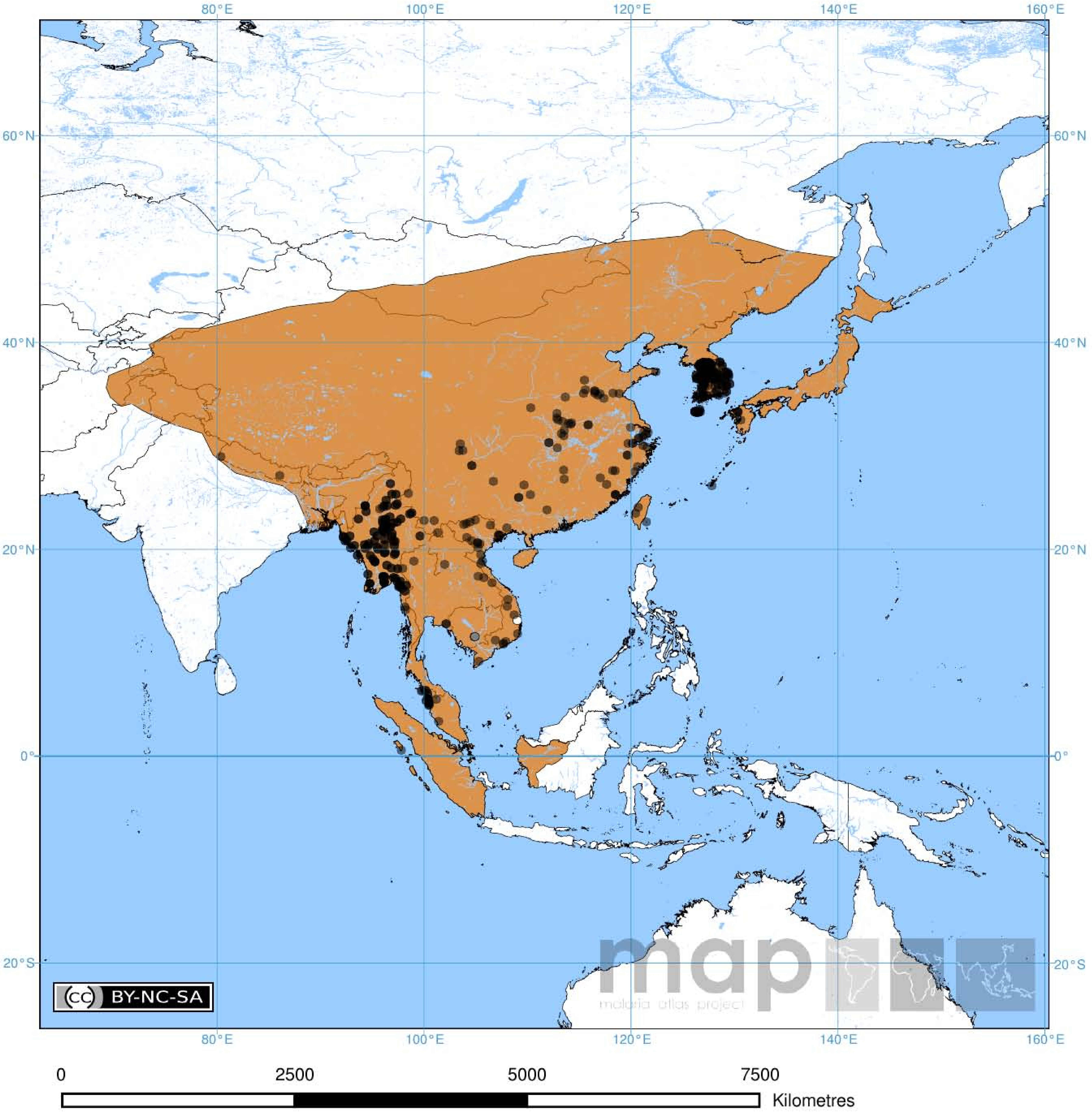

The 573 records for *Anopheles* (*Anopheles*) *sinensis*, were found in 13 countries between 1981 and 2006. There were 568 records of occurrence and 5 records of true absence.

Note: We are particularly keen to augment this map with occurrence records from its northern extent in China, Mongolia, North Korea and Japan, as well as from Pakistan, India and Nepal to the west and Thailand, Cambodia and Indonesian Sumatra to the south. Please e-mail map.vector@zoo.ox.ac.uk if you have any useful information to share on the distribution of this species complex.

Copyright: Licensed to the Malaria Atlas Project (MAP; www.map.ox.ac.uk) under a Creative Commons Attribution 3.0 License (http://creativecommons.org/).

Citation: Hay, S.I. *et al.* (2009). Developing global maps of the dominant *Anopheles* vectors of human malaria. *PLoS Medicine* **6**: in press.

EO range

Present

Absent

N

Anopheles (*Cellia*) *stephensi* Liston, 1901

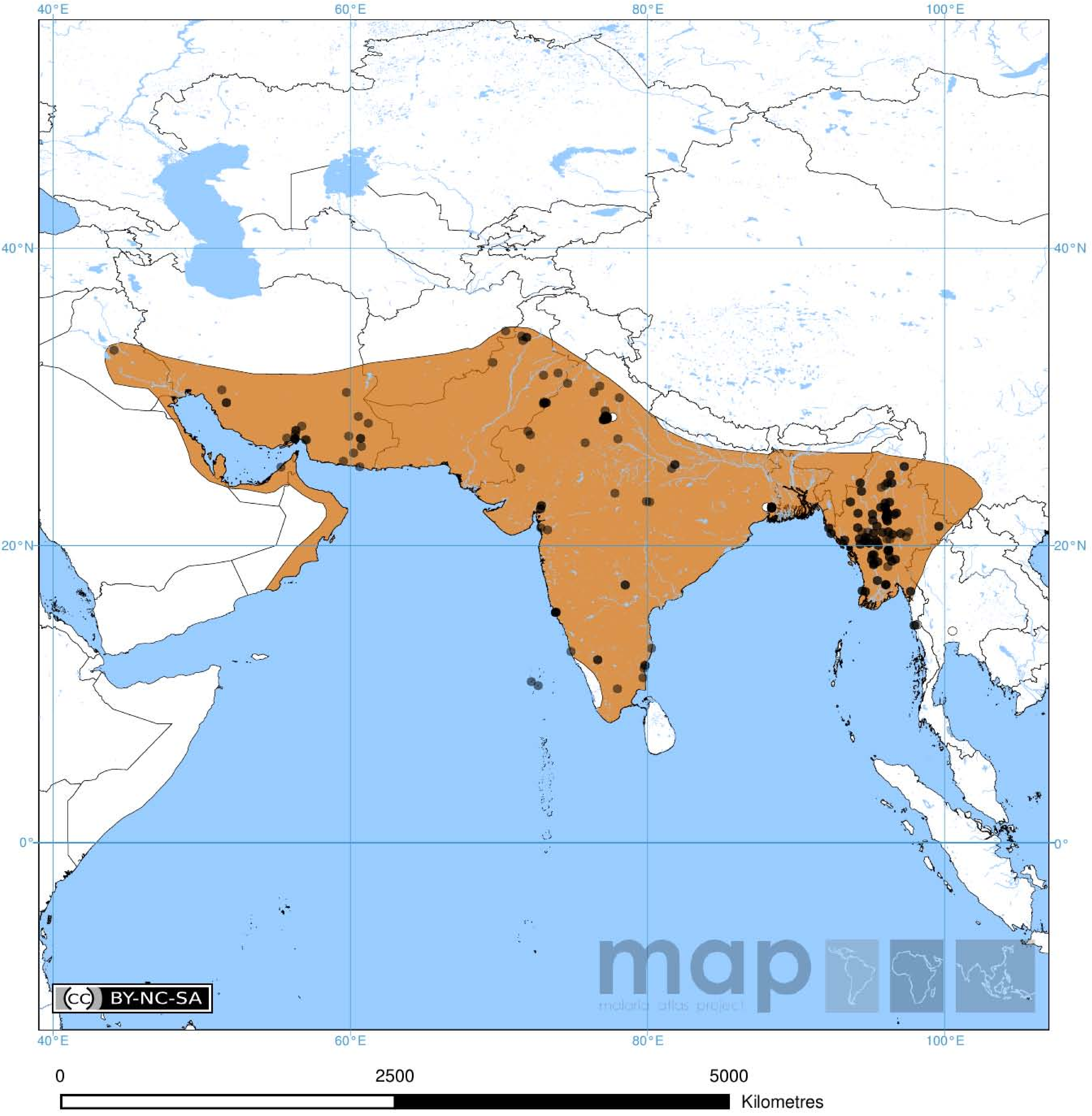

The 265 records for *Anopheles* (*Cellia*) *stephensi* Liston, 1901, were found in 8 countries between 1984 and 2006. There were 261 records of occurrence and 4 records of true absence.

Note: We are particularly keen to augment this map with occurrence records from its westerly extent in Kuwait, Saudi Arabia, United Arab Emirates and Oman, as well as Pakistan, southern Afghanistan and central and western areas of India, Bangladesh and southern China (Yunnan). Please e-mail map.vector@zoo.ox.ac.uk if you have any useful information to share on the distribution of this species.

Copyright: Licensed to the Malaria Atlas Project (MAP; www.map.ox.ac.uk) under a Creative Commons Attribution 3.0 License (http://creativecommons.org/).

Citation: Hay, S.I. *et al.* (2009). Developing global maps of the dominant *Anopheles* vectors of human malaria. *PLoS Medicine* **6**: in press.

EO range

Present

Absent

N

Anopheles (*Cellia*) *subpictus*

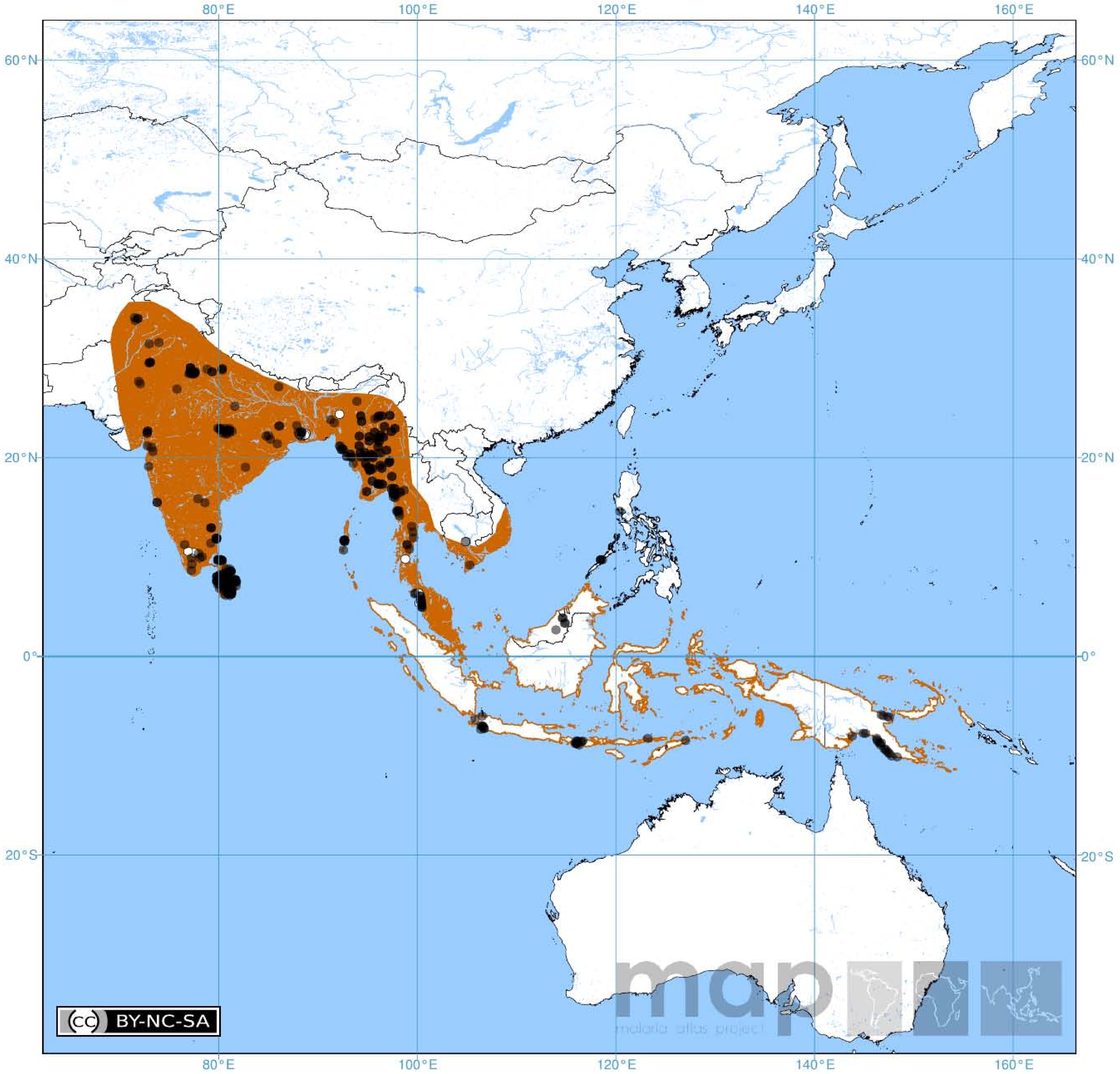

0 5000 10000
Kilometres

N

The 430 records for *Anopheles* (*Cellia*) *subpictus*, were found in 13 countries between 1983 and 2006. There were 410 records of occurrence and 20 records of true absence.

Note: We are particularly keen to augment this map with occurrence records from India (Maharashtra, Madhya Pradesh, Karnataka), as well as Indonesia (Sumatra and Sulawesi, Kalimantan and Papua), Penisular Malaysia and Sabah. Please e-mail map.vector@zoo.ox.ac.uk if you have any useful information to share on the distribution of this species complex.

Copyright: Licensed to the Malaria Atlas Project (MAP; www.map.ox.ac.uk) under a Creative Commons Attribution 3.0 License (http://creativecommons.org/).

Citation: Hay, S.I. *et al.* (2009). Developing global maps of the dominant *Anopheles* vectors of human malaria. *PLoS Medicine* **6**: in press.

EO range

Present

Absent

Anopheles (*Cellia*) *sundaicus*

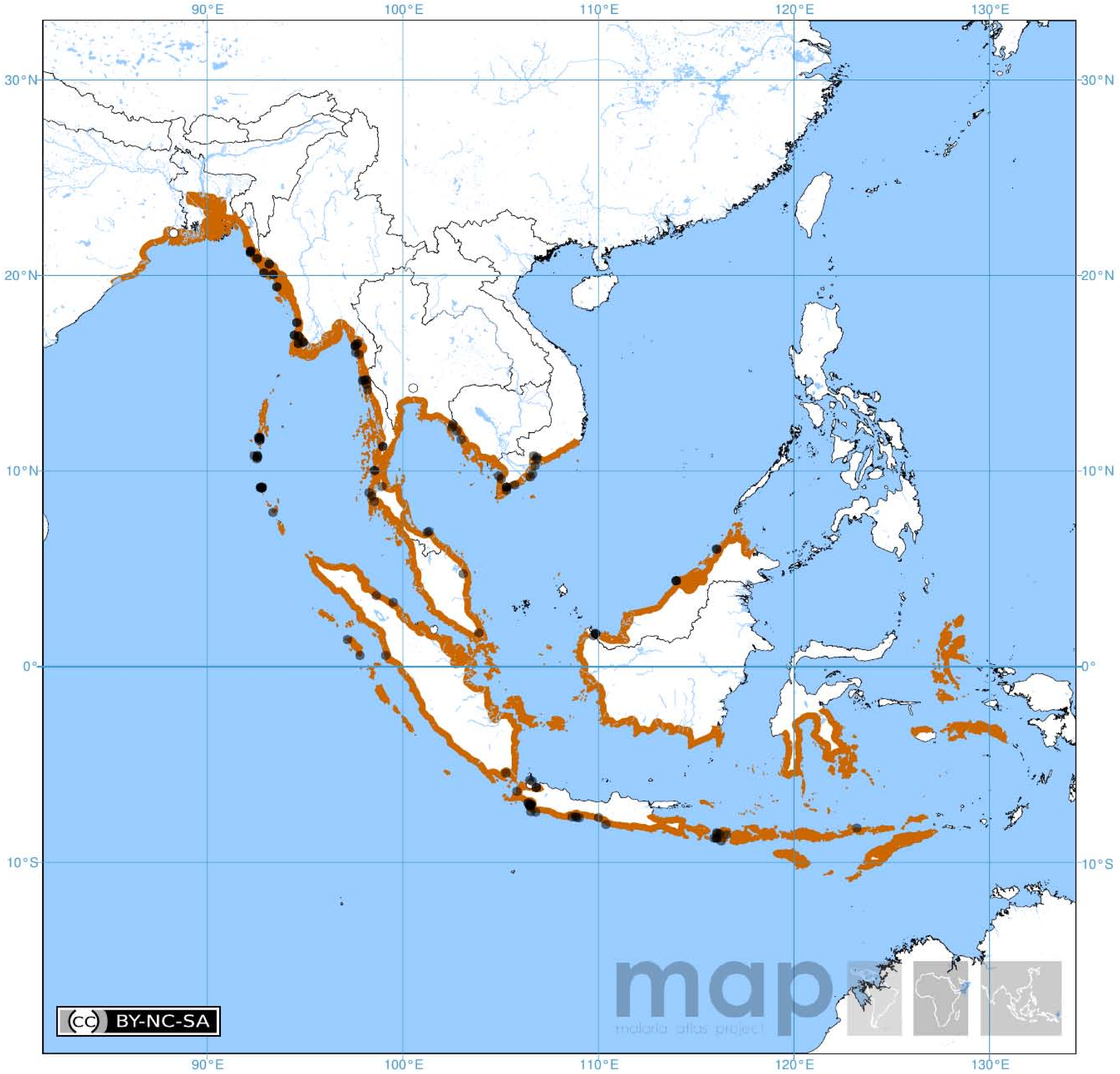

0 2500 5000

Kilometres

N

The 137 records for *Anopheles* (*Cellia*) *sundaicus*, were found in 7 countries between 1985 and 2006. There were 133 records of occurrence and 4 records of true absence.

Note: We are particularly keen to augment this map with occurrence records from Peninsular Malaysia, the east and west coasts of Sumatra and Borneo (Sabah, Sarawak and east and south Kalimantan), as well as, south Sulawesi, the Moluccas, Lesser Sunda Islands and the south coast of Java. Please e-mail map.vector@zoo.ox.ac.uk if you have any useful information to share on the distribution of this species complex.

Copyright: Licensed to the Malaria Atlas Project (MAP; www.map.ox.ac.uk) under a Creative Commons Attribution 3.0 License (http://creativecommons.org/).

Citation: Hay, S.I. *et al.* (2009). Developing global maps of the dominant *Anopheles* vectors of human malaria. *PLoS Medicine* **6**: in press.

EO range

Present

Absent
